# Integrated QTL and eQTL Mapping Provides Insights and Candidate Genes for Fatty Acid Composition, Flowering Time, and Growth Traits in a F_2_ Population of a Novel Synthetic Allopolyploid *Brassica napus*

**DOI:** 10.3389/fpls.2018.01632

**Published:** 2018-11-13

**Authors:** Ruijuan Li, Kwangju Jeong, John T. Davis, Seungmo Kim, Soonbong Lee, Richard W. Michelmore, Shinje Kim, Julin N. Maloof

**Affiliations:** ^1^Department of Plant Biology, University of California, Davis, Davis, CA, United States; ^2^FnP Co., Ltd., Jeungpyeong, South Korea; ^3^The Genome Center and Department of Plant Sciences, University of California, Davis, Davis, CA, United States

**Keywords:** *Brassica napus*, QTL, eQTL, fatty acid, flowering time, growth related traits

## Abstract

*Brassica napus* (*B. napus*, AACC), is an economically important allotetraploid crop species that resulted from hybridization between two diploid species, *Brassica rapa* (AA) and *Brassica olereacea* (CC). We have created one new synthetic *B. napus* genotype Da-Ae (AACC) and one introgression line Da-Ol-1 (AACC), which were used to generate an F_2_ mapping population. Plants in this F_2_ mapping population varied in fatty acid content, flowering time, and growth-related traits. Using quantitative trait locus (QTL) mapping, we aimed to determine if Da-Ae and Da-Ol-1 provided novel genetic variation beyond what has already been found in *B. napus.* Making use of the genotyping information generated from RNA-seq data of these two lines and their F_2_ mapping population of 166 plants, we constructed a genetic map consisting of 2,021 single nucleotide polymorphism markers that spans 2,929 cM across 19 linkage groups. Besides the known major QTL identified, our high resolution genetic map facilitated the identification of several new QTL contributing to the different fatty acid levels, flowering time, and growth-related trait values. These new QTL probably represent novel genetic variation that existed in our new synthetic *B. napus* strain. By conducting genome-wide expression variation analysis in our F_2_ mapping population, genetic regions that potentially regulate many genes across the genome were revealed. A *FLOWERING LOCUS C* gene homolog, which was identified as a candidate regulating flowering time and multiple growth-related traits, was found underlying one of these regions. Integrated QTL and expression QTL analyses also helped us identified candidate causative genes associated with various biological traits through expression level change and/or possible protein function modification.

## Introduction

Rapeseed, *Brassica napus* (*B. napus*), is one of the most important oilseed crops worldwide producing multi-purpose oil for food and biofuel. As the global requirements for rapeseed oil are growing rapidly, it becomes important to understand the control of *B. napus* oil content, oil composition, and growth including flowering time, plant height, etc., so that different varieties can be bred to meet the agricultural demand in different areas.

Canola was developed from rapeseed through plant breeding. As a source of edible oil, canola seeds must contain less than 2% erucic acid and less than 35 μmol/g glucosinolates. Erucic acid (C22:1) is a monounsaturated fatty acid (FA); high concentrations of erucic acid have been reported to be responsible for producing fatty deposits in the heart (Houtsmuller et al., 1970). Besides erucic acid and glucosinolate content, the composition of various FAs in the seed oil determines the economic value of rapeseed, including saturated FA palmitic (C16:0) and stearic (C18:0), monounsaturated FA palmitoleic (C16:1), oleic (C18:1) and eicosenoic (C20:1), and polyunsaturated FA: linoleic (C18:2) and linolenic (C18:3).

The biosynthesis of FA has been well-characterized in the model species *Arabidopsis thaliana* (Houtsmuller et al., 1970; Harwood, 1996), also a member of the Brassicaceae family. Acetyl-CoA carboxylase carries out the first committed step in FA *de novo* synthesis (Harwood, 1996). Then two enzyme systems are utilized in long-chain FA synthesis: the FA synthase and FA elongase complexes. In the plastid, the FA synthase complex catalyzes the sequential addition of two-carbon units to the growing acyl chain (Harwood, 1996). In the ER, production of very-long-chain (>18 carbon) FAs is catalyzed by the FA elongase complex, and β-ketoacyl-CoA synthase (KCS) is the condensing enzyme, whereas β-ketoacyl-ACP synthase works as the condensing enzyme in FA *de novo* synthesis (Kunst and Samuels, 2003). FA with double bonds are generated by FA desaturase (FAD), *FAD2, FAD3, FAD6*, *FAD7*, and *FAD8* are different enzymes responsible for FA desaturation (Ohlrogge and Browse, 1995). In addition, Arabidopsis FAE1 (Fatty Acid Elongase; KCS18) is the best characterized FA elongase; *fae1* mutants have a reduced level of very long chain FA and elongation activity deficiency (James et al., 1995).

Because of the critical role of FA in seed oil quality and uses, multiple studies have been conducted to understand the genetic regulation of FA composition in *B. napus* (Thormann et al., 1996; Burns et al., 2003; Qiu et al., 2006; Cao et al., 2010; Li et al., 2014; Wang et al., 2015; Gacek et al., 2016; Korber et al., 2016). Thormann et al. (1996) found two major quantitative trait loci (QTL) that explained 95% of the phenotypic variance observed for erucic acid (C22:1) and suggested that linolenic acid (C18:3) might be controlled by *FAD3*, which mediates the desaturation of linoleic acid (C18:2) to linolenic acid (C18:3) in the ER. Burns et al. (2003) used substitution lines to identify a major QTL on linkage group (LG) 8 affecting nine out of the eleven FAs they scored, including oleic (C18:1) and erucic acid (C22:1). This same QTL was also found to be associated with seed oil content, suggesting the pleiotropic effect of this loci or the presence of multiple QTL underneath (Burns et al., 2003). Besides the two major QTL, using a double haploid (DH) population segregating for oleic (C18:1), linoleic (C18:2), and linolenic acid (C18:3) content, loci on LG5 (Linkage Group), LG4, and LG14 were found harbor *FAD2* and *FAD3* genes (Hu et al., 2006). Mutations on FAD2 and FAD3 could affect levels of oleic (C18:1), linoleic (C18:2), and linolenic acid (C18:3) content (Hu et al., 2006; Peng et al., 2010; Yang et al., 2012).

Studies have also been carried out to understand the genetic control of *B. napus* flowering time, plant height, and other growth-related traits (?; Chen et al., 2007; Long et al., 2007; Ding et al., 2012; Wurschum et al., 2012; Raman et al., 2013; Shi et al., 2013; Fletcher et al., 2015; Li et al., 2016). Flowering time QTL with phenotypic effects have been mapped in several chromosomes including A03, A10, C02, C06, etc. (?; Wurschum et al., 2012; Fletcher et al., 2015). Among these detected loci, *BnFLC10*, a QTL on A10, was identified multiple times, which was also found associated with plant height and root weight (?; Long et al., 2007; Hou et al., 2012). The *FLC* (*FLOWERING LOCUS C*) gene located under this QTL was proposed to be the putative candidate gene (Hou et al., 2012; Fletcher et al., 2016). When expressed in Arabidopsis *B. napus FLC* orthologs functioned as a floral repressor and delayed flowering (Tadege et al., 2001).

While variation of many traits results from sequence polymorphism in the coding region, gene expression differences can also result in observable phenotypic differences. With the advance of high throughput gene expression profiling technologies, many studies have been conducted to examine the genetic control of genome wide transcript variation in plants such as Arabidopsis (Keurentjes et al., 2007; West et al., 2007; Lowry et al., 2013), maize (Holloway et al., 2011; Wang et al., 2018), rice (Wang et al., 2014; Kuroha et al., 2017), *B. rapa* (Graham et al., 2014), tomato (Ranjan et al., 2016), wheat (Samad-Zamini et al., 2017), and lettuce (Zhang et al., 2017). Expression QTL (eQTL) associated with each gene can be classified as *cis*-eQTL or *trans*-eQTL, based on the location of the eQTL relative to the location of the affected gene (Kliebenstein et al., 2006). An inventory of eQTL that regulate gene expression levels provide the necessary information required for identifying genes that control quantitative phenotypes (Kliebenstein et al., 2006). To this end, studies have been conducted to correlate eQTL results with phenotypic data to elucidate candidate genes, whose expression levels affect phenotypes (Shi et al., 2007; Yin et al., 2011; Song et al., 2014; Wang et al., 2014; Qu et al., 2016; Ranjan et al., 2016; Galpaz et al., 2018; Yu et al., 2018). Specifically, in *B. napus*, important genes involved in flavonoid synthesis and apetalous characteristic were revealed through analyses combining eQTL results with phenotypic data (Qu et al., 2016; Yu et al., 2018).

An important question in plant breeding is the extent to which related species or relatives can serve as sources of new genetic variation for crop improvement. *B. napus* is an allopolyploid formed by interspecies hybridization between ancestral diploid species having an A-like genome (present day *B. rapa*) and C-like genome (present day *B. oleracea*) (Allender and King, 2010). We generated one new synthetic *B. napus* strain and one line with introgression from *B. juncea* to determine whether they could provide novel genetic variation for FA composition and growth traits. Additionally, we used RNA-seq expression analysis to help identify candidate genes underlying trait QTL. Overall, we aimed to (1) build a high resolution genetic map; (2) identify QTL responsible for various phenotypic traits; (3) examine the genome-wide genetic regulation of gene expression levels; (4) find candidate genes connected with various phenotypic traits through either gene expression level changes or sequence variation. Our results will extend the current understanding of genetical genomics of *B. napus* and provide insights and candidate genes for several traits in *B. napus*.

## Materials and Methods

### Plant Materials, Phenotyping, and Calculation of Broad Sense Heritability

Two *B. napus* genotypes were created in this study. Da-Ae (AACC, Korea patent number: 10-1432278-0000, 2014.08.13) was developed by crossing *B. rapa* (AA) with *B. oleracea* (CC) (Supplementary Figure 1). Seven selfed seeds were harvested from the cross between *B. rapa* and *B. oleracea*, three of them germinated and flowered, with only one plant producing seeds. This single plant that produced seeds was used to create Da-Ae through six generations of selfing (Supplementary Figure 1). Da-Ol-1 (AACC) was made by crossing a *B. napus* (AACC) common cultivar in Korea “Nae-Han” with a *B. juncea* (AABB) cultivar “Mi-So” (Korea patent number: 10-2012-0011666, 2012.02.06) following the procedures described in Supplementary Figure 1. The F_2_ population was derived from a cross between the male parent Da-Ae and the female parent Da-Ol-1. Parental lines, F_1_s, and the F_2_ plants were sown in the greenhouse at the Jeungpyeong in South Korea in September 2015 and harvested in May 2016.

Data for 41 phenotypic traits was collected from the 166 F_2_ plants, including oil content, 14 oil composition traits, two flowering time related traits (days to flowering: the time from sowing until the first flower opened; days to bolt: the time from sowing date until an inflorescence bolt was first observed), plant weight, root weight, weight of one thousand seeds, above ground height to the 5th, 10th, and 15th branch, as well as time series growth data measured at four time points for plant width, leaf number, and lobe number, or five time points for plant height (Supplementary Table 1). The crude oil contents were measured from crushed rapeseeds by hexane extraction. Briefly, dry seed was crushed into powder, extracted with n-hexane, sonicated and centrifuged; the supernatant was filtered by passing through a layer of sodium sulfate and further extracted by n-hexane; the remaining hexane was evaporated in oven and the crude oil content was then determined. For gas chromatographic analysis, the methyl ester derivatives of FAs were prepared by the methylation procedure using boron trifluoride-methanol reagent (Ackman, 1998). The chromatographic analysis of lipids was performed using Agilent Technologies 7890A and FAME Mix C4-C24 (Supelco, United States) was used as standard fatty acid methyl ester (Eder, 1995).

Calculation of broad sense heritability was carried out using lmerTest (Kuznetsova et al., 2018) package in R. The variance for the F_2_ population was used as phenotypic variance. Environmental variance was calculated using biological replicates of Da-Ae and Da-Ol-1 measured from the same year, except for different FAs and crude oil contents, for which we used data from multiple years (because within-year replicate data is not available for these traits).

The code that was used to analyze phenotypic data and calculate broad sense heritability is available at: https://github.com/MaloofLab/Li-eQTL-2018/blob/master/scripts/phenotype-data-analysis.md.

### Time-Series Growth Model

In addition to using the individual time point measurements for the growth traits we also modeled growth using a Gompertz growth equation (Gompertz, 1825; Winsor, 1932) for plant height, plant width, and lobe number and a Weibull growth equation (Weibull, 1951) for leaf number. The modified form of Gompertz growth model with 4-parameter was used:

$$l_{t}=L_{\infty }+(L_{\infty }-\beta )e^{{-e}^{-k\left(t-l\right)}}$$

where l = length (or any other measure of size) and t = time. The four parameters are: K, the growth rate; L∞, the asymptotic length at which growth is zero; β, the lower asymptote; I, the day at the inflection point. The Weibull growth model is described by the equation:

$$l_{t}=L_{\infty }+(L_{\infty }-\beta ){e^{\left(-kt\right)}}^{\delta }$$

where l = length (or any other measure of size) and t = time. The four parameters are: L∞, the upper asymptote; β, the lower asymptote; k, the growth rate; δ, a parameter that controls the x-ordinate for the point of inflection.

Both the Gompertz and Weibull functions were fit to F_2_ data using the R package for Bayesian multilevel models using Stan, brms (Bürkner, 2017). Each model was fitted using 4 chains, each with 5000 iterations, the first 2500 of which were warmed up to calibrate the samples, resulting in a total of 10000 posterior samples. From the fitted model, we extracted growth model parameter coefficients for each F_2_ and then used them as function valued traits (FVT) for QTL mapping (Stinchcombe and Kirkpatrick, 2012).

### Total RNA Extraction, RNA-Seq Library Preparation, and Sequencing

For RNA sequencing, young leaf, flower, bolting tissue, 1cm silique, and 5 cm silique tissue were collected 77, 133, 189, 211, 208 days and 83, 104, 189, 204, 208 days, respectively, from Da-Ae and Da-Ol-1, three biological replicates were used except for bolting tissue, which there was no replicate for Da-Ae and only two replicates for Da-Ol-1. For the F_2_s, 5 cm silique tissue were collected from 166 plants.

Total RNA was extracted from the sample collected from both parents Da-Ae and Da-Ol-1 as well as the 166 F_2_s. RNA-seq libraries were prepared following the BrAD-seq protocol (Townsley et al., 2015). The constructed libraries were sequenced on illumina HiSeq 2500 at Teragen Etex as 100-bp strand specific paired end reads.

### Sequencing Data Preprocessing and Genome Mapping

Before mapping, raw sequencing reads were trimmed by removing adapter sequences, low quality sequences, and short read length sequences after clipping (length below 36 bp) using Trimmomatic v0.33 with parameters [ILLUMINACLIP:Bradseq_adapter.fa:2:30:10 LEADING:3 TRAILING:3 SLIDINGWINDOW:4:15 MINLEN:36] (Bolger et al., 2014). The quality of raw reads and trimmed reads was checked by fastQC software v0.11.5. To check the possibility of *B. juncea* B subgenome introgression into Da-Ol-1, a pseudo reference genome including *B. napus* (A and C subgenomes) (Chalhoub et al., 2014) and *B. juncea* B subgenome (Yang et al., 2016) was constructed and reads from three libraries of Da-Ol-1 were mapped to this pseudo reference genome using STAR v2.5.2b with zero mismatch [–outSAMtype BAM SortedByCoordinate; –twopassMode Basic; –outReadsUnmapped Fastx; –readFilesCommand zcat; –outFilterMismatchNmax 0]. Then reads uniquely mapped were used to calculate read depth across different chromosomes and unique mapping rate. For this analysis, one library from Da-Ae was used as a negative control. Due to the similar mapping rate and read depth distribution pattern between Da-Ae and Da-Ol-1 (Supplementary Figure 2), we concluded that there was no evidence of substantial introgression of the B subgenome into Da-Ol-1 and therefore decided to use the published genome sequence of *B. napus* v5 (Chalhoub et al., 2014) as reference genome for mapping. In brief, the trimmed high-quality reads were mapped to the pre-built genome index of the published genome sequence of *B. napus* v5 (Chalhoub et al., 2014) using STAR v2.5.2b with parameters [–outSAMtype BAM SortedByCoordinate; –quantMode TranscriptomeSAM GeneCounts; –twopassMode Basic; –alignIntronMax 15000; –outFilterIntronMotifs RemoveNoncanonical; –sjdbGTFtagExonParentTranscript Parent; –sjdbGTFfeatureExon CDS; –outReadsUnmapped Fastx; –readFilesCommand zcat] (Dobin et al., 2013). By including the GFF3 file (version 5) of the published *B. napus* genome sequence during mapping, both the alignment bam file and read count file for the annotated genes were obtained. The final sequencing and mapping results are summarized in Supplementary Table 2.

### Gene Expression Analysis Between Da-Ae and Da-Ol-1

Before expression analysis, TMM normalization (Robinson and Oshlack, 2010) implemented in edgeR was applied to correct for library size effect. Gene expression analysis between Da-Ae and Da-Ol-1 was then conducted using edgeR’s glm function with an FDR corrected *p*-value < 0.05 (Robinson and Smyth, 2007, 2008; McCarthy et al., 2012). The glm model was Gene read count ∼ Genotype ^∗^ tissue; genes with significant genotype and/or genotype by tissue effect were identified by conducting genewise statistical tests for corresponding coefficient(s). Gene ontology (GO) enrichment analysis was done by GOseq R package (Young et al., 2010) using custom scripts^1^. Results were visualized in heatmap using ggplot2 (Wickham, 2009) with custom scripts where -log10 of enriched *p*-values were indicated by color. Significance was determined based on *p*-value < 0.05 by GOseq.

### SNP Calling Between Da-Ae and Da-Ol-1

For SNP (Single Nucleotide Polymorphism) calling between Da-Ae and Da-Ol-1, the trimmed reads from each parent were merged into one FASTA file and mapping was conducted on the two separate files, respectively, using STAR v2.5.2b with parameters [–outSAMtype BAM SortedByCoordinate; –quantMode TranscriptomeSAM GeneCounts; –twopassMode Basic; –alignIntronMax 15000; –outFilterIntronMotifs RemoveNoncanonical; –sjdbGTFtagExonParentTranscript Parent; –sjdbGTFfeatureExon CDS; –outReadsUnmapped Fastx; –readFilesCommand zcat] (Dobin et al., 2013) (Bolting tissue was not used in SNP calling). The resulting alignment files were processed by a custom script to extract only uniquely mapped reads and PCR duplicates were removed as well. With this script, SNPs for Da-Ae and Da-Ol-1 versus the reference published *B. napus* genome (Chalhoub et al., 2014) were identified separately using Freebayes v0.9.21-7-g7dd41db (Garrison and Marth, 2012). The output vcf files from the two parents were processed using a custom script^2^ to retain only biallelic SNPs with high confidence using different parameters including QUAL score (greater than 40), read depth (greater than 10 and less than 1000), genotype quality (greater than 30) and tail bias (see scripts for details).

GATK v3.6 haplotype caller (McKenna et al., 2010) was also used for SNP calling between Da-Ae and Da-Ol-1 following the best practice for RNAseq short variant discovery. Briefly, uniquely mapped reads from both parents were processed to remove PCR duplicate using Picard^3^ followed by adding read group information, and indexing. Next, SplitNCigarReads developed specially for RNAseq was applied to reduce the number of called false variants due to inaccurate splicing of some of the read. Variant calling between Da-Ae and Da-Ol-1 was conducted on the resulting analysis-ready RNAseq read alignment files for each chromosome in parallel, and the 41 final vcf output were concatenated into one vcf file using CatVariants. The code that was used to perform each of these steps is available at: https://github.com/MaloofLab/Li-eQTL-2018/blob/master/scripts/SNP-calling.Rmd. Filtering of SNPs identified from GATK was also carried out using custom script^2^.

Only overlapped SNP sets between Freebayes and GATK were kept for genotyping of the F_2_s. snpEff v4.1 (Cingolani et al., 2012) was used for SNP annotation.

### Genotyping of the F_2_s and Genetic Map Construction

For F_2_ genotyping, Freebayes v1.0.2-33-gdbb6160 (Garrison and Marth, 2012) was used for parallel SNP calling of the 166 F_2_s on per chromosome level and the resulted output were merged into one vcf file. Segregation ratio were checked and SNPs with low quality and high missing rate were filtered.

Genetic linkage map construction was performed using SNPs with a missing rate less than 0.1 across F_2_ population. First, two-point recombination fraction between all pairs of markers was calculated in R package Onemap (Margarido et al., 2007) with the default values of LOD score 3 and maximum recombination fraction 0.50. Meanwhile, an in-house python tool madmapper^4^ was used to cluster markers into different linkage groups. Then, with the calculated two-point recombination information, markers within each linkage group were ordered using Rapid Chain Delineation algorithm. Next, markers with double crossovers in individual F_2_s were replaced with missing data and the ripple function was applied to further refine the order of makers within a linkage group. Last, the direction of several linkage groups was flipped according to the order of their markers on the physical map of the reference *B. napus* genome. Pairwise recombination and LOD plots were examined throughout the map construction process for quality check of the genetic linkage map. The code that was used to perform each of the above steps is available at: https://github.com/MaloofLab/Li-eQTL-2018/blob/master/scripts/genetic-map-construction.Rmd and https://github.com/MaloofLab/Li-eQTL-2018/blob/master/scripts/genetic_map_construction_2.md. Circos plot was made using R package circlize v0.4.3 (Gu et al., 2014).

### QTL Mapping

Quantitative trait locus mapping was performed using both interval mapping (scanone function in R/qtl) (Lander and Botstein, 1989) and composite interval mapping (CIM) (Zeng, 1994) methods implemented in R/qtl package (Broman et al., 2003). The experiment-wise 0.05 significance LOD threshold value was determined empirically for each trait from 1000 permutations (Churchill and Doerge, 1994). Compared to interval mapping, which investigates the association between phenotypic trait values and single marker intervals, CIM includes additional markers as covariates to reduce residual variance and control genetic variations of linked or unlinked markers beyond interval of interest (Broman et al., 2003). CIM has the advantage of better mapping resolution, however, due to the more complex model can sometimes have reduced sensitivity for small QTL as compared to interval mapping. So for each trait, if the QTL detected from the two methods overlapped with each other, CIM derived QTL and confidence intervals were recorded due to its higher precision, otherwise, unique QTL identified from both methods were kept. For traits with multiple QTL identified from interval mapping and CIM, two-way QTL mapping (scantwo function implemented in R/qtl) and multiple QTL mapping (fitqtl function implemented in R/qtl) were performed to test all QTL in the same statistical model to examine significance and identify epistasis. Any markers not significant using fitqtl were dropped. For each QTL, 95% Bayes credible interval was determined via the Bayesint function. Allelic effects were calculated using the fitqtl function. The code that was used to perform each of the above steps is available at: https://github.com/MaloofLab/Li-eQTL-2018/blob/master/scripts/QTL-eQTL-mapping.Rmd and https://github.com/MaloofLab/Li-eQTL-2018/blob/master/scripts/QTL_eQTL_mapping_2.md. Synteny analysis was conducted using SyMAP v4.2 (Soderlund et al., 2006).

### eQTL Analysis and Candidate Genes Identification for Phenotypic Traits

To obtain the e-trait (expression level of each gene) in the F_2_ population, the read count files generated from genome mapping for each F_2_ were processed and combined into a single file. Genes with detectable expression (expressed with greater than 10 CPM in at least 1/4 of the F_2_s) were transformed using variance stabilizing transformation implemented in DESeq2 package (Love et al., 2014). The strong batch effect propagated from RNAseq library preparation step was corrected by removeBatchEffect function implemented in limma package (Ritchie et al., 2015).

Expression QTL mapping was then conducted on the processed e-traits using the multiple imputation algorithm of interval mapping method (Lander and Botstein, 1989) in R/qtl package (Broman et al., 2003), and QTL associated with each e-trait was identified based on a LOD threshold value determined by 1000 permutations of 100 sampled genes at the significance level of 0.05. Using a custom script^5^^,^^6^, 95% Bayes credible interval(s) for each gene was determined and transformed to corresponding physical position on the reference *B. napus* genome (Chalhoub et al., 2014). Since the average physical space between genetic markers is 0.5 Mb, we added 0.25 Mb length to both ends of each eQTL interval to overcome the over precision of eQTL mapping when using bayeint() function [with parameter: expandtomarker = TURE; prob = 0.999], and the physical position of each gene was compared with their eQTL interval. For each gene, when their eQTL interval overlapped with their physical position, that eQTL was classified as a *cis*-eQTL, otherwise, a *trans*-eQTL was identified. R package qtlhot was used to identify *trans*-eQTL hotspot (Neto and Yandell, 2013). We note that while there is no replication of the F2s (since it is not possible), (1) the eQTL regression model is based on marker (or interval) genotype, (2) genotypes at each marker are randomly distributed across the field. Therefore, we have a high level of replication for the eQTL model and an appropriate design for detection of eQTL.

The 95% Bayes credible interval(s) for e-traits identified from above and the QTL interval for phenotypic traits obtained from QTL mapping step were compared with each other. Genes whose eQTL overlapped with a phenotypic trait QTL were identified candidate regulators for the corresponding phenotypic traits. For identification of candidate genes with possible protein function modification, genes underlying the 95% Bayes credible interval(s) for phenotypic traits with non-synonymous mutation(s) between Da-Ae and Da-Ol-1 were listed.

## Results and Discussion

**FIGURE 1:**
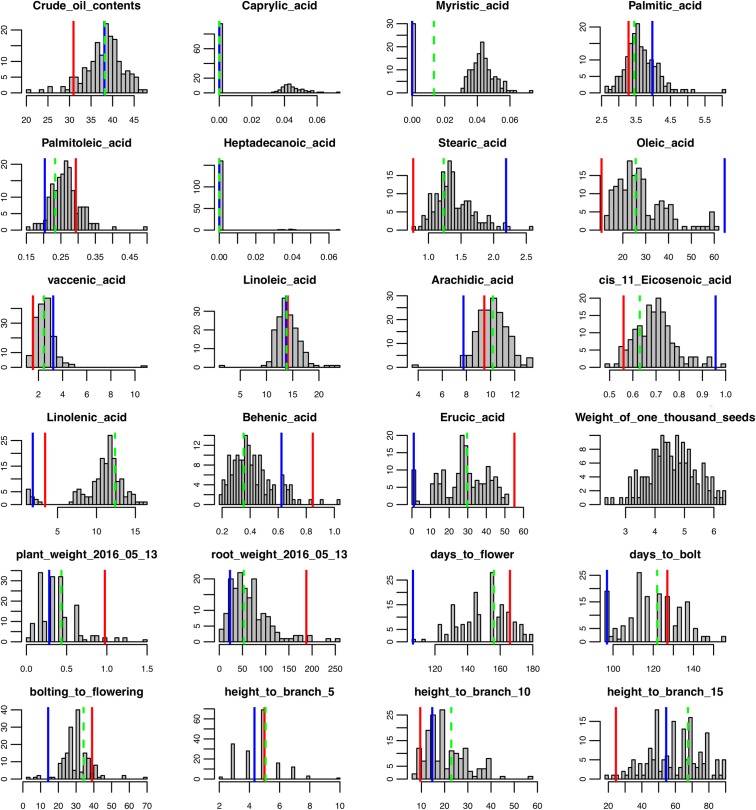
Phenotypic trait data distribution for the F_2_ population, parents, and the F_1_. Red lines indicate trait values for Da-Ae, blue lines indicate trait values for Da-Ol-1, and green dashed line indicate trait values for F_1_.

We synthesized two new strains of *B. napus* Da-Ae (AACC) and Da-Ol-1 (AACC). The synthetic allotetraploid Da-Ae is a Korean winter cultivar that flowers later and has seeds with high erucic acid (C22:1) content. Da-Ol-1 is a spring cultivar that takes a shorter time to flower and has seeds with high oleic acid (18:1) content (Figure 1). Da-Ol-1 also has introgression from *B. juncea* (AABB). To begin exploring the genetic basis of these differences we performed a gene expression analysis between the two genotypes. The results suggested that these phenotypic differences might be reflected in gene expression changes; for example, differentially expressed genes were enriched in GO terms related to lipid and FA processes (Supplementary Figure 3). In total, 15,098 genes were differentially expressed between Da-Ae and Da-Ol-1. The number of differentially expressed genes between genotypes we identified are higher than other studies (Teng et al., 2017; Li et al., 2018) because we included more (five) tissues types (see section “Materials and Methods”). It is also possible that the higher number of differentially expressed genes is because the two parental genotypes are adapted to different eco-environments (winter and spring). Among these differentially expressed genes, 1,701 were significant for the genotype by tissue effect, with lipid and FA related pathways that were differentially modulated between the two genotypes developmentally (Supplementary Figure 3).

### F_2_ Population Exhibits Phenotypic Variation for Several Correlated Seed Oil Traits and Growth Traits

To determine the genetic architecture underlying the phenotypic and gene expression differences in the parents, we analyzed an F_2_ population derived from a cross between Da-Ae and Da-Ol-1. We collected time series phenotypic data for growth traits and one-time measurements for seed, root, flowering traits, above ground height to the 5th, 10th, and 15th branch for 166 F_2_ plants (Supplementary Table 1). There was a wide range of phenotypic segregation, with a continuous normal quantitative distribution for most of the traits (Figure 1 and Supplementary Figure 4). One of the exceptions was caprylic acid (C8:0) that was not detected in either Da-Ae or Da-Ol-1; however, some of the F_2_ progeny did produce caprylic acid (C8:0) suggesting possible complementation of genes controlling this FA from the two parents. Myristic acid (C14:0) exhibited a normal distribution in the F_2_ plants; however, it was not detectable in some plants. Heptadecanoic acid (C17:0) data was not used for QTL analysis since it was undetectable in more than 150 plants. The distribution of linolenic acid (C18:3) was similar to caprylic acid (C8:0; low trait value for both parents, however, a higher normal distribution for some F_2_ plants) suggesting the underlying complementation function of regulatory or biosynthetic genes from Da-Ae and Da-Ol-1. Besides caprylic acid (C8:0) and linolenic acid (C18:3), transgressive segregation was observed in the F_2_ population for crude oil content, arachidic acid (20:0), and behenic acid (22:0).

**FIGURE 2:**
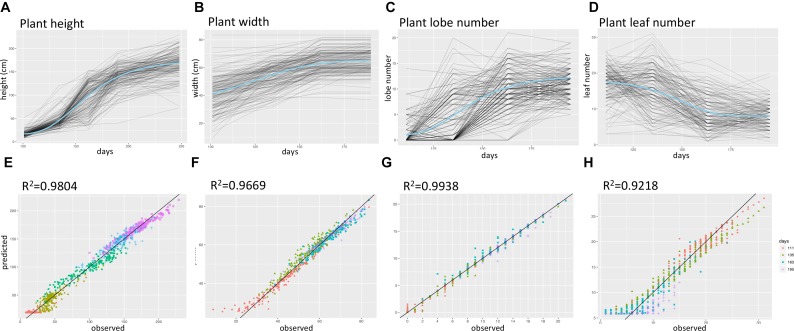
Summary of time-series growth model results. **(A–D)** Fitted growth model curves for plant height, lobe number, plant width, and leaf number. Blue curves represent the intercept values, and gray curves represent values for each F_2_ plant. **(E–H)** predicted versus observed trait values for plant height, lobe number, plant width, and leaf number, different colors represent data for different days after sowing.

The time series data was collected from multiple time points for plant width, leaf number, lobe number, and plant height (Supplementary Figure 4 and Supplementary Table 1). To capture parameters describing the shape of the growth curve for each of the four growth traits, we fit hierarchal Bayesian growth models to these time series data in which model parameters were allowed to vary for each F_2_ plant. These models each explained over 90% of the phenotypic variance observed (Figure 2). Supplementary Figure 5 shows the distribution of growth model parameters for each trait, with Hmax representing the highest value a F_2_ plant could reach for a specific trait (e.g., The maximum height of a plant) and k representing the growth rate. Inflection point I, in Gompertz model is the point on the curve at which the rate of growth reaches the maximum (or minimum) value and similarly, delta in Weibull model, is a parameter that controls the x-ordinate for the point of inflection (Goshu and Koya, 2013).

**FIGURE 3:**
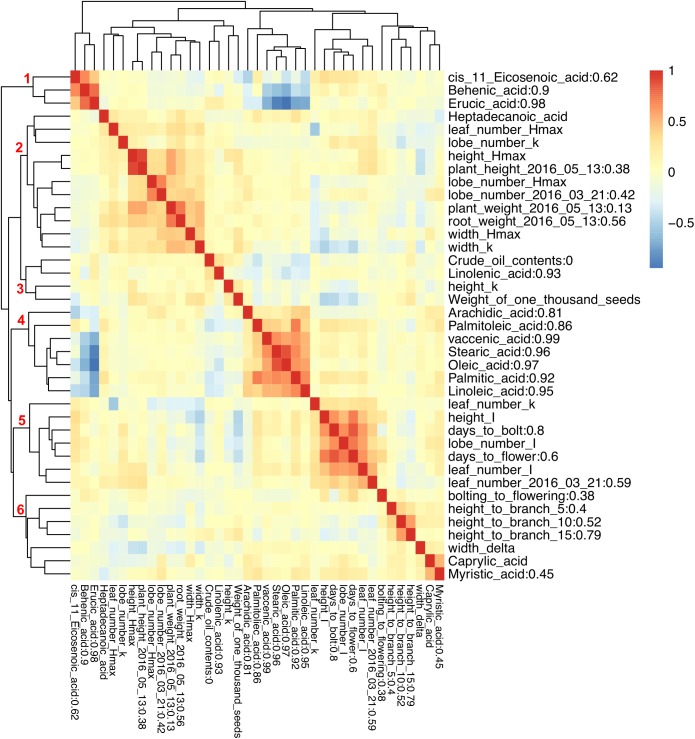
Pearson correlation between phenotypic traits and broad sense heritability. Different colors represent Pearson correlation values between phenotypic traits. Broad sense heritability values are shown as the numbers after trait names. Numbers on the branch indicate different groups formed due to high Pearson correlation.

To examine the correlation between phenotypic traits, pairwise Pearson correlation values were calculated, and hierarchical clustering was used to illustrate the relationships. Six clusters of positively correlated traits were identified (Figure 3). There was high negative correlation between two groups of FA composition traits, with erucic acid (C22:1), behenic acid (C22:0), and *cis*-11-Eicosenoic acid (C20:1) from group one being negatively correlated with arachidic acid (C20:0), palmitoleic acid (C16:1), vaccenic acid (C18:1), stearic acid (C18:0), oleic acid (C18:1), palmitic acid (C16:0), and linoleic acid (C18:2) from group four (Figure 3). While one type of FA can be converted into another type through either elongation or desaturation, the correlation pattern observed here illustrates a major branch point in the synthetic pathway. Caprylic acid (C8:0) and myristic acid (C14:0) were positively correlated with each other and did not show correlation with any other FA. Similarly, linolenic acid (C18:3) was not correlated with any other FAs. For growth and flowering related traits, we found that the maximum value for all growth traits was positively correlated (group two), and the time to the inflection point for all traits except for plant width, and the time to bolting and flowering were all positively correlated forming group five (Figure 3).

Broad sense heritability was also calculated: most FAs exhibited high heritability except for *cis*-11-Eicosenoic acid (C20:1) and myristic acid (C14:0) with a heritability below 0.8 (Figure 3); we were not able to calculate the heritability for caprylic acid (C8:0) and heptadecanoic acid (C17:0), because neither of them was detected in Da-Ae, Da-Ol-1, or the F_1_ line (Figure 1), from which we measured the environmental variability. The low heritability for different FAs including crude oil contents could be attributed to the over-estimation of environmental variability, because multiple year measurements were used for the calculation instead of multiple replicates from a single year. For flowering time and growth-related traits, environmental variability was calculated using multiple measurements from a single year. Thus, heritability values were comparable within different FAs or flowering time and growth-related traits, but not across these two groups of traits. Except for days to bolt, heritability for other flowering time and growth-related traits were all below 0.8 (Figure 3), which indicated that these traits are more complex and environment might have a stronger influence on them. Compared to our study, a similar level of broad sense heritability was reported for erucic acid (C22:1), oleic acid (C18:1), and linolenic acid (C18:3) in Ahmad et al. (2013); higher heritability values (greater than 0.85) were reported for flowering time related traits and plant height in Nelson et al. (2014) and Luo et al. (2017), respectively.

### SNPs Between Da-Ae and Da-Ol-1 and Generation of the Genetic Map

Using RNA-seq data generated from four tissue types (young leaf, flower, 1 and 5 cm long siliques) and three biological replicates, 63,981 SNPs were identified between Da-Ae and Da-Ol-1 (Supplementary Figure 6). While the gene density in subgenomes A and C was similar, a big difference in the density of SNPs was observed between Da-Ae and Da-Ol-1, with 69% of the total SNPs being mapped to subgenome A (Supplementary Figure 6). Similarly, a higher SNP density was observed in a *B. napus* diversity panel with a mean density of 1 SNP/15.44 kb for subgenome A and 1 SNP/29.89 kb for subgenome C (Qu et al., 2017). These findings are consistent with the report that subgenome A has a higher nucleotide diversity (Samans et al., 2017). The higher density of SNPs in subgenome A in our study could also be a result of the introgression of *B. juncea* into Da-Ol-1, which could introduce increased genetic diversity into subgenome A. The SNP density showed variation within each subgenome and each chromosome, reflecting the sequence coverage and/or the gene density across the genome (Supplementary Figure 6).

**FIGURE 4:**
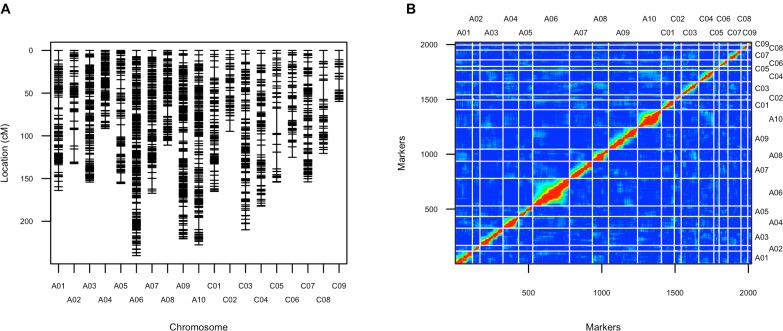
Genetic map of the F_2_ population. **(A)** Layout of markers on the genetic map; **(B)** Pairwise recombination and LOD score between markers shows the quality of this genetic map. Upper left part represents pairwise recombination fraction, and lower right part represents pairwise LOD scores.

After filtering SNPs with less than 90% coverage rate across the F_2_ population, from the above 63,981 SNPs, 2,021 SNPs were kept for construction of the genetic map. The genetic map recovered 19 linkage groups that represented the 10 chromosomes of subgenome A and the 9 chromosomes of subgenome C (Figure 4 and Supplementary Table 3). The total map length was 2,929 cM, with a 1.5 cM average distance between adjacent markers (Table 1). The number of markers per LG varied from 26 to 251, and the length of each LG varied from 59 to 240 cM, with large gaps of 29.5 cM and 22.3 cM on C05 and C08, respectively, due to low SNP and gene density (Table 1 and Supplementary Figure 6). There was a good collinearity between the constructed genetic map and the published *B. napus* reference physical map (indicated by the high Pearson correlation values between the genetic positions and physical positions for markers on different linkage groups), with only a few markers as exceptions (Supplementary Figure 7), these markers could indicate sites of genome rearrangements between the two parents that we used to compare to the published reference genome of *B. napus* (Chalhoub et al., 2014).

**Table 1:** Summary of the genetic map.

	Number of	Length	Average	Max
	mark	(cM)	spacing	spacing
**A01**	116	163.9	1.4	12.6
**A02**	52	132.5	2.6	19.2
**A03**	157	154.1	1.0	9.4
**A04**	107	91.4	0.9	6.3
**A05**	96	155.8	1.6	14.4
**A06**	251	240.2	1.0	7.4
**A07**	156	167.3	1.1	12.5
**A08**	110	110.9	1.0	12.1
**A09**	198	220.5	1.1	10.5
**A10**	163	227.6	1.4	14.2
**C01**	88	165.1	1.9	12.8
**C02**	47	94.7	2.1	13.1
**C03**	120	210.0	1.8	9.7
**C04**	103	182.2	1.8	13.7
**C05**	36	154.2	4.4	29.5
**C06**	60	125.1	2.1	12.2
**C07**	89	153.9	1.7	9.6
**C08**	46	120.6	2.7	22.3
**C09**	26	59.7	2.4	13.0
**overall**	2021	2929.9	1.5	29.5
**A subgenome**	1406	1664.3	1.3	19.2
**C subgenome**	615	1265.6	2.3	29.5


### QTL Mapping of Phenotypic Traits

We used two approaches for genome-wide QTL analysis, interval mapping and CIM. As expected (Jansen and Stam, 1994; Zeng, 1994), many QTL identified from both methods overlapped with each other, and CIM generally gave higher precision and stronger signal for the detected QTL (Supplementary Figure 8). However, there were several loci identified using interval mapping that were insignificant using CIM or *vice versa* (Supplementary Figure 8 and Table 2). To reconcile the results from the different methods, we tested the significance of all QTL discovered in a unified multiple qtl mapping model. Some of the phenotypic data showed deviation from normal distribution, such as caprylic acid (C8:0) and myristic acid (C14:0) (Figure 1). For these traits, we tried applying a two-part model to deal with zero-inflated value or using non-parametric interval mapping, both as implemented in R/qtl package (Broman et al., 2003), however, these methods did not find additional QTL beyond the IM or CIM approaches.

**Table 2:** List of QTL detected for phenotypic traits using CIM (composite interval mapping) or scanone (interval mapping) approach.

Trait	chr	confidence_interval	pos	LOD	flanking markers	R.square	additive.effect	Model
Arachidic_acid	A08	29.11–33.48	32	9.06	chrA08_5598684-chrA08_8712081	17.40%	0.71	CIM^∗^
Behenic_acid	A08	32.75–33.48	33	36.91	chrA08_8409348-chrA08_8712081	49.20%	-0.14	CIM^∗^
Behenic_acid	C03	167.26–180.81	175.1	6.7	chrC03_50371054-chrC03_56259623	5.90%	-0.04	CIM^∗^
Behenic_acid	A01	33.85–86.55	60	4.61	chrA01_1665566-chrA01_5352789	3.30%	-0.02	Scanone
*cis*_11_Eicosenoic_acid	A08	33.84–38.26	35.26	15.53	chrA08_8714266-chrA08_10042934	29.70%	-0.07	CIM^∗^
Crude_oil_contents	A03	93.51–100.9	98.54	7.06	chrA03_17859843-chrA03_18998089	9.20%	-1.69	CIM
days_to_bolt	A10	169.79–177.63	175.99	7.89	chrA10_14613931-chrA10_15142872	14.10%	-7	CIM^∗^
days_to_bolt	C06	71.3–87.94	85.72	7.21	chrC06_22643132-chrC06_31785732	11.40%	-7.5	CIM^∗^
days_to_flower	A10	182.84–187.74	183.94	8.64	chrA10_15367151-chrA10_15942430	16.50%	-7.4	CIM^∗^
days_to_flower	C06	66.69–91.24	82	5.7	chrC06_25340726-chrC06_32476288	11.60%	-7.6	Scanone
Erucic_acid	A08	37.96–40.38	38.87	49.18	chrA08_10082441-chrA08_10252345	51.20%	-13.1	CIM^∗^
Erucic_acid	C03	167.26–175.1	173	27.43	chrC03_50371054-chrC03_53755234	21.40%	-7.9	CIM^∗^
height_Hmax	A10	173.88–182.33	179	6.88	chrA10_14772310-chrA10_15394868	12.10%	-9.65	CIM^∗^
leaf_number_2015_12_28	A10	152.22–158.8	154.39	7.13	chrA10_13804529-chrA10_13882200	10.40%	-2.05	CIM
leaf_number_2016_03_21	A10	177.93–182.33	178.85	8.21	chrA10_15143193-chrA10_15394868	17.63%	-1.79	CIM^∗^
leaf_number_I	A10	152.22–193.33	176.72	5.06	chrA10_13804529-chrA10_16211022	13.00%	-9.13	Scanone
Linoleic_acid	A08	35.26–40.38	37.96	14.64	chrA08_9170855-chrA08_10252345	24.80%	1.7	CIM^∗^
Linoleic_acid	C03	167.26–180.81	172	9.27	chrC03_50371054-chrC03_56259623	9.90%	1.05	CIM^∗^
Linolenic_acid	A08	24–40.38	37.65	5.71	chrA08_4523415-chrA08_10252345	66.10%	0.51	Scanone
Linolenic_acid	C03	152.69–175.1	174	5.42	chrC03_35231068-chrC03_53755234	65.60%	-0.17	Scanone
lobe_number_2016_01_21	C06	43.49–112.9	68	4.24	chrC06_17988931-chrC06_34186889	11%	2.37	Scanone
lobe_number_I	C06	71.3–86.31	83.54	7.95	chrC06_22643132-chrC06_30253083	13.00%	-9.13	CIM^∗^
Myristic_acid	C06	65.05–105.08	86	4.32	chrC06_25440524-chrC06_33994242	11.30%	-0.01	Scanone
Oleic_acid	A08	36.09–40.38	38.57	30.4	chrA08_9205559-chrA08_10252345	46.10%	9.74	CIM^∗^
Oleic_acid	C03	182.77–187.14	185.35	15.95	chrC03_56699775-chrC03_57105562	33.40%	7.3	CIM^∗^
Palmitic_acid	A08	37.45–40.38	38.57	15.2	chrA08_10043009-chrA08_10252345	35.40%	0.39	CIM^∗^
Palmitic_acid	C03	167.26–176.61	174	8.52	chrC03_50371054-chrC03_53727555	13.70%	0.18	CIM^∗^
Palmitoleic_acid	C09	0–59.71	46.69	5.18	chrC09_7141985-chrC09_44446434	13.40%	-0.02	Scanone
plant_height_2016_05_13	A10	171.37–180.34	178.85	8.2	chrA10_14692950-chrA10_15177742	13.76%	-13.34	CIM^∗^
plant_width_2016_02_17	C02	70.26–81.65	75.99	8.82	chrC02_39235217-chrC02_42343010	11.97%	4	CIM^∗^
root_weight_2016_05_13	A10	169.79–173.41	172	11.53	chrA10_14613931-chrA10_14763016	17.70%	-23.95	CIM^∗^
Stearic_acid	A08	36.51–42.5	39	30.31	chrA08_9203680-chrA08_10849748	36.80%	0.25	CIM^∗^
Stearic_acid	C03	176.98–182.77	180.81	16.21	chrC03_54290917-chrC03_56699775	19.40%	0.18	CIM^∗^
Vaccenic_acid	A08	37.65–40.38	38.57	31.64	chrA08_10043132-chrA08_10252345	33.40%	0.72	CIM^∗^
Vaccenic_acid	C03	137.57–175.7	156	7.16	chrC03_29855026-chrC03_53746996	9.10%	0.37	Scanone


*The column “Model” specifies from which model the confidence intervals and other QTL information were derived. ^∗^ indicates overlapped QTL from both scanone and CIM were identified for the trait on the specific linkage group, otherwise unique QTL were identified.*

### Fatty Acid QTL

**FIGURE 5:**
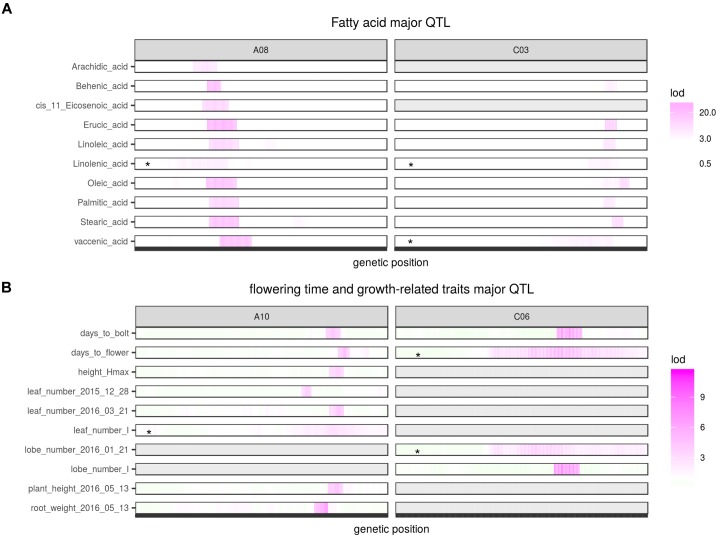
Major QTL identified for multiple traits. **(A)** Major QTL identified for different fatty acids; **(B)** Major QTL identified for flowering and growth-related traits. Asterisks indicate QTL identified from interval mapping (scanone) instead of composite interval mapping (CIM). Gray area indicate no QTL identified for that region.

Two QTL were identified on LG A08 and C03 as significant for almost every type of FA (Figure 5, Table 2, and Supplementary Figure 8). Arachidic acid (C20:0) and *cis*-11-Eicosenic acid (C20:1), different from other FAs, were only significant for the QTL on A08 (Figure 5, Table 2, and Supplementary Figure 8).

**FIGURE 6:**
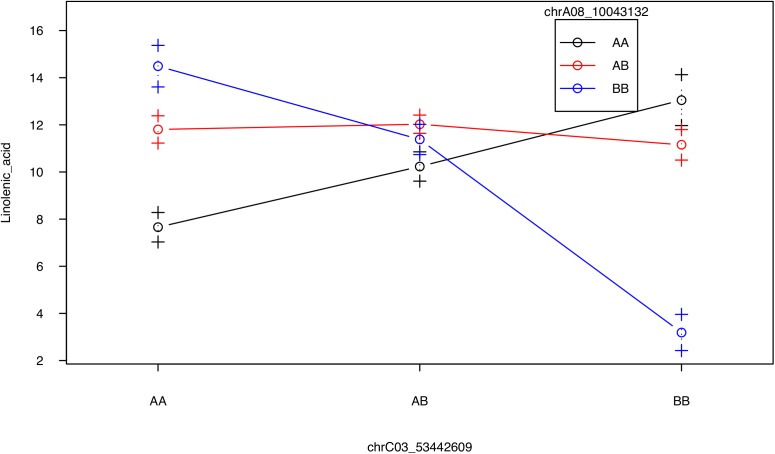
Strong epistatic effect between the two QTL for linolenic acid. Average linolenic acid levels for F_2_ plants with specific genotypes at the two major QTL on A08 and C03, represented by the two markers chrA08_10043132 and chrC03_53442609, respectively. AA, homozygous identical as Da-Ae; AB, heterozygous; BB: homozygous identical as Da-Ol-1.

No QTL were identified for linolenic acid (C18:3) in CIM although the two QTL on A08 and C03 were significant using interval mapping (Figure 5, Table 2, and Supplementary Figure 8). Using a multiple marker mapping approach, a strong epistatic effect between these two loci was observed (Figure 6); this model explained 84% of the total phenotypic variance. The epistasis between A08 and C03 loci also explained their absence of significance in the CIM model, which canceled the effect of one QTL when the other was included as a covariate (Zeng, 1994). This is a new finding compared to previous studies, which reported a major QTL on A08 but missed the QTL on C03 (Wang et al., 2015; Gacek et al., 2016).

The phenotypic variance explained by the locus on A08 was greater than the locus on C03 for most FAs (Figure 5 and Table 2), suggesting either that the gene underneath the A08 interval is more important for FA synthesis or that the segregating polymorphisms affecting the A08 QTL have a larger effect on gene function. The variance explained by the identified QTL varied among different FAs (Table 2): for erucic acid (C22:1), oleic acid (C18:1), and linolenic acid (C18:3), the *R*^2^-value explained by these two major QTL was greater or close to 80%; for stearic acid (C18:0), the *R*^2^-value was around 70%; the identified QTL explained about 50% of the phenotypic variance for behenic acid (C22:0), linoleic acid (C18:2), palmitic acid (C16:0), and vaccenic acid (C18:1), whereas only 17 and 29% of the phenotypic variance was explained for arachidic acid (C20:0) and *cis*-11-Eicosenic acid (C20:1), respectively, which had low heritability as well (Figure 3).

Besides FA composition, others have reported that the A08 QTL identified for FA composition also affects oil content (Burns et al., 2003; Qiu et al., 2006; Cao et al., 2010; Li et al., 2014). In our study, only one locus on A03 was identified significant for crude oil content, which explained 9.2% of the phenotypic variance for this trait (Table 2). This suggests that there are separate genes controlling FA composition and oil content both underlying the A08 QTL. Under this scenario both genes would have segregating alleles in the populations previous analyzed but only the FA composition gene has segregating alleles in our population.

For the majority of the FA, Da-Ol-1 carried the allele increasing the trait value, and this direction was consistent with the observed phenotypic differences between the Da-Ol-1 and Da-Ae parents (Figure 1 and Table 2). One exception is arachidic acid (C20:0) where the Da-Ol-1 allele increased the trait value even though the Da-Ol-1 parent strain had lower levels than the Da-Ae parent. For behenic acid (C22:0) and erucic acid (C22:1) the Da-Ol-1 allele decreased the trait value, consistent with expectations based on parental phenotypes. Lastly, for crude oil content and *cis*-11-Eicosenic acid (C20:1) the Da-Ol-1 allele decreased the trait value even though the Da-Ol-1 parent had a larger phenotypic value than Da-Ae. That the Da-Ol-1 allele reduces behenic acid (C22:0), erucic acid (C22:1), and *cis*-11-Eicosenic acid (C20:1) was consistent with our finding that these three types of FAs clustered together and formed a negative correlation with the other six FAs controlled by the same two loci (Figure 3).

Since the report in Thormann et al. (1996), the two major loci on A08 and C03 have been consistently found significant for FA composition in both linkage mapping analyses and GWAS studies (Burns et al., 2003; Qiu et al., 2006; Cao et al., 2010; Li et al., 2014; Wang et al., 2015; Korber et al., 2016; Gacek et al., 2016). *BnaA.FAE1* and *BnaC.FAE1* on the A08 and C03 homeologous genome blocks (Qian et al., 2014; Supplementary Figure 9), with synteny to Arabidopsis *FAE1* (Qiu et al., 2006), are accepted as the causal genes underlying the two major QTL. The *FAE1* genes in *B. napus* were first cloned in 2001 (Han et al., 2001); their implication in FA composition has been demonstrated using multiple approaches including introducing mutations by TILLING (Wang et al., 2008), RNA interference (Peng et al., 2010), and EcoTLLING (Wang et al., 2010). On the published reference genome of *B. napus* (Chalhoub et al., 2014), they were annotated as *BnaA08g11130D* and *BnaC03g65980D*, respectively. Here, *BnaA08g11130D* was found under the QTL interval for seven types of FAs: palmitic acid (C16:0), stearic acid (C18:0), oleic acid (C18:1), linoleic acid (C18:2), linolenic acid (C18:3), vaccenic acid (C18:1), and erucic acid (C22:1); *BnaC03g65980D* was found within the 95% confidence interval for behenic acid (22:0), linoleic acid (18:2), and stearic acid (18:0).

We found three additional FA QTL beyond the major A08 and C03 loci. This is less than previous studies using DH population (Burns et al., 2003; Qiu et al., 2006; Cao et al., 2010; Wang et al., 2015). The lower number of loci identified here could be caused by the small population size that we used and/or the inability to obtain replicate plant measurements in F_2_ population (since the genotypes are not fixed). For the three additional loci that we identified, a locus on C06 was significant for myristic acid (C14:0) explaining 11.3% of the phenotypic variance, C09 exhibited a QTL with a R^2^ of 13.4% for palmitoleic acid (C16:1), and A01 carried a QTL with minor effect for behenic acid (C22:0). While Arabidopsis *FAD2* and *FAD3* homologs in *B. napus* were reported to be the genes responsible for unsaturated FA variation (Hu et al., 2006; Yang et al., 2012), we did not find these genes under our QTL regions. Instead, there were three Arabidopsis *FAD6* homologs (*BnaA08g12780D*, *BnaA0812800D*, and *BnaC03g67820D*) located around 1Mb away from the A08 and C03 QTL, which might be in genetic linkage with the two QTL we identified and responsible for the variation in unsaturated FAs (especially if there is some discordance between the physical and genetic maps in this region).

### Flowering Time and Growth-Related QTL

Two major QTL, one on A10 and one on C06 were identified for flowering time and bolting time, each explaining 10 to 15% of the phenotypic variance. At both loci Da-Ae carried the allele delaying flowering (Table 2). The flowering trait QTL on A10 was also identified as significant for or close to many growth-related traits, including height_Hmax (The maximum height of a plant), leaf_number_I (the time taken to get inflection point on the leaf number growth curve), root weight, as well as plant height and leaf number measured from single day (Figure 5 and Table 2). The phenotypic variance of growth traits explained by this QTL ranged from 10 to 17%, with the allele from Da-Ol-1 decreasing the phenotypic value for each trait (Table 2), i.e., the allele both shortened the time to flowering and slowed or reduced growth. In contrast, the flowering and bolting time QTL on C06, was only additionally significant for the lobe_number_I growth trait (Figure 5 and Table 2).

Plant width can be an important feature of plant architecture, while no QTL were identified for plant width FVT data, a QTL on C02 was found control the plant width data measured from a single day, with Da-Ol-1 carrying the allele increased the trait value (Table 2).

Among the many QTL and GWAS studies conducted on flowering time, plant height, and other growth-related traits (Quijada et al., 2006; Chen et al., 2007; Long et al., 2007; Ding et al., 2012; Wurschum et al., 2012; Raman et al., 2013; Shi et al., 2013; Fletcher et al., 2015; Li et al., 2016), several found the same QTL region that we identified (Quijada et al., 2006; Long et al., 2007; Hou et al., 2012; Fletcher et al., 2015). Quijada et al. (2006) located a QTL on A10 as the regulatory region for both flowering time and plant height, pointing out *FLC* as the candidate genes. Later, using DH population and its derived reconstructed F_2_ population between a European winter cultivar and a Chinese semi-winter cultivar, linkage mapping analysis identified *BnFLC10* as a good candidate for the flowering time variation between spring- and winter- type *B. napus* (Long et al., 2007). Consistent with these two previous findings, we found the Arabidopsis *FLC* gene homolog *BnaA10g22080D* is located in the interval of A10 QTL for days to bolt, height_Hmax, leaf_number_I, and plant_height_2016_05_13, suggesting the regulatory role of this gene in multiple aspects of *B. napus* development. The study conducted by Long et al. (2007) also identified a QTL cluster on C06 for data from winter cropped environment and hypothesized it as a regulator of floral transition (Long et al., 2007). In our study, several genes with missense variant or stop lost mutation between Da-Ae and Da-Ol-1 were found underlying the significant QTL on C06, including a gene encoding global transcription factor (*BnaC06g27050D*), genes encoding auxin response factor (*BnaC06g20640D*) and heat shock transcription factor (*BnaC06g29140D*) (Supplementary Table 4). Synteny analysis found no homology between the A10 and C06 QTL interval identified from our study (Supplementary Figure 9); the C06 locus might be different from the locus identified in Long et al. (2007).

Taken together, we found several new QTL besides the two major QTL on A08 and C03 for FA composition. Additionally, we found a new, strong epistatic interaction between the A08 and C03 QTL affecting linolenic acid (C18:3) levels (Figure 6). The several loci with minor effect that we identified could also represent new genes segregating in our population, and the *FAD6* homologs close to the two major QTL might affect the unsaturated FA levels, due to its genetic linkage with the two major QTL. For flowering time and growth-related traits, the identification of pleiotropic QTL on A10, which regulated multiple traits, including flowering/bolting time, plant height, root weight, and leaf number, is consistent with what was known in Arabidopsis that FLC functions as a master regulator for many traits (Deng et al., 2011).

### eQTL: *cis*-eQTL and *trans*-eQTL

To understand the gene expression regulation on a genome wide scale, eQTL mapping of the transcript abundance in the F_2_ population was performed for the 56,182 genes with detectable expression. We found that 22,693 genes had eQTL; 11,031 genes had *cis*-eQTL and 13,296 genes had *trans*-eQTL. Because some genes had multiple *trans*-eQTL, 15,213 *trans*-eQTL were identified, comprising 58% of all eQTL found. We found eQTL for 5,650 of the 7,413 genes differentially expressed between the parents in the late silique tissue analyzed in the F_2_s, indicating that for many genes the genetic basis for gene expression differences in the parents has been identified in our analysis. Interestingly, approximately half of the genes did not show eQTL. There are several possible reasons for this: (1) it is likely that for some genes there is no variation for expression level segregating in our population, and this idea is supported by the fact that 48,769 genes showed no expression differences between the parents; (2) the QTL mapping method does not have enough power to detect very small eQTL, especially in a relatively small population; (3) genes which showed expression variation in this population but with no eQTL identified could have meiotically unstable epigenetic modification (meiotically stable epigenetic modification should be detectable as *cis-*eQTL).

**FIGURE 7:**
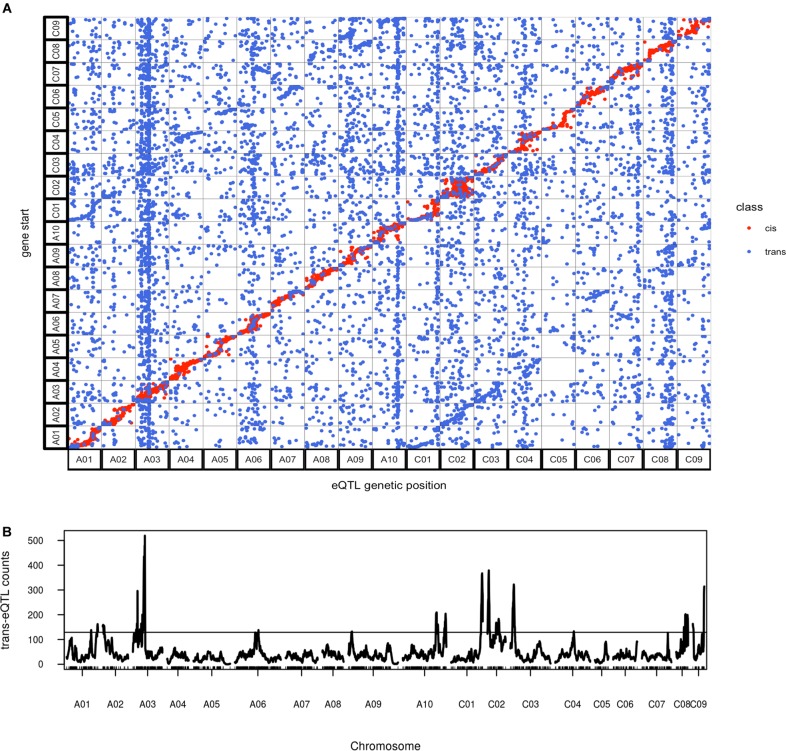
Overview of genome wide distribution of eQTL. **(A)** Alignment of eQTL genetic position against the physical position of its regulated transcript(s); **(B)** *trans*-eQTL hotspot distribution across the genome, the horizontal line represents threshold of 129 *trans*-eQTL per site calculated using qtlhot (Neto and Yandell, 2013), with *P*-value < 0.05.

As expected, *cis*-eQTL were evident as a diagonal line showing the correspondence between eQTL and transcript location (Figure 7). *Trans*-eQTL were non-uniformly distributed across the genome, with the highest number of *trans*-eQTL mapped to chromosome A03 (Figure 7). Twelve chromosomes were found to exhibit 20 *trans*-eQTL hotspots, only a few of which had significant over represented biological process GO categories (Supplementary Table 5). Among the several hotspots with significant enriched GO categories, genes regulated by the hotspot on A03 spanning from position 49 to 64 cM were significantly enriched for FA biosynthetic process (Supplementary Table 5). The A03 *trans*-eQTL hotspot was found significant for controlling phenotypic variance in erucic acid (C22:1) and oleic acid (C18:1) content; Da-Ol-1 alleles in this hotspot exhibited opposite effect compared to the major loci on A08 and C03, i.e., Da-Ol-1 alleles on A03 *trans*-eQTL hotspot increased erucic acid (C22:1) content and decreased oleic acid (C18:1). This hotspot is a *trans*-eQTL for the two *FAE1* genes underlying the two major FA composition QTL, *BnaA08g11130D* and *BnaC03g65980D* (Supplementary Table 5). We tested whether the A03 *trans*-eQTL hotspot might exert its effect on erucic acid (C22:1) levels by regulating expression of these two *FAE1* genes; however, the expression levels of *BnaA08g11130D* and *BnaC03g65980D* were not significant predictors of erucic acid (C22:1) or oleic acid (C18:1; via linear regression). Besides the two *FAE1*, over 40 genes with FA synthesis, metabolic, or storage GO terms were found to be targets of this A03 *trans*-eQTL hotspot (Supplementary Table 5), however, no single gene showed high expression level correlation with erucic acid (C22:1) or oleic acid (C18:1) content. This result suggested that this A03 *trans*-eQTL locus might have a minor effect on FA levels through multiple targets, which requires further investigation. Another *trans*-eQTL hotspot, A10 173-185cM overlapped with the pleiotropic QTL for flowering/bolting time, plant height, root weight, and leaf number (Supplementary Table 5 and Table 2). Combined with the fact that no enriched GO categories were found for genes regulated by this region, our study supports the idea that this QTL on A10 has a pleiotropic effect that regulates many aspects of plant development (Fletcher et al., 2015, 2016). Such result is also consistent with the finding in Arabidopsis that the FLC protein binds to over 500 sites located throughout the genome (Deng et al., 2011). It is highly likely that the *FLC* gene under this A10 QTL in *B. napus* has the similar regulatory role.

In addition to *cis*- and *trans*- eQTL, we also asked whether there was specificity in the *trans* regulation between the two genomes: are sub-genome A eQTL more likely to regulate targets in the A or C subgenome? Given that the A and C subgenomes were estimated to have been separated for four million years (Yang et al., 2016), we hypothesized that there would be evidence for specificity within sub-genomes. In cases where we did find evidence of inter-subgenome regulation would an eQTL from one of the subgenomes target homeologous regions in its alternate subgenome? As a result, we found 30% of the eQTL on both A and C subgenomes regulated genes on the alternate subgenome and an enrichment of regulation of homeologous regions was observed, which was revealed by the two lines parallel to the diagonal in Figure 7. Such result suggested the interaction between the two subgenomes, where genes on one subgenome act as *trans*- regulator of genes on the other subgenome, especially for their homeologous regions. However, these signals could also represent instances of *cis*-eQTL in one subgenome appearing to be *trans*-eQTL on the other, which could be an artifact of reads mapping to the wrong subgenome. That is, a true *cis*-eQTL on subgenome A could cause an apparent *trans*-eQTL at the homeologous region of subgenome C if some A reads mapped to the homeologous region of C. To test the proposed hypothesis, additional analysis is required, but the potential mapping problem should be firstly solved. It should also be noticed that our population was generated from a cross between a recent synthetic allopolyploid and a line with introgression from *B. juncea*, so caution should be taken when comparing any results to our study.

### Candidate Regulatory Genes From Integrated QTL and eQTL Results

By integrating QTL and eQTL results, we identified candidate genes underlying or associated with multiple QTL. We used three types of genes: (1) *cis*-coding candidates are genes with predicted amino acid changes that underlie a QTL peak (Supplementary Table 4); (2) *cis*-regulator candidates are genes with *cis*-eQTL (i.e., whose expression level is controlled in *cis)* that underlie a QTL peak (Supplementary Table 6); and (3) *trans*-eQTL target candidates are genes whose annotation is consistent with the trait being considered and whose expression is regulated by a *trans*-eQTL that underlies a QTL peak (Supplementary Table 7). In this later case, it is the genetic variation in the *trans*-eQTL, not the *trans*-eQTL targets that would underlie the QTL.

### Fatty Acid Regulatory Genes

Our integrated QTL and eQTL analyses located many *cis*-coding and/or *cis*-regulator candidate genes for different FAs. Among these genes, several were predicted to be involved in FA synthesis and metabolism.

**FIGURE 8:**
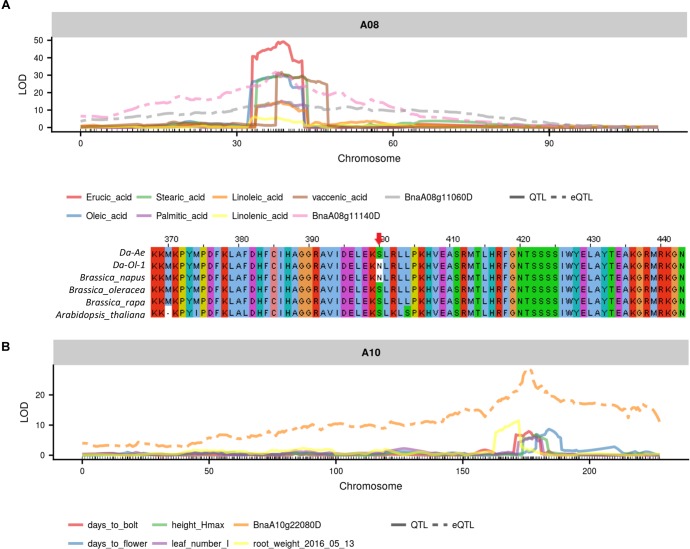
Example candidate genes identified from integrated QTL and eQTL analysis. **(A)** eQTL of *BnaA0811140D* and *BnaA08g11060D* overlapped with trait QTL for seven types of fatty acid; **(B)** *BnaA0811140D* has missense mutation in Da-Ol-1 relative to other species (Choi and Chan, 2015); **(C)** eQTL of *BnaA10g22080D* overlapped with trait QTL for flowering time and multiple growth-related traits.

From the two major QTL regions on A08 and C03, *BnaA08g11140D* was found to be both a *cis*-coding candidate and a *cis*-regulator candidate for seven types of FAs (Figure 8 and Supplementary Table 8). *BnaA08g11140D* had a missense deleterious mutation in Da-Ol-1 (Figure 8 and Supplementary Table 8), such result is consistent with *BnaA08g11140D* annotation that this gene encodes KCS17, an important enzyme catalyzing FA elongation. Another FA biosynthesis related gene *BnaA08g11060D* was also found to be a candidate *cis*-regulator for the seven types of FAs (Figure 8 and Supplementary Table 8). *BnaA08g11060D* is a squalene synthase 1 homolog and the inhibition of its protein product suppressed FA biosynthesis in rat hepatocytes (Hiyoshi et al., 2003). Besides these two genes, one *FAD6* homolog on A08, *BnaA08g12780D*, showed high expression level correlation with several FAs including erucic acid (C22:1) and oleic acid (C18:1), suggesting its possible regulatory role in affecting FA contents, although the eQTL of this gene was several cM away from the QTL for FAs.

For the behenic acid (C22:0) QTL on A01, *BnaA01g03770D* was identified as a *cis*-coding candidate; *BnaA01g07910D* and *BnaA03g37760D* were found to be associated with behenic acid (C22:0) and crude oil content, respectively, as both *cis*-coding and *cis*-regulator candidates (Supplementary Table 8). *BnaA01g07910D* encodes Enoyl-CoA hydratase; *BnaA03g37760D* is a beta-ketoacyl synthase homolog gene. Compared to other FAs, myristic acid (C14:0) and palmitoleic acid (16:1) had relatively large QTL intervals and small LOD scores (Table 2). *BnaC06g28980D*, a lipid metabolism related gene, was identified as a *cis*-regulator candidate for myristic acid (C14:0) (Supplementary Table 8).

As for candidate *trans*-eQTL target, *BnaA08g27750D*, an Arabidopsis KCS2 homolog, was associated with multiple FAs through gene expression (Supplementary Table 8). It would be helpful for understanding *B. napus* FA biosynthesis if we could locate the upstream regulatory gene (possibly transcription factor(s) identified as *cis*-coding and/or *cis*-regulator(s) for corresponding trait) in the A08 QTL region for *BnaA08g27750D*. This gene is also an example of inter-subgenome regulation that the gene eQTL is located on the homeologous region (C03) relative to the transcript location (A08), although the possibility that mis-mapping play a role here could not be excluded.

### Flowering Time and Growth-Related Traits Regulatory Genes

On the A10 QTL, our integrated QTL and eQTL analyses suggested that the underlying *FLC* gene homology *BnaA10g22080D* might work as a master regulator for multiple traits. Such finding was consistent with studies in *B. napus* (Hou et al., 2012; Fletcher et al., 2015, 2016) and Arabidopsis (Deng et al., 2011). In the past, others found that the allelic diversity caused by MITE insertion/deletion upstream of the *FLC* gene on A10 could result in *B. napus* winter and spring genotypes differentiation (Hou et al., 2012). Retroelement insertion in the first exon of this *FLC* gene creating truncated protein could also cause flowering time variation (Fletcher et al., 2016). We found that *BnaA10g22080D* expression was regulated by a *cis*-eQTL, with the allele from Da-Ol-1 decreasing the gene expression level, and this *cis*-eQTL co-localized with QTL for flowering/bolting time and multiple growth-related traits (Figure 8 and Supplementary Table 8). This would predict that plants inheriting the Da-Ol-1 allele would flower earlier, consistent with the phenotypic data. This result, together with the observation that this gene was underneath the *trans*-eQTL hotspot possibly regulating many aspects of plant development as well as reports from other studies, strongly suggested *BnaA10g22080D* to be a causative candidate gene affecting flowering time and many other growth-related traits in *B. napus*. Besides this *FLC* gene, *BnaA10g21380D* underlying the QTL for plant height, root weight, leaf number and time to bolt, had a missense mutation and its Arabidopsis homolog prevented initiation of lateral roots (Supplementary Table 8). Another candidate is *BnaA10g22000D*, whose Arabidopsis homolog mutants had defects in shoot and primary root growth (Supplementary Table 8).

For the C06 QTL (Figure 5), several genes underlying were found to be *cis*-coding and/or *cis*-regulator candidates (Supplementary Table 8). Among them, two auxin response factor coding genes *BnaC06g20640D* and *BnaC06g27170D* were found to be *cis*-regulator candidates; *BnaC06g20640D* had missense mutation making this gene a *cis*-coding candidate as well (Supplementary Table 8). A heat shock transcription factor coding gene *BnaC06g29140D*, identified as a *cis*-coding candidate, also shows missense mutation. We also identified one early-responsive to dehydration stress protein coding gene homolog, *BnaC06g30620D*, as a *cis*-coding candidate (Supplementary Table 8). Such finding suggests a connection between flowering time and drought tolerance level, which requires further investigation.

*Trans*-eQTL targets for flowering time trait were enriched for AGL3 binding site motif, which could possibility be regulated by the *FLC* gene *BnaA10g22080D* underlying the A10 QTL (Huang et al., 1995). The expression level of *BnaC06g29980D* and *BnaC04g35060D*, two MADS-box transcription factor coding genes, were found correlated with multiple traits including flowering time, plant height, and leaf number (Supplementary Table 8).

## Conclusion

In summary, using RNA-seq data of an F_2_ population made from crossing one synthetic *B. napus* genotype and one line with introgression from *B. juncea*, we constructed a high-resolution genetic map that facilitated fine mapping of phenotypic traits involved in many aspects of plant development, as well as genome-wide gene expression levels. Several major QTL identified as polymorphic between these new lines were already known from prior *B. napus* studies, indicating either that the polymorphism at those loci predate the formation of our new synthetic *B. napus* or that those loci are common targets for selection in *B. rapa*. However, fine mapping linkage analysis helped us identified several new QTL explaining the different FA levels, besides the two major QTL that were consistently found in other studies. Fine mapping using multiple markers helped us identify a strong epistatic interaction between the two major QTL on A08 and C03 for the first time, explaining variations in linolenic acid (C18:3) level. Being the first study examining the genome-wide genetic control of gene expression levels, we detected 20 *trans*-eQTL hotspot clusters, which regulate many genes across the genome. By integrating QTL and eQTL mapping results, we were able to locate candidate regulatory genes, which potentially affected various phenotypic traits through expression level changes and/or protein function modification. Among the several located candidates, our data, together with previous findings, suggested that the *FLC* homolog on A10 might function as a master regulator, affecting many aspects of plant development.

## Data Availability Statement

The raw sequence files generated for this study can be found at NCBI GenBank under the accession PRJNA470539.

## Author Contributions

JM, RM, and ShK conceived and designed the experiment. KJ and SL conducted sample collection, phenotyping and RNA-seq library preparation. RL, JD, SeK, and JM carried out data analysis. RL, JM, and RM wrote the manuscript. All authors have read and approved the manuscript.

## Conflict of Interest Statement

The authors declare that the research was conducted in the absence of any commercial or financial relationships that could be construed as a potential conflict of interest.

## Footnotes

1 https://github.com/MaloofLab/Li-eQTL-2018/blob/master/scripts/gene-expression-analysis.md

2 https://github.com/MaloofLab/Li-eQTL-2018/blob/master/scripts/SNP-calling.Rmd

3 http://picard.sourceforge.net/

4 http://cgpdb.ucdavis.edu/XLinkage/MadMapper/

5 https://github.com/MaloofLab/Li-eQTL-2018/blob/master/scripts/QTL-eQTL-mapping.Rmd

6 https://github.com/MaloofLab/Li-eQTL-2018/blob/master/scripts/QTL_eQTL_mapping_2.md
