# Supplementary material for: Integrated QTL and eQTL Mapping Provides Insights and Candidate Genes for Fatty Acid Composition, Flowering Time, and Growth Traits in a F2 Population of a Novel Synthetic Allopolyploid Brassica napus
Source: Front Plant Sci. 2018 Nov 13;9:1632. doi: 10.3389/fpls.2018.01632 (PMC6243938; doi:10.3389/fpls.2018.01632)
Supplement: Supplementary file 9 [file Image_1.pdf]

Supplementary Material

Integrated QTL and eQTL mapping provides insights and candidate genes for fatty acid composition, flowering time, and growth traits in a F_2 population of a novel synthetic allopolyploid *Brassica napus*

Ruijuan Li[1†], Kwangju Jeong[2†], John T. Davis[1], Seungmo Kim[12], Soonbong Lee[2], Richard W. Michelmore[3], Shinje Kim[2*], Julin N Maloof[1*]

† These authors contributed equally to this work.

*** Correspondence:** Julin N. Maloof: jnmaloof@ucdavis.edu; Shinje Kim: sjekim@fnpco.com

Supplementary Figures and Tables

Supplementary Figures

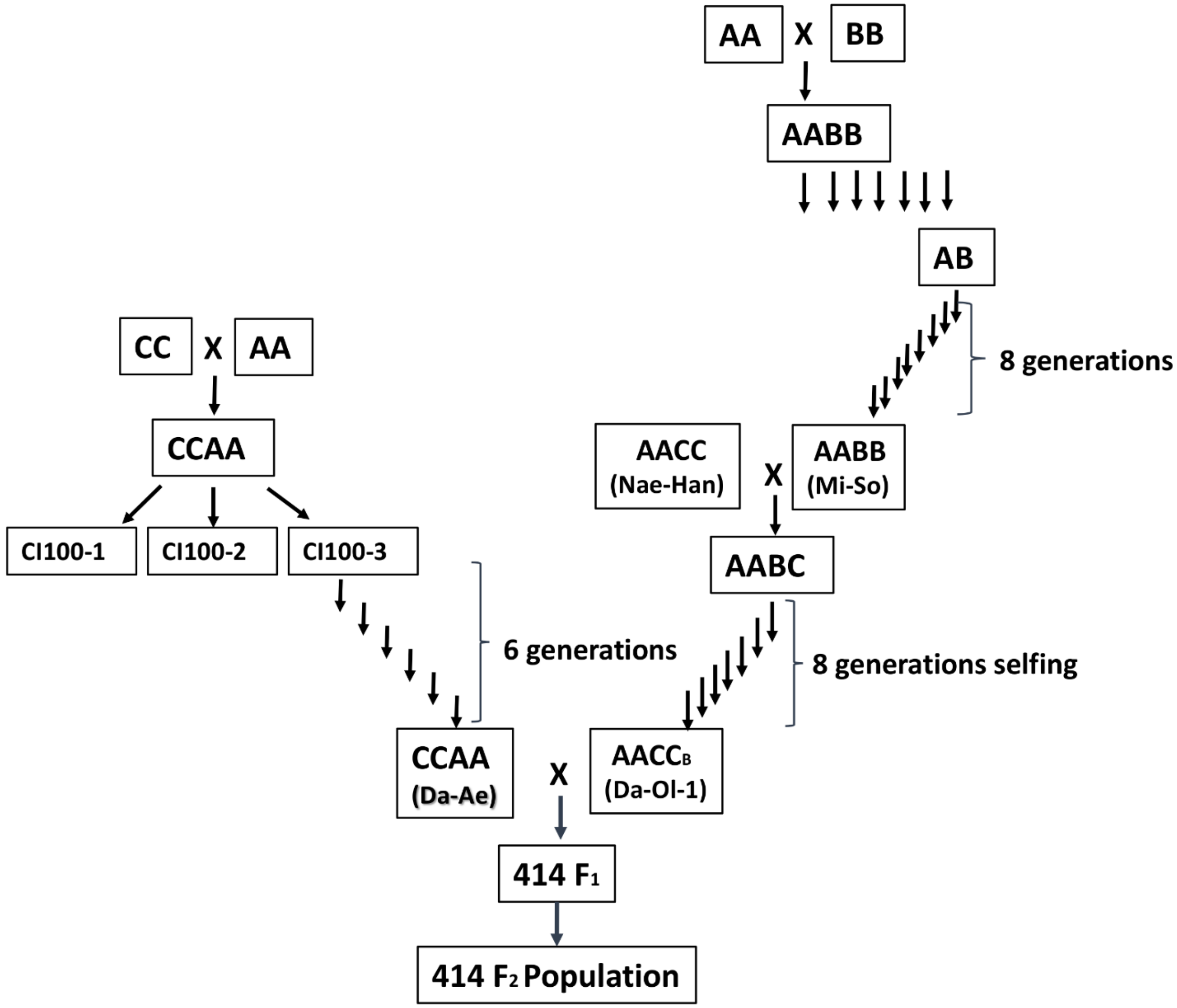

Supplementary Figure 1. Crossing scheme for Da-Ae, Da-Ol-1, and the F_2 population.

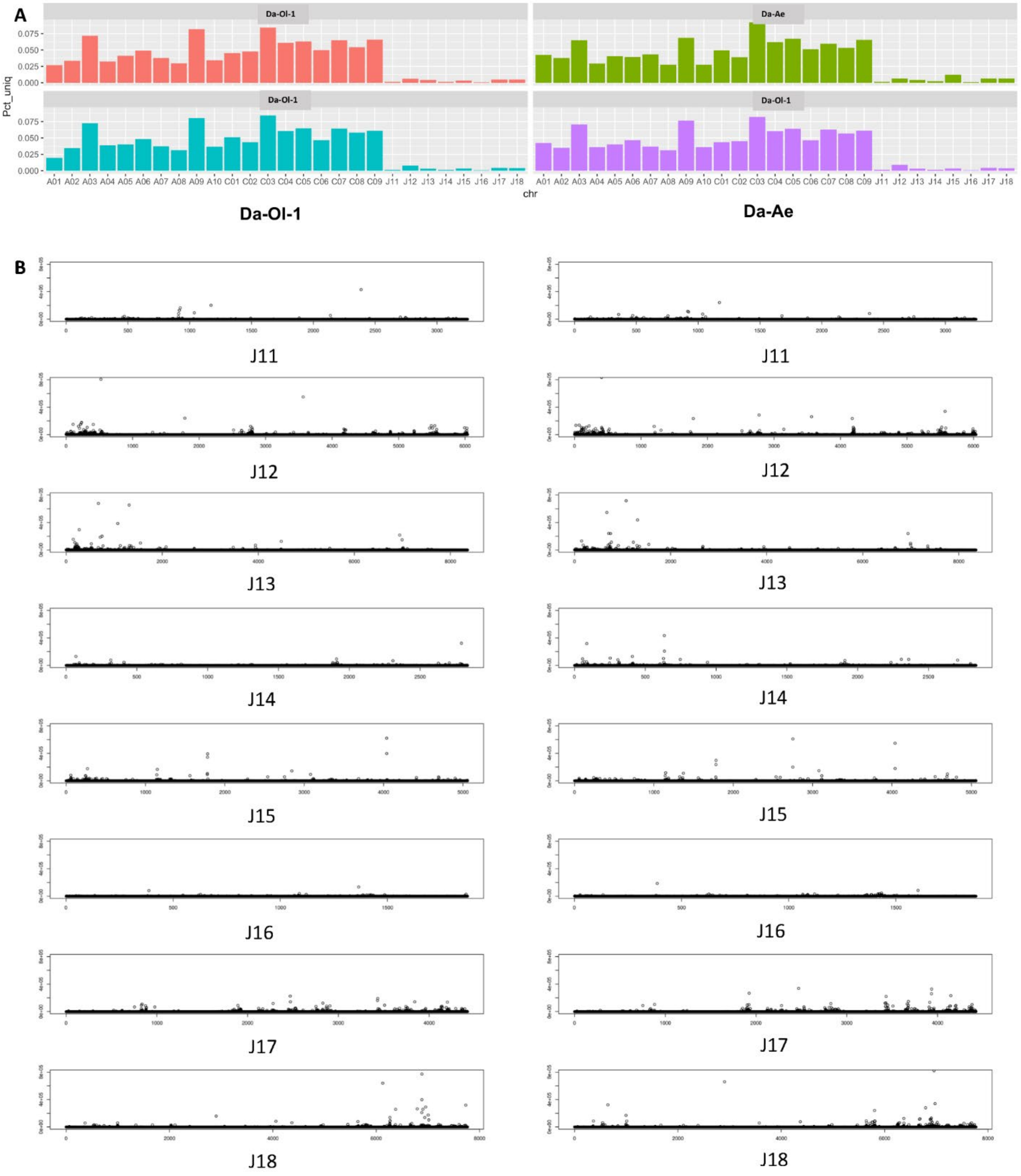

Supplementary Figure 2. Checking for possible *B. juncea* B-subgenome introgression into Da-Ol-1. (A) Percent of uniquely mapped reads (with zero mismatch) to the pseudo-reference genome containing A and C subgenomes of *B. napus* genome v5 (Chalhoub et al., 2014) and B-subgenome of *B. juncea* (Yang et al., 2016). A total of 2% of the reads were uniquely mapped to B-subgneome for Da-Ol-1, with the remaining 98% mapping to *B. napus* genome v5; for the negative control Da-Ae, a total of 3% of the reads were uniquely mapped to B-subgneome; (B) For reads that were mapped to B-subgenome in (A), the distribution of read mapping depth per Mb on B-subgenome are similar between Da-Ol-1 (left) and Da-Ae (right).

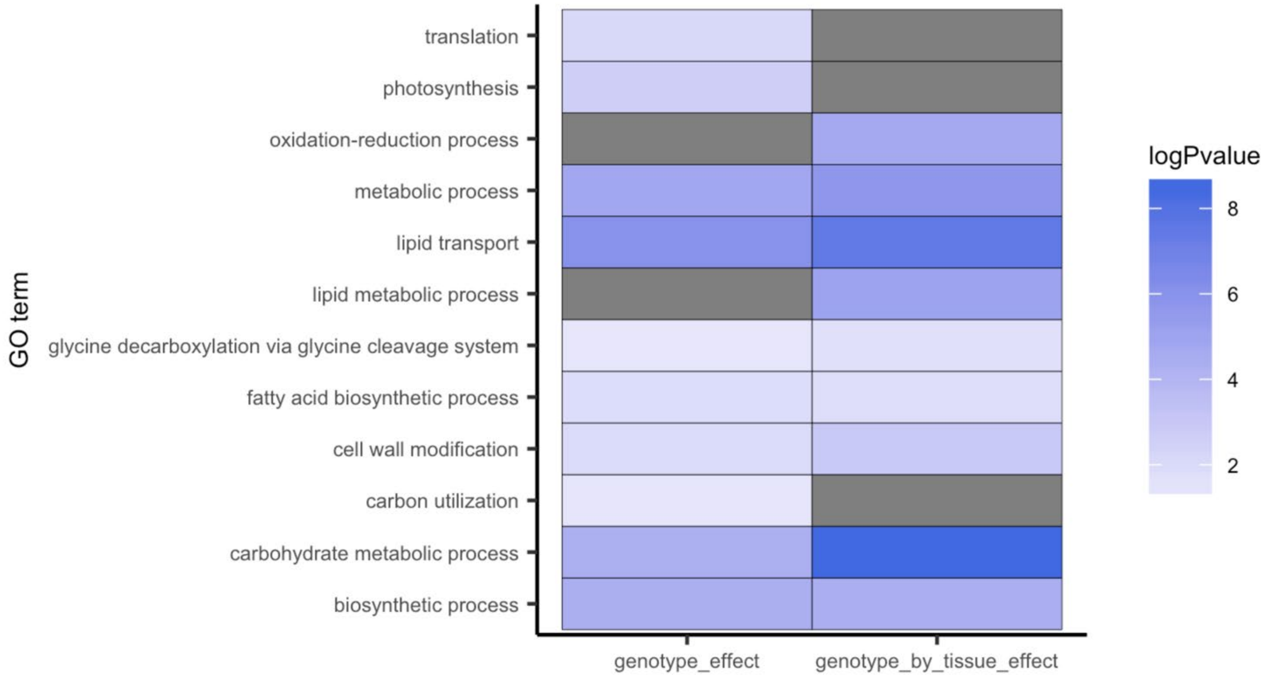

Supplementary Figure 3. Enriched gene ontology terms for differentially expressed genes between Da-Ae and Da-Ol-1.

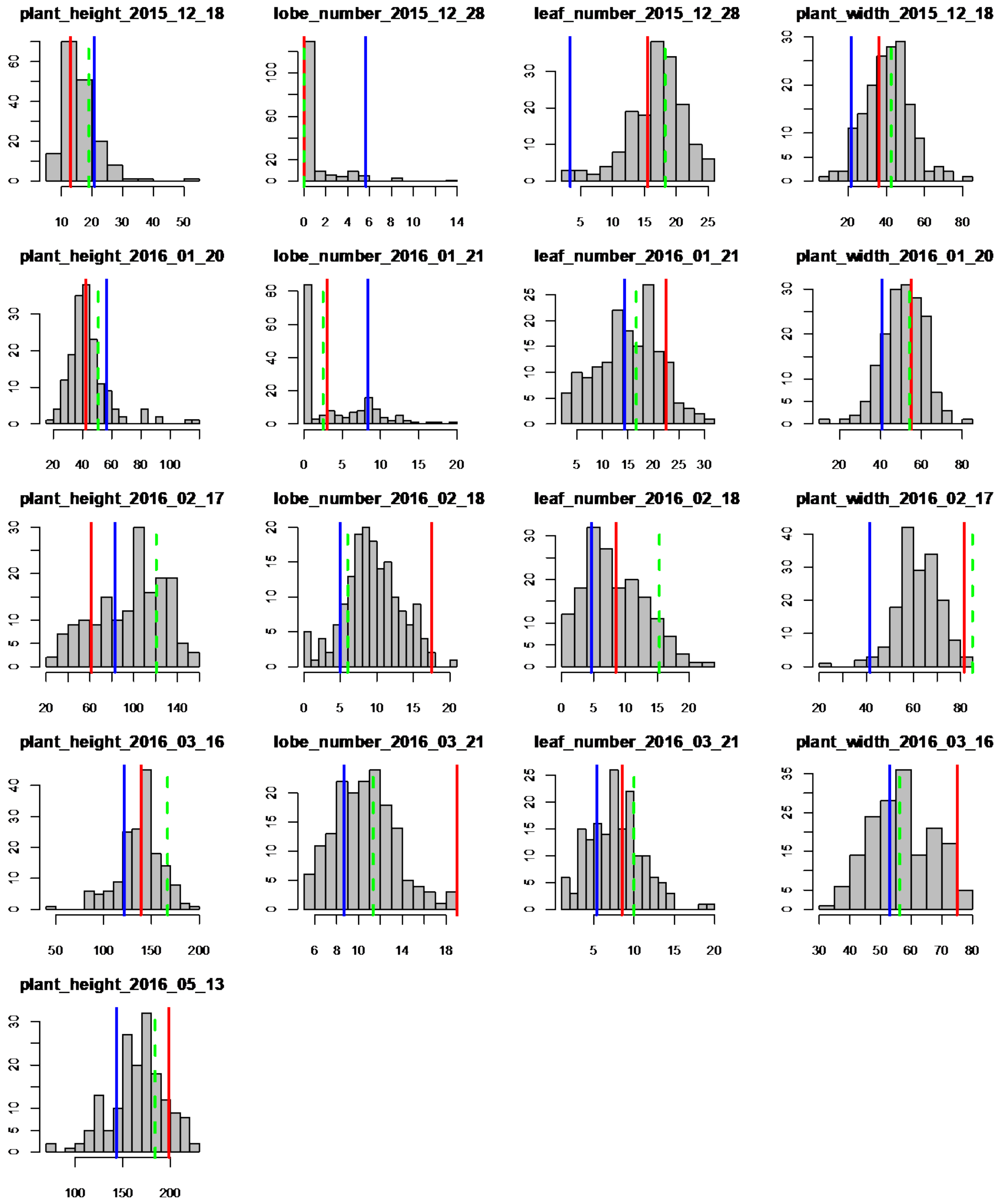

Supplementary Figure 4. Time-series growth data distribution for the F_2 population, parents, and the F_1. Red lines indicate trait values for Da-Ae, blue lines indicate trait values for Da-Ol-1, and green dashed lines indicate trait values for F_1.

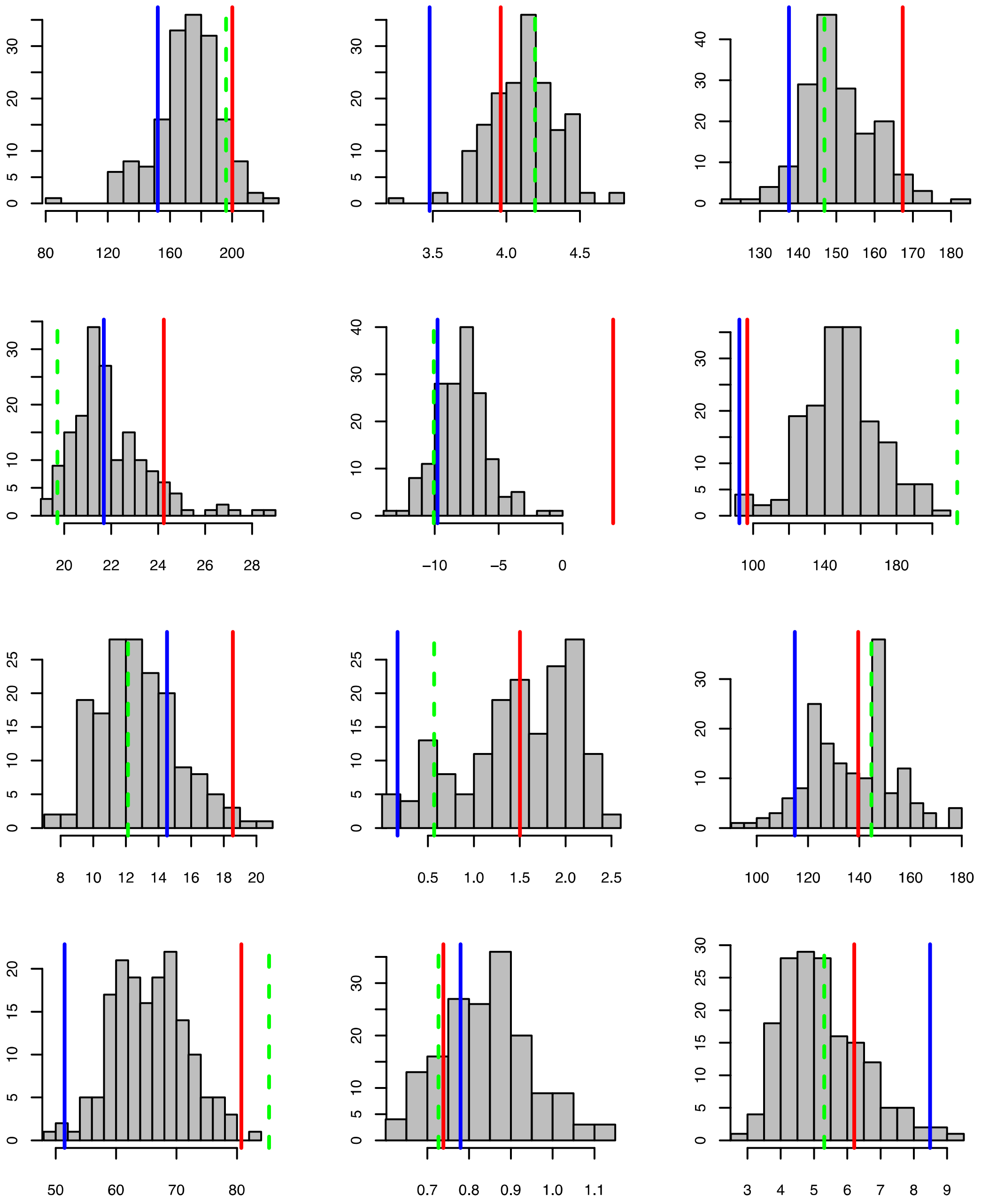

Supplementary Figure 5. Distribution of parameters generated from growth model curve. Red lines indicate trait values for Da-Ae, blue lines indicate trait values for Da-Ol-1, and green dashed lines indicate trait values for F_1.

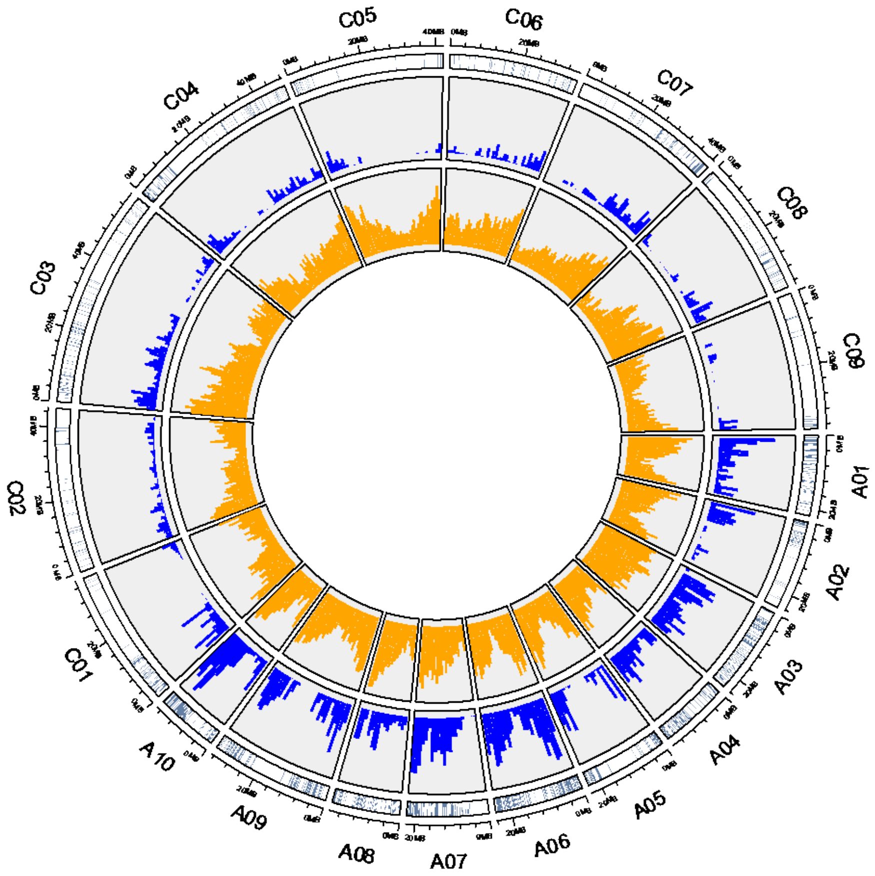

Supplementary Figure 6. Overview of gene density, SNP density, and marker density across the genome. Orange track represents gene density; blue track presents density of SNPs between Da-Ae and Da-Ol-1; the outermost track with blue lines represents density of markers used for genetic map construction.

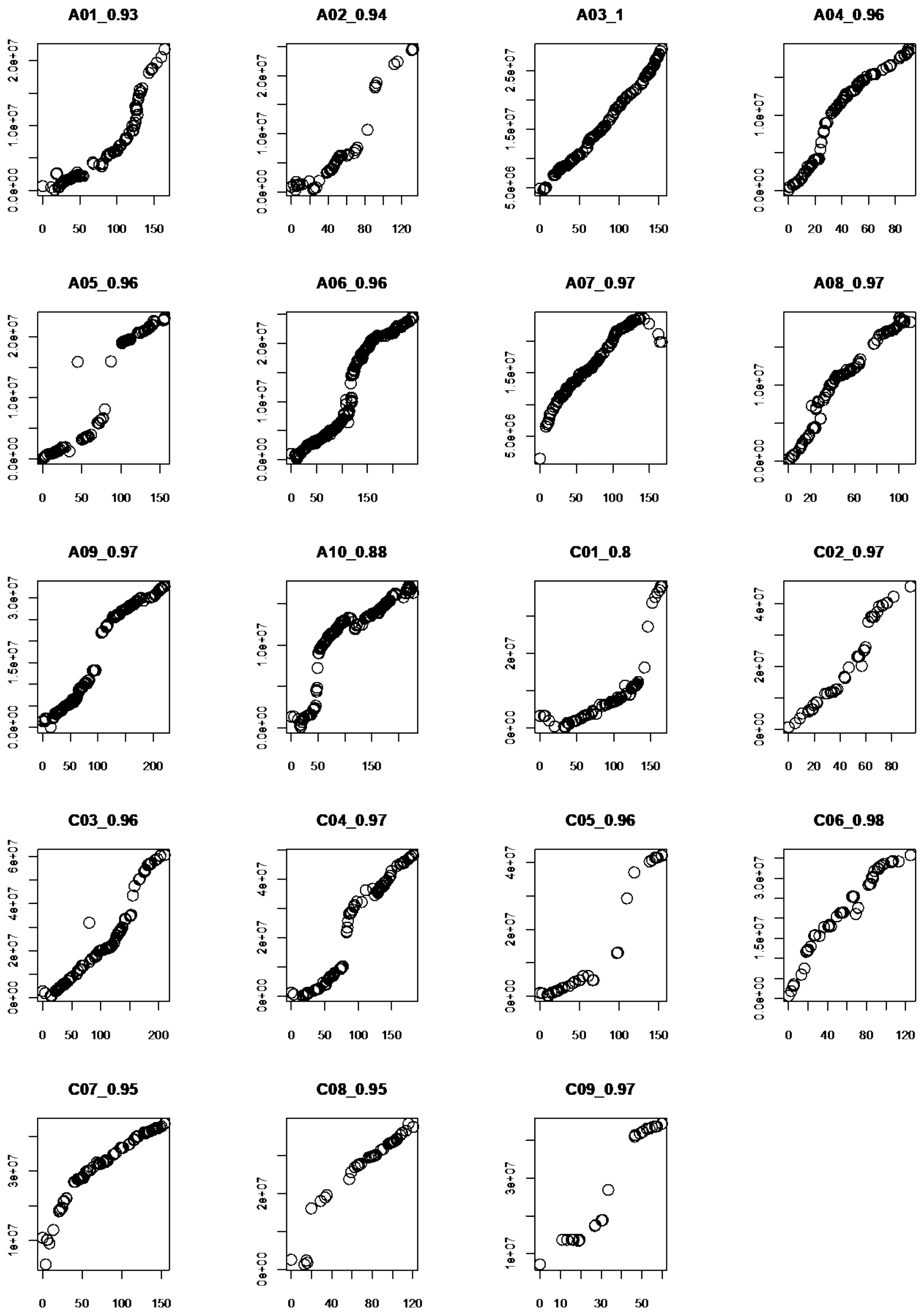
A01_0.93
0.0e+00 1.0e+07 2.0e+07
0 50 100 150
A02_0.94
0.0e+00 1.0e+07 2.0e+07
0 40 80 120
A03_1
5.0e+06 1.5e+07 2.5e+07
0 50 100 150
A04_0.96
0.0e+00 1.0e+07
0 20 40 60 80
A05_0.96
0.0e+00 1.0e+07 2.0e+07
0 50 100 150
A06_0.96
0.0e+00 1.0e+07 2.0e+07
0 50 150
A07_0.97
5.0e+06 1.5e+07
0 50 100 150
A08_0.97
0.0e+00 1.0e+07
0 20 60 100
A09_0.97
0.0e+00 1.5e+07 3.0e+07
0 50 100 200
A10_0.88
0.0e+00 1.0e+07
0 50 150
C01_0.8
0e+00 2e+07
0 50 100 150
C02_0.97
0e+00 2e+07 4e+07
0 20 40 60 80
C03_0.96
0e+00 2e+07 4e+07 6e+07
0 50 100 200
C04_0.97
0e+00 2e+07 4e+07
0 50 100 150
C05_0.96
0e+00 2e+07 4e+07
0 50 100 150
C06_0.98
0.0e+00 1.5e+07 3.0e+07
0 40 80 120
C07_0.95
1e+07 3e+07
0 50 100 150
C08_0.95
0e+00 2e+07
0 40 80 120
C09_0.97
1e+07 3e+07
0 10 30 50

Supplementary Figure 7. Collinearity between the constructed genetic map and the published *Brassica napus* reference genome physical map is quantified using Pearson correlation test (indicated in the title for each subplot), x-axis values represent genetic positions, y-axis values represent physical positions.

Supplementary Figure 8. interval mapping and composite interval mapping results for every phenotypic trait.

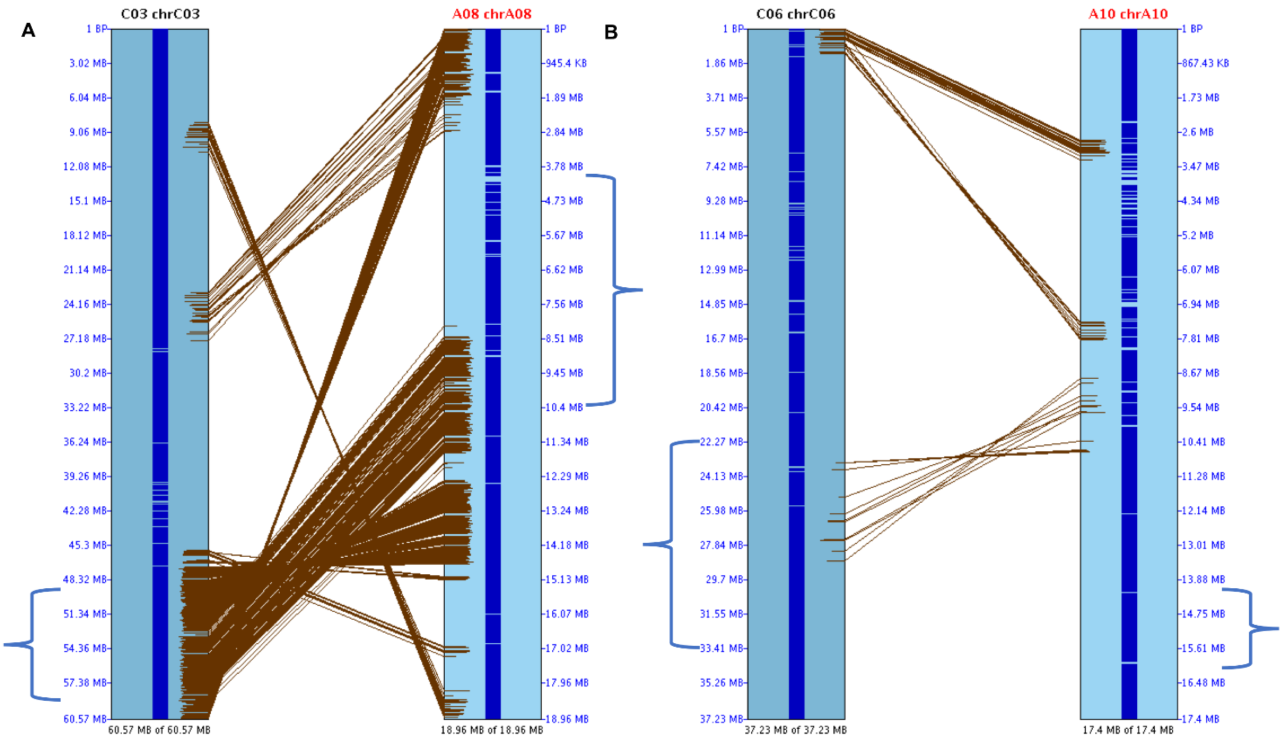

Supplementary Figure 9. Synteny between the two pairs of chromosomes harboring major QTL for fatty acid composition, flowering time, and growth-related traits. (A) synteny between A08 and C03 QTL for different fatty acids; (B) synteny between A10 and C06 QTL for flowering time and growth-related traits. Regions enclosed by brackets are QTL on the corresponding chromosome

Supplementary Tables

Supplementary Table 1 Raw phenotypic data for the 166 F_2s

Supplementary Table 2 RNA-seq sequencing and genome mapping result summary

Supplementary Table 3 Genetic map file for the F_2 population

Supplementary Table 4 List of *cis*-coding candidates for phenotypic traits

Supplementary Table 5 Gene ontology enrichment result for *trans*-eQTL hotspot regulated genes

Supplementary Table 6 List of *cis*-regulators for phenotypic traits

Supplementary Table 7 List of *trans*-eQTL target candidate genes for phenotypic traits

Supplementary Table 8 List of candidate genes for phenotypic traits discussed in the text
