# Supplementary material for: Integrated QTL and eQTL Mapping Provides Insights and Candidate Genes for Fatty Acid Composition, Flowering Time, and Growth Traits in a F2 Population of a Novel Synthetic Allopolyploid Brassica napus
Source: Front Plant Sci. 2018 Nov 13;9:1632. doi: 10.3389/fpls.2018.01632 (PMC6243938; doi:10.3389/fpls.2018.01632)
Supplement: Supplementary file 10 [file Data_Sheet_1.PDF]

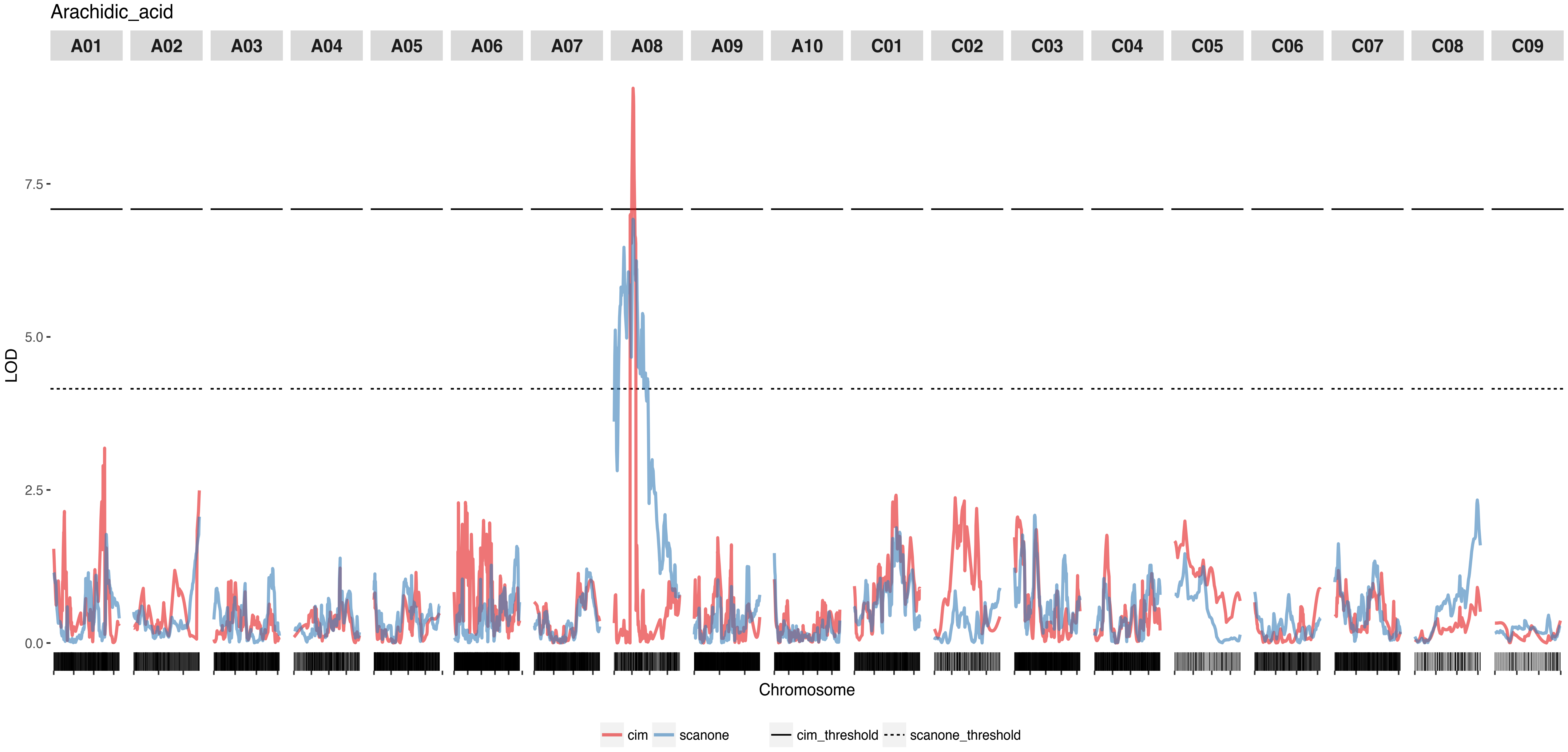

Arachidic_acid
A01
A02
A03
A04
A05
A06
A07
A08
A09
A10
C01
C02
C03
C04
C05
C06
C07
C08
C09
LOD
7.5
5.0
2.5
0.0
Chromosome
cim
scanone
cim_threshold
scanone_threshold

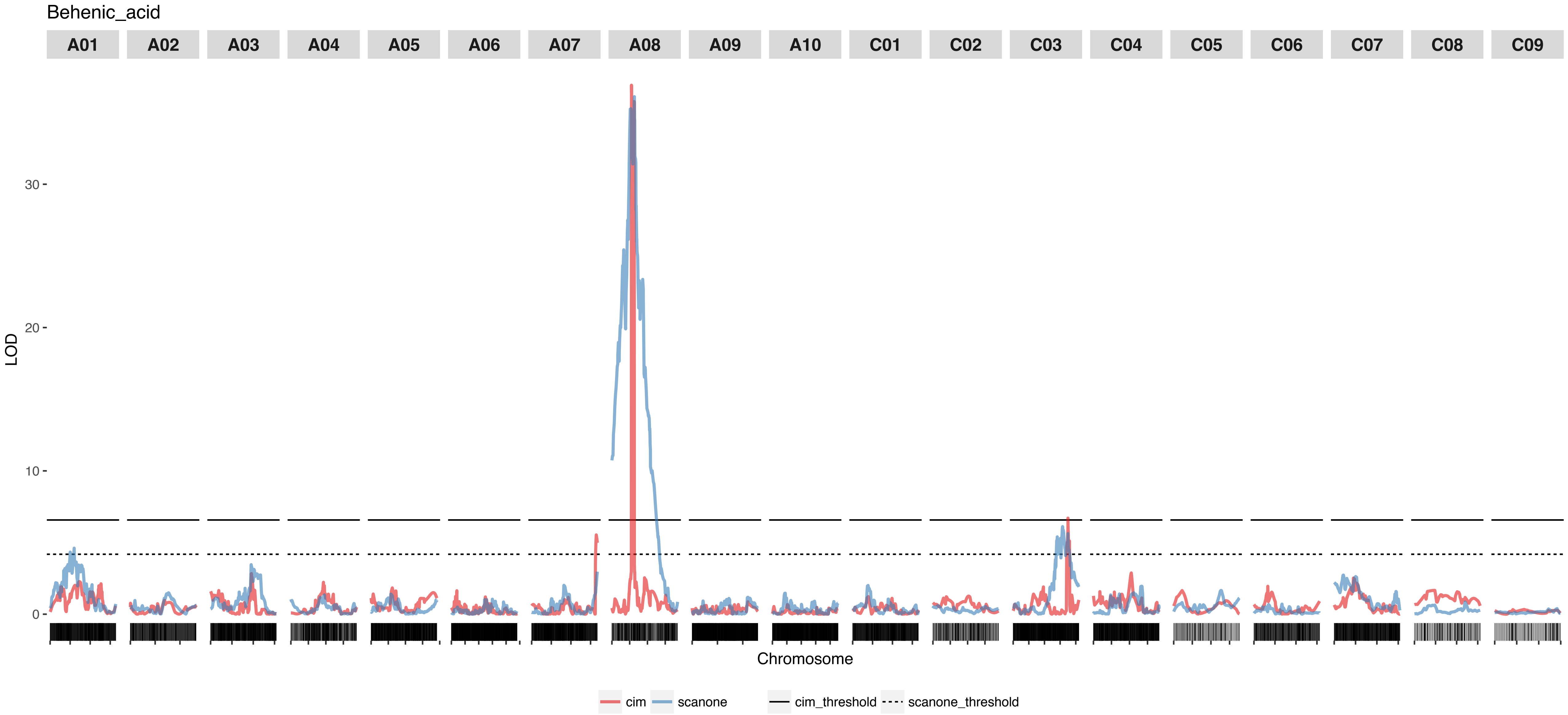
Behenic_acid
A01
A02
A03
A04
A05
A06
A07
A08
A09
A10
C01
C02
C03
C04
C05
C06
C07
C08
C09
LOD
30
20
10
0
Chromosome
cim
scanone
cim_threshold
scanone_threshold

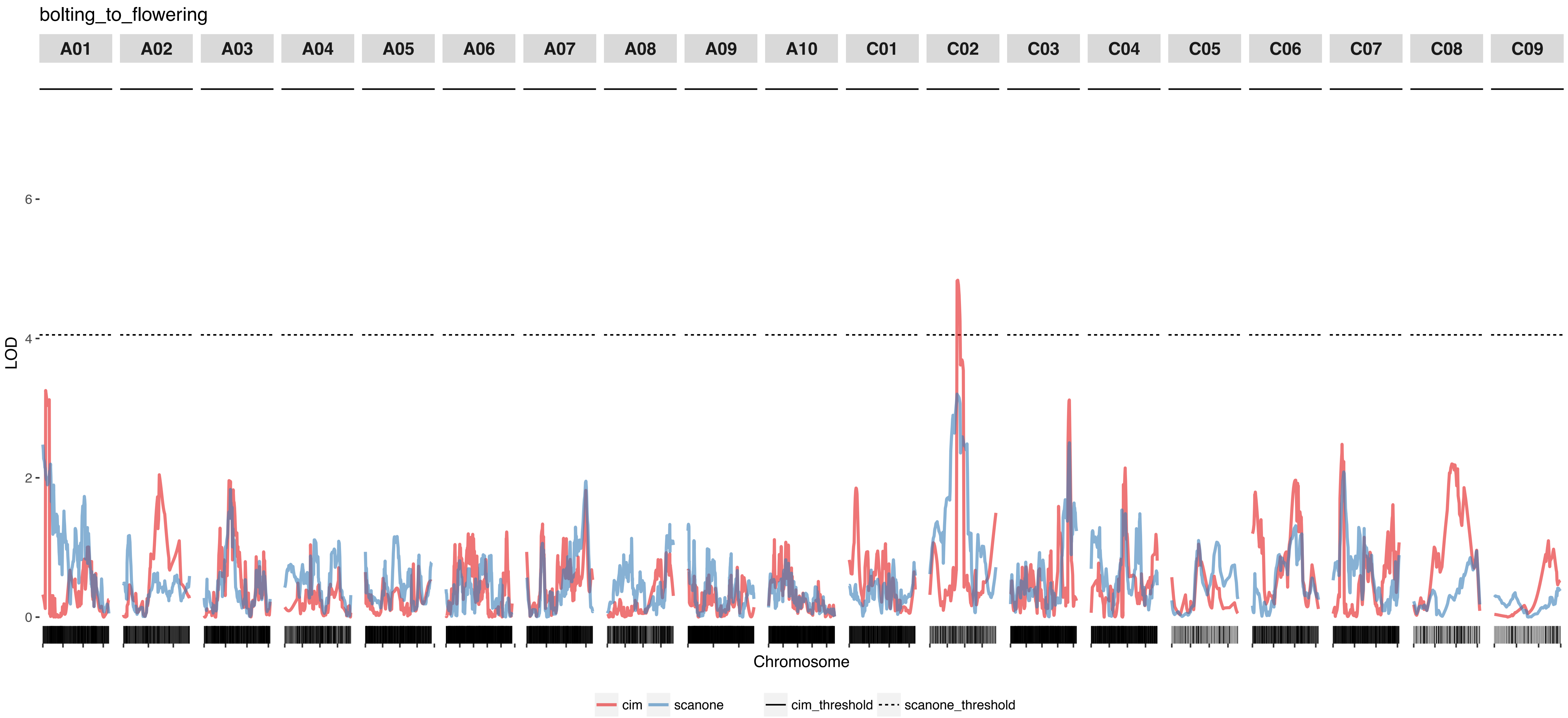
bolting_to_flowering
A01
A02
A03
A04
A05
A06
A07
A08
A09
A10
C01
C02
C03
C04
C05
C06
C07
C08
C09
LOD
6
4
2
0
Chromosome
cim
scanone
cim_threshold
scanone_threshold

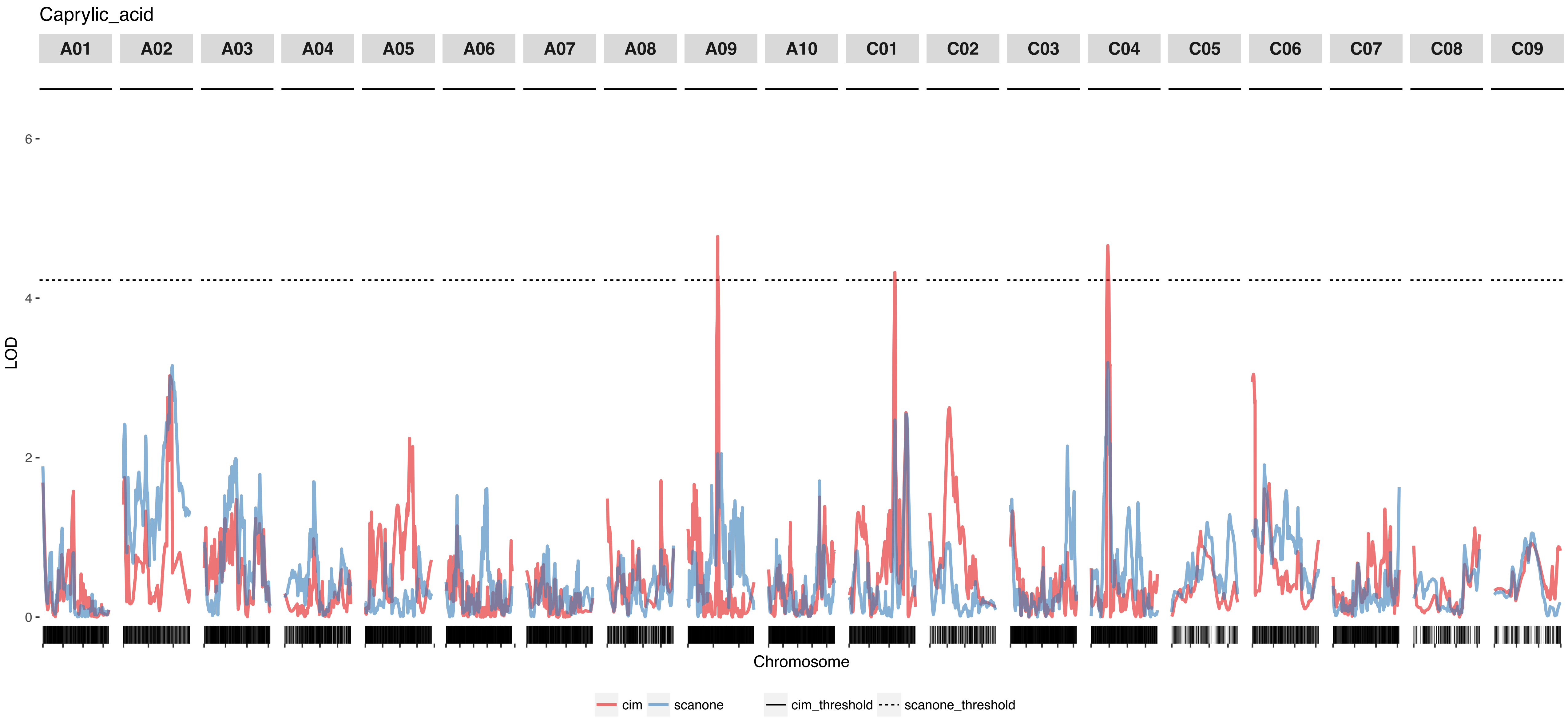
Caprylic_acid
A01
A02
A03
A04
A05
A06
A07
A08
A09
A10
C01
C02
C03
C04
C05
C06
C07
C08
C09
LOD
6
4
2
0
Chromosome
cim
scanone
cim_threshold
scanone_threshold

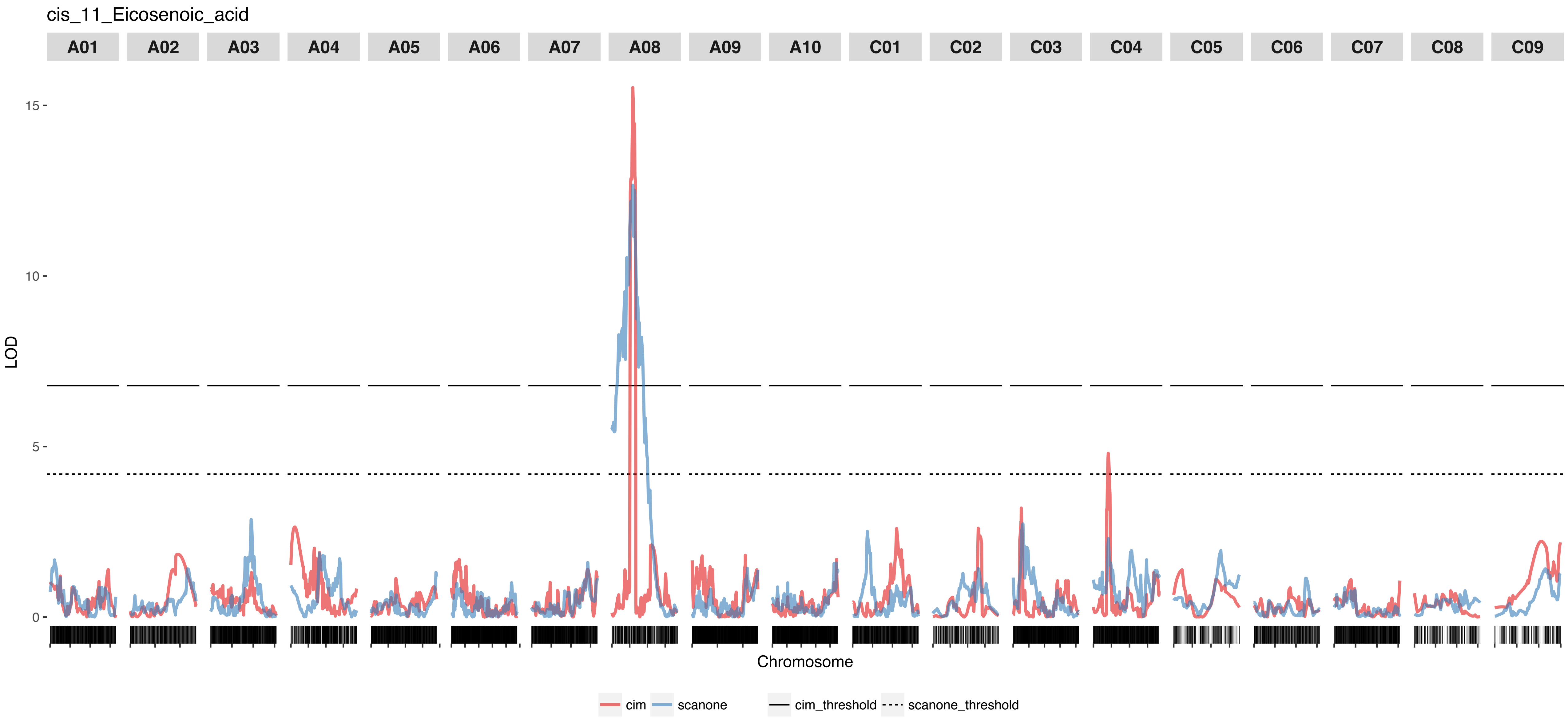
cis_11_Eicosenoic_acid
A01
A02
A03
A04
A05
A06
A07
A08
A09
A10
C01
C02
C03
C04
C05
C06
C07
C08
C09
15
10
5
0
LOD
Chromosome
cim
scanone
cim_threshold
scanone_threshold

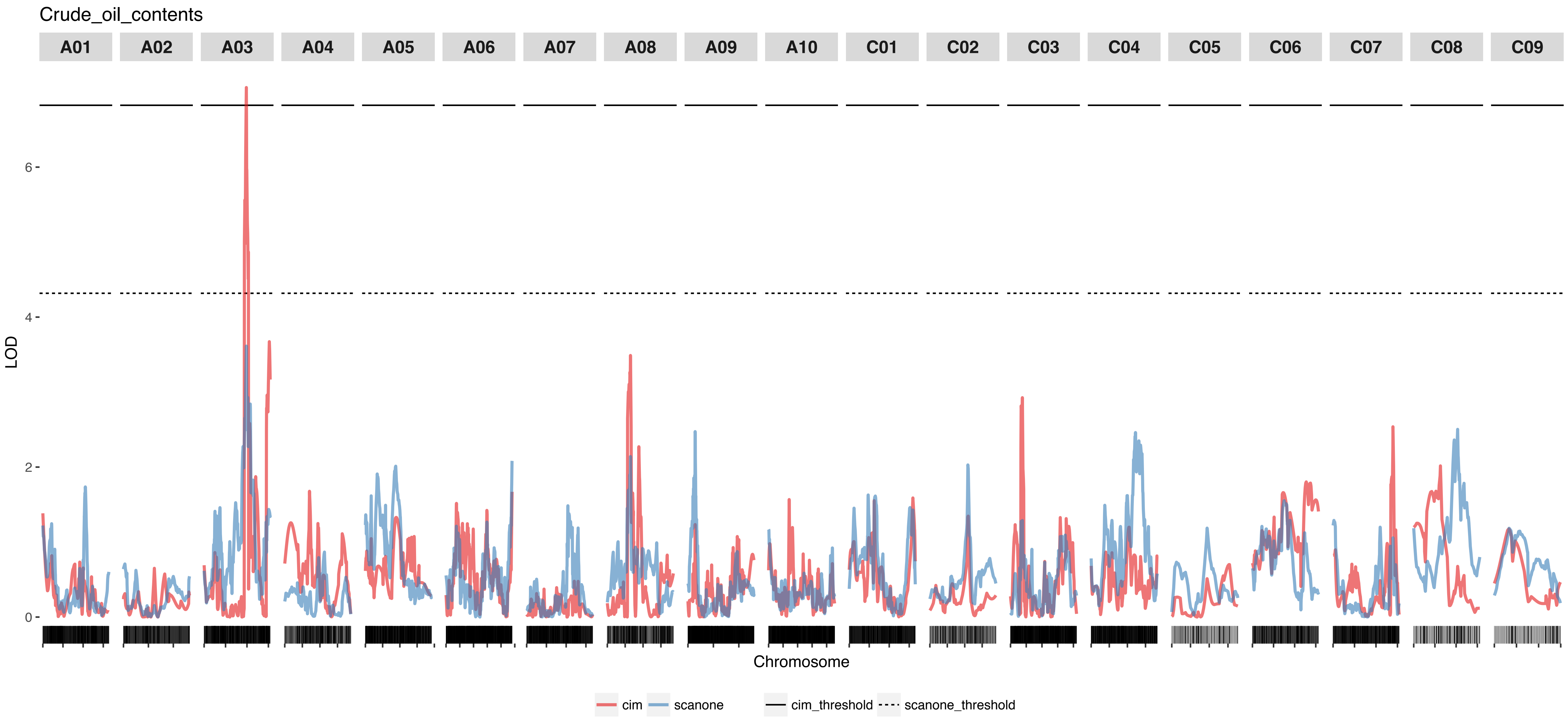

Crude_oil_contents
A01
A02
A03
A04
A05
A06
A07
A08
A09
A10
C01
C02
C03
C04
C05
C06
C07
C08
C09
LOD
6
4
2
0
Chromosome
cim
scanone
cim_threshold
scanone_threshold

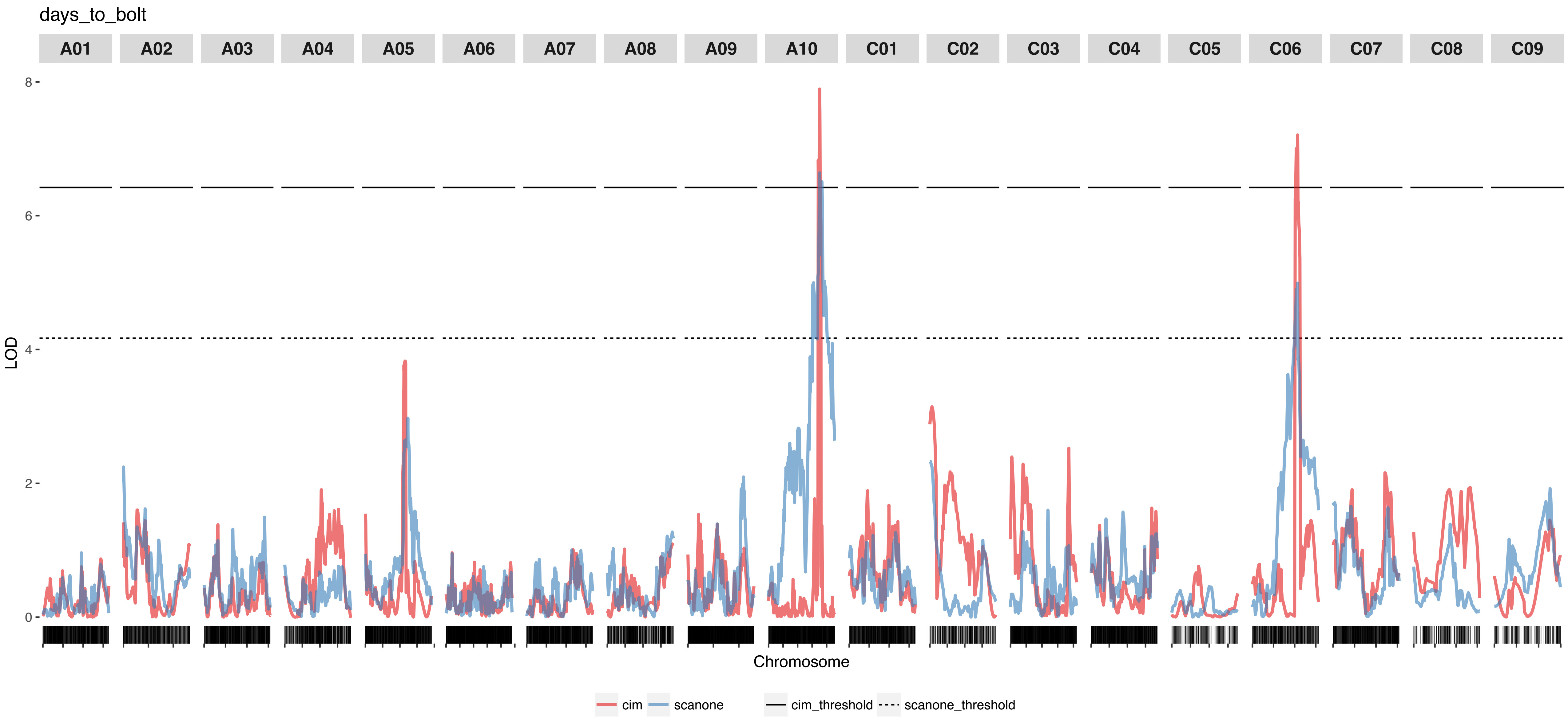
days_to_bolt
A01
A02
A03
A04
A05
A06
A07
A08
A09
A10
C01
C02
C03
C04
C05
C06
C07
C08
C09
LOD
0
2
4
6
8
Chromosome
cim
scanone
cim_threshold
scanone_threshold

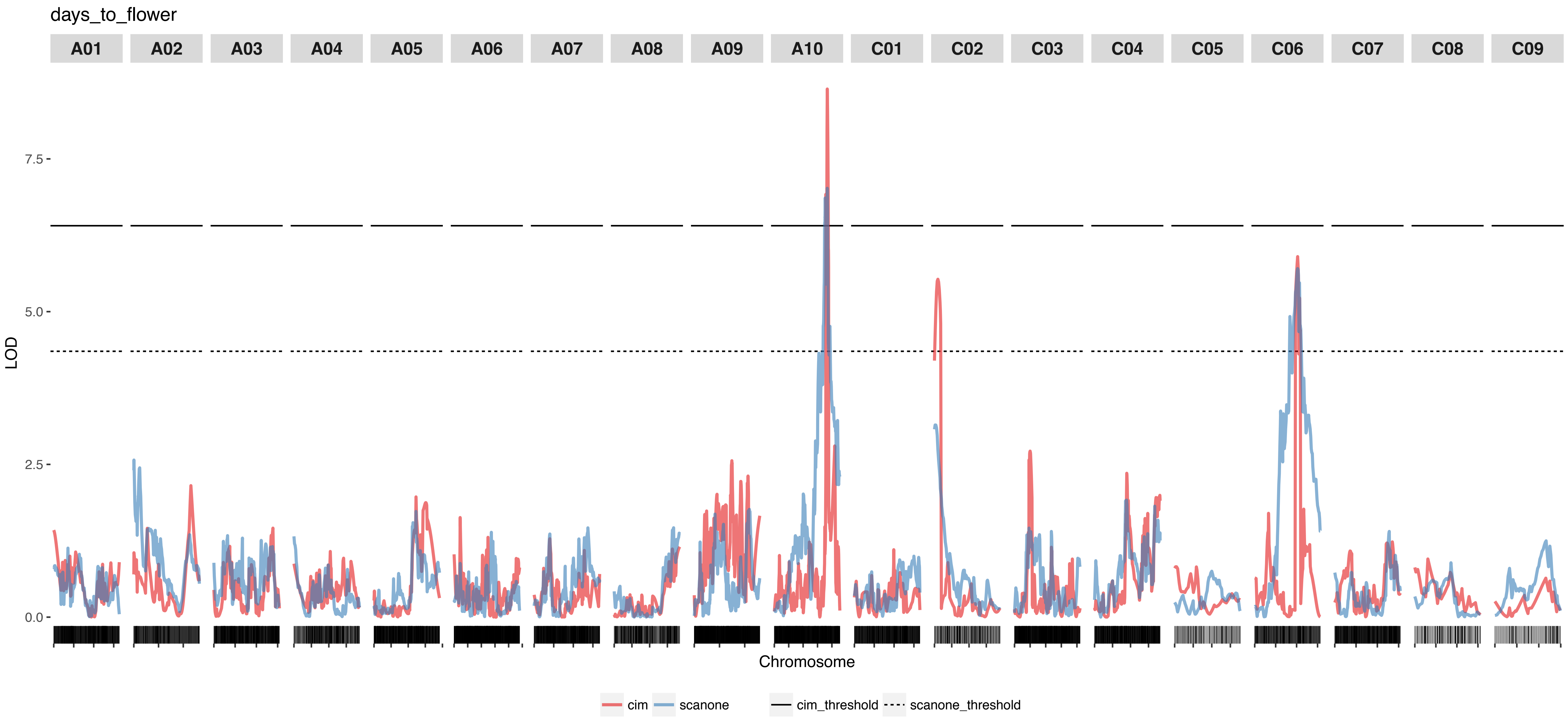
days_to_flower
A01
A02
A03
A04
A05
A06
A07
A08
A09
A10
C01
C02
C03
C04
C05
C06
C07
C08
C09
7.5
5.0
2.5
0.0
LOD
Chromosome
cim
scanone
cim_threshold
scanone_threshold

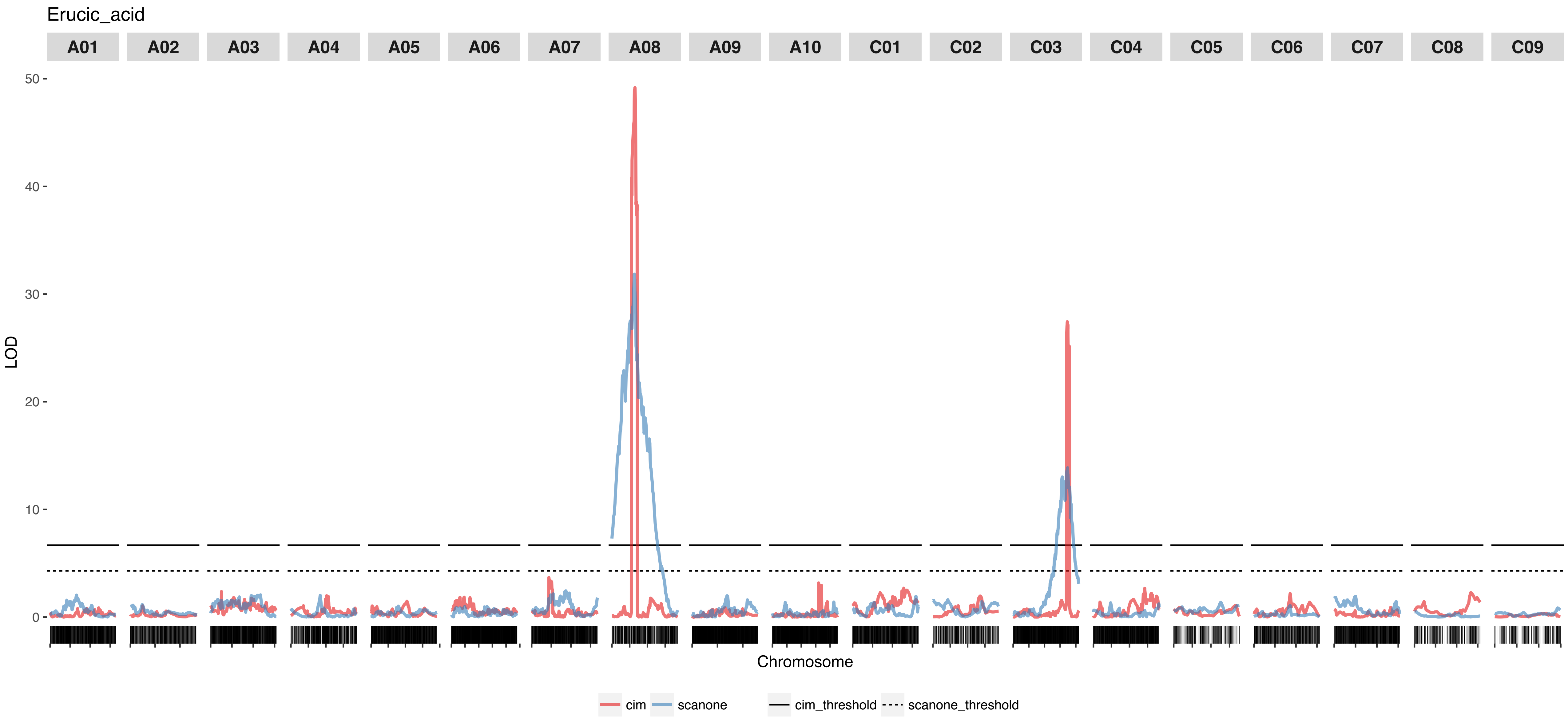

Erucic_acid
A01
A02
A03
A04
A05
A06
A07
A08
A09
A10
C01
C02
C03
C04
C05
C06
C07
C08
C09
50
40
30
20
10
0
LOD
Chromosome
cim
scanone
cim_threshold
scanone_threshold

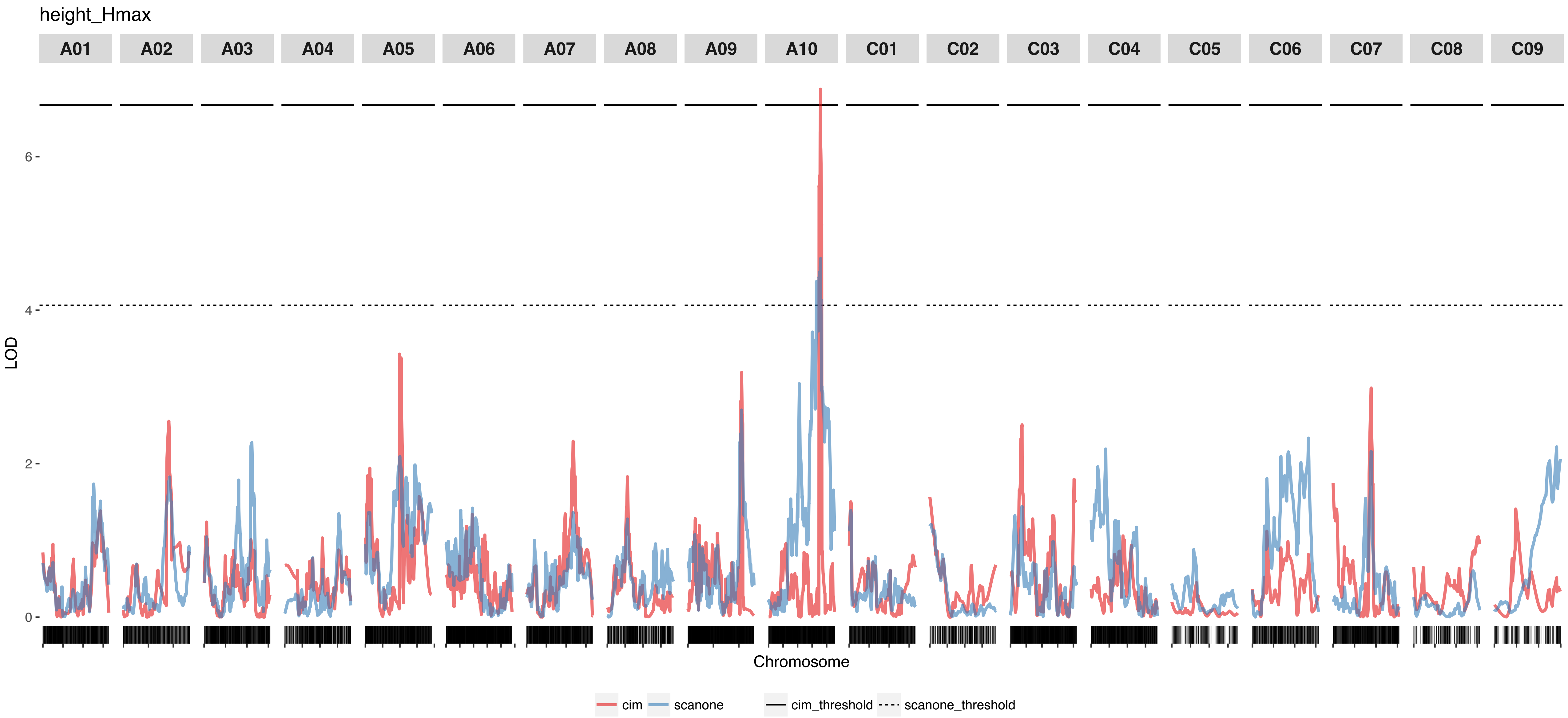

height_Hmax
A01
A02
A03
A04
A05
A06
A07
A08
A09
A10
C01
C02
C03
C04
C05
C06
C07
C08
C09
LOD
0
2
4
6
Chromosome
cim
scanone
cim_threshold
scanone_threshold

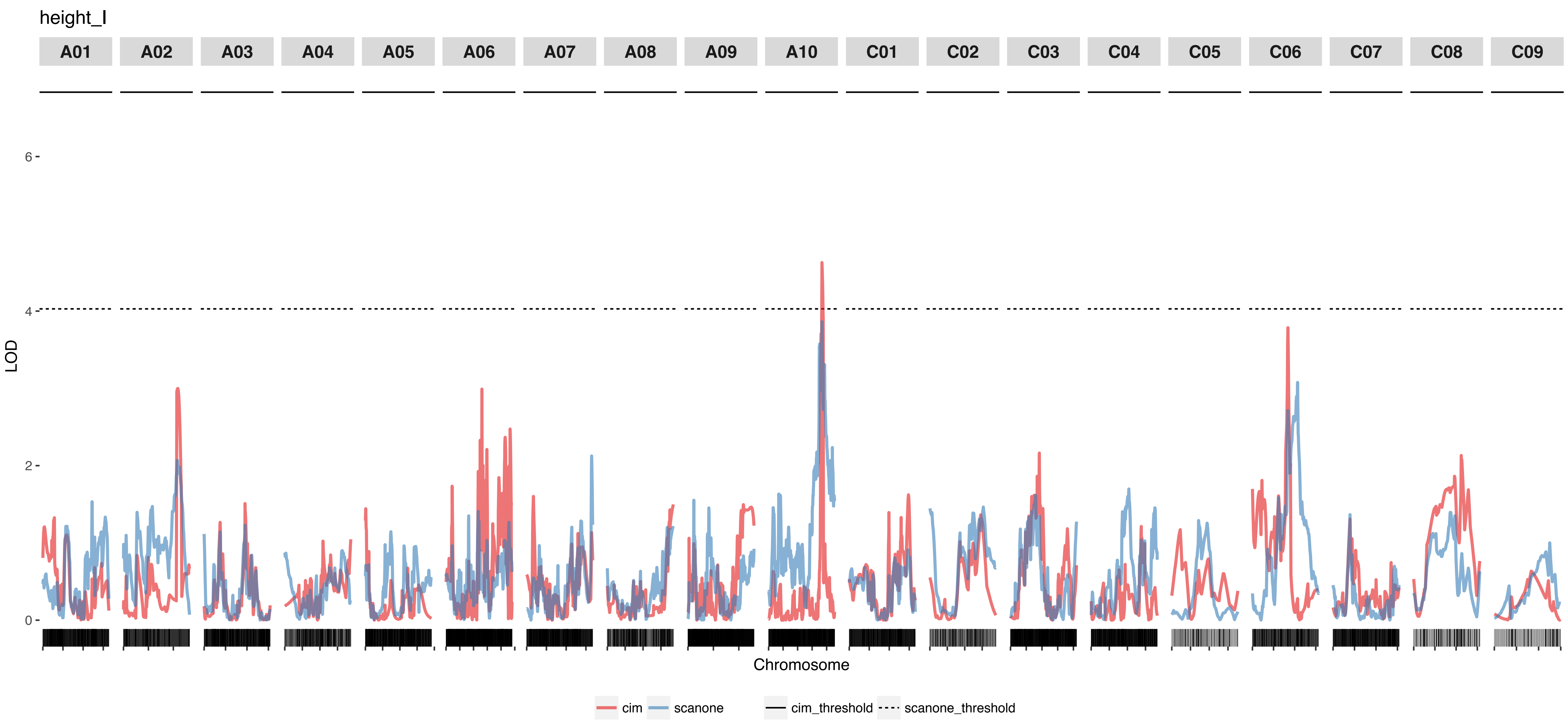
height_I
A01
A02
A03
A04
A05
A06
A07
A08
A09
A10
C01
C02
C03
C04
C05
C06
C07
C08
C09
LOD
6
4
2
0
Chromosome
cim
scanone
cim_threshold
scanone_threshold

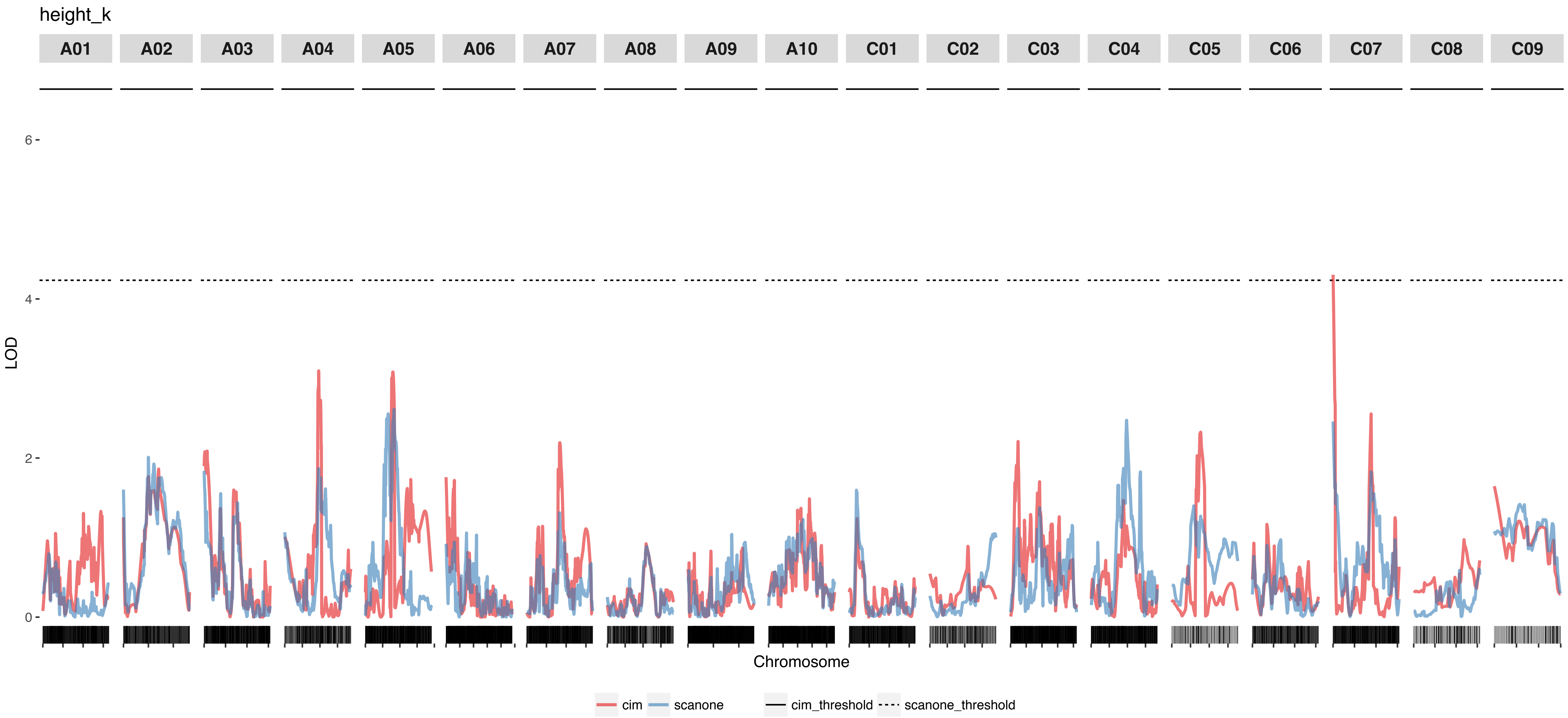
height_k
A01
A02
A03
A04
A05
A06
A07
A08
A09
A10
C01
C02
C03
C04
C05
C06
C07
C08
C09
LOD
6
4
2
0
Chromosome
cim
scanone
cim_threshold
scanone_threshold

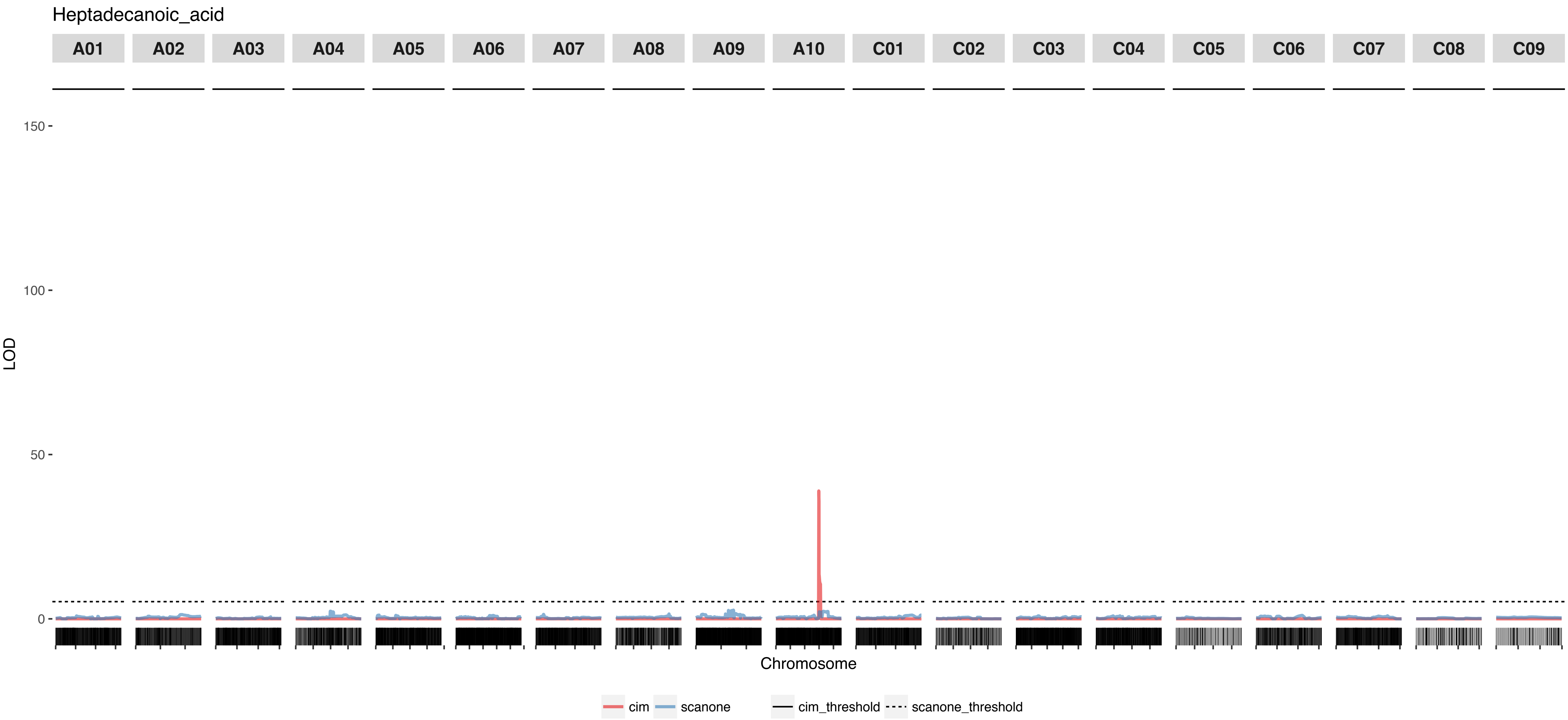
Heptadecanoic_acid
A01
A02
A03
A04
A05
A06
A07
A08
A09
A10
C01
C02
C03
C04
C05
C06
C07
C08
C09
150
100
50
0
LOD
Chromosome
cim
scanone
cim_threshold
scanone_threshold

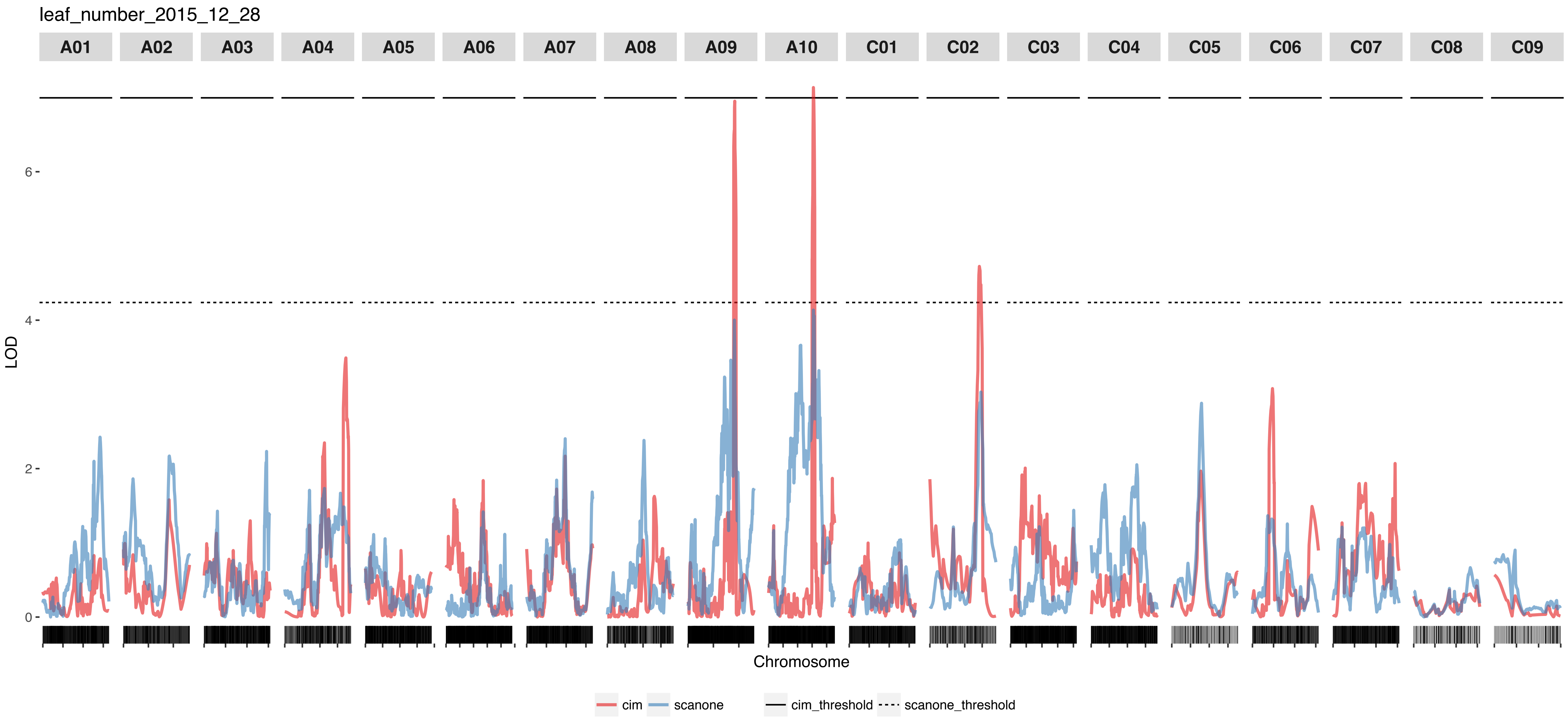

leaf_number_2015_12_28
A01
A02
A03
A04
A05
A06
A07
A08
A09
A10
C01
C02
C03
C04
C05
C06
C07
C08
C09
LOD
6
4
2
0
Chromosome
cim
scanone
cim_threshold
scanone_threshold

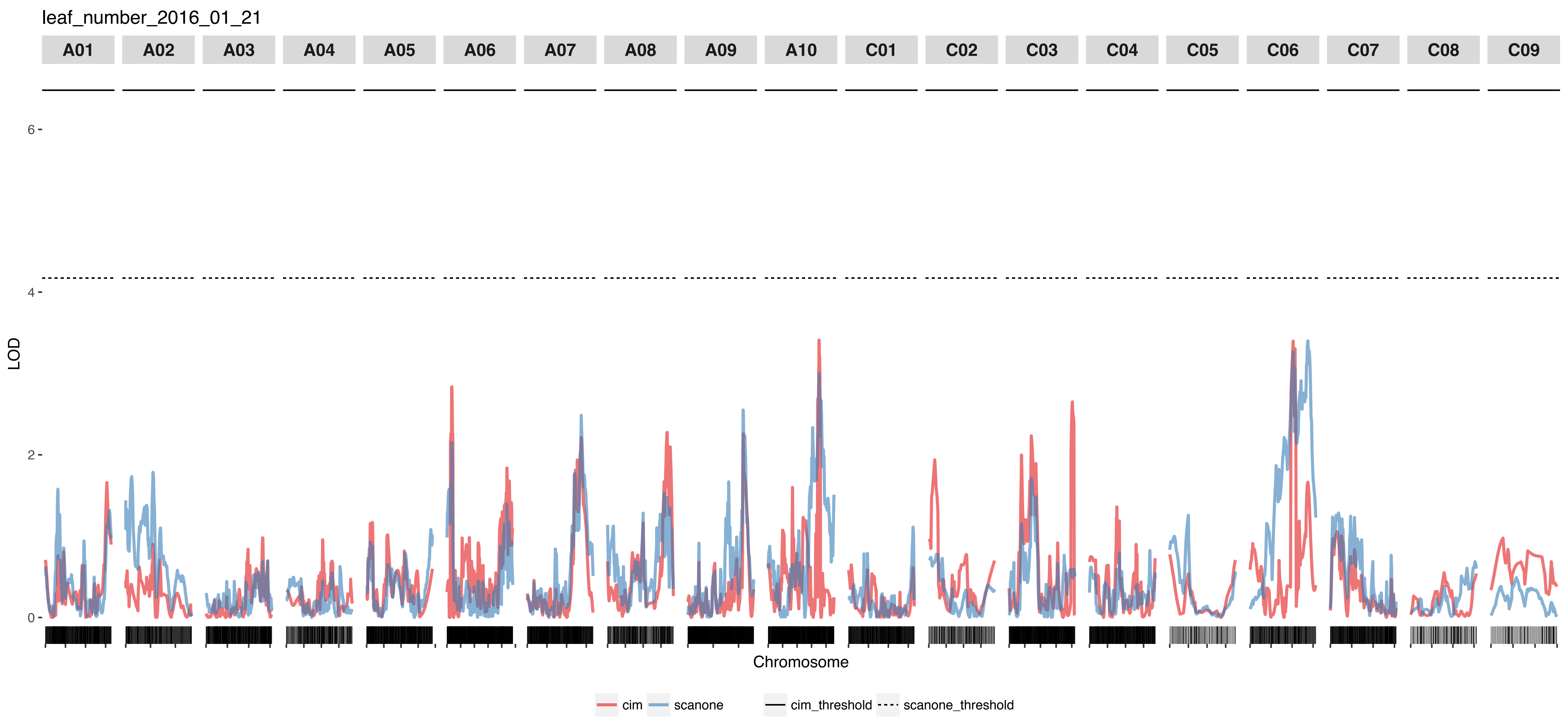
leaf_number_2016_01_21
A01
A02
A03
A04
A05
A06
A07
A08
A09
A10
C01
C02
C03
C04
C05
C06
C07
C08
C09
LOD
6
4
2
0
Chromosome
cim
scanone
cim_threshold
scanone_threshold

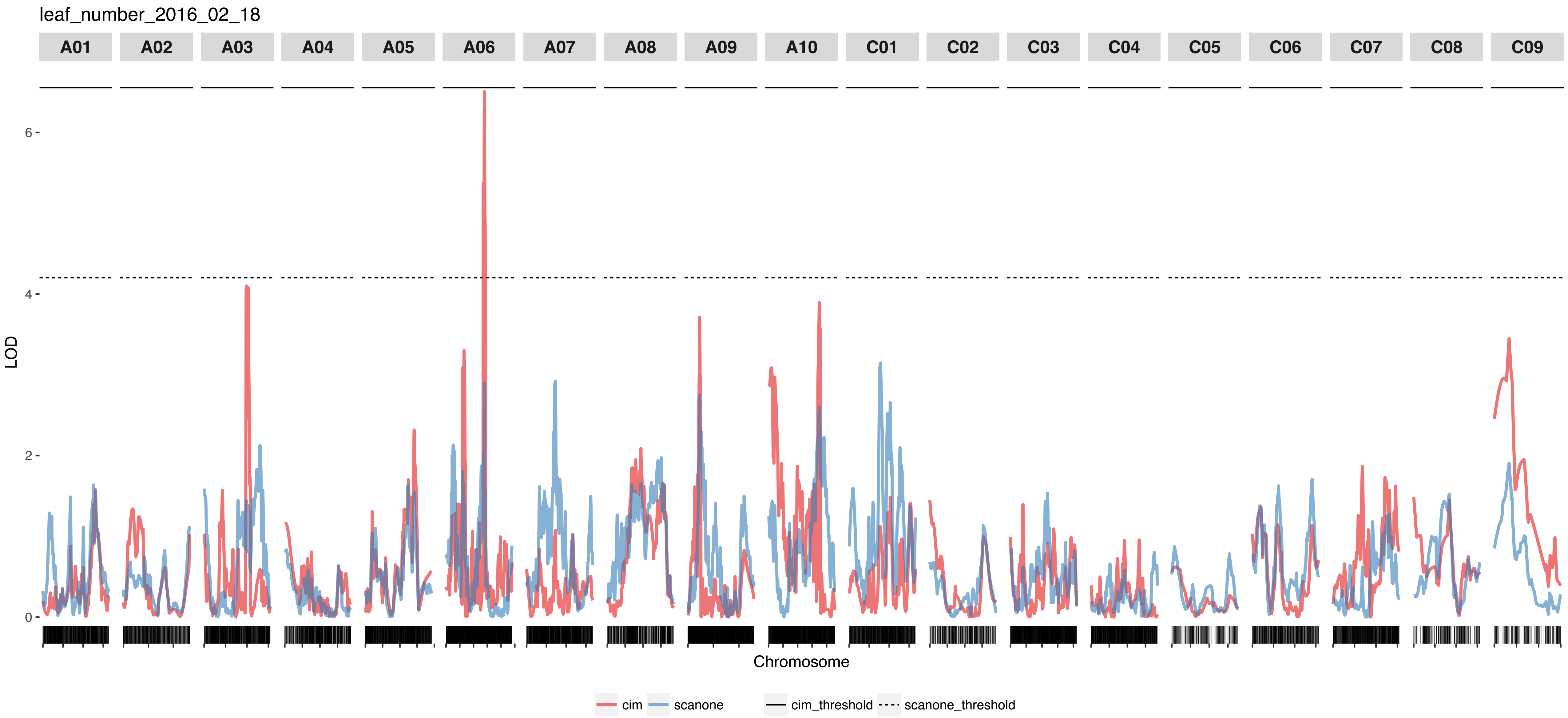

leaf_number_2016_02_18
A01
A02
A03
A04
A05
A06
A07
A08
A09
A10
C01
C02
C03
C04
C05
C06
C07
C08
C09
LOD
6
4
2
0
Chromosome
cim
scanone
cim_threshold
scanone_threshold

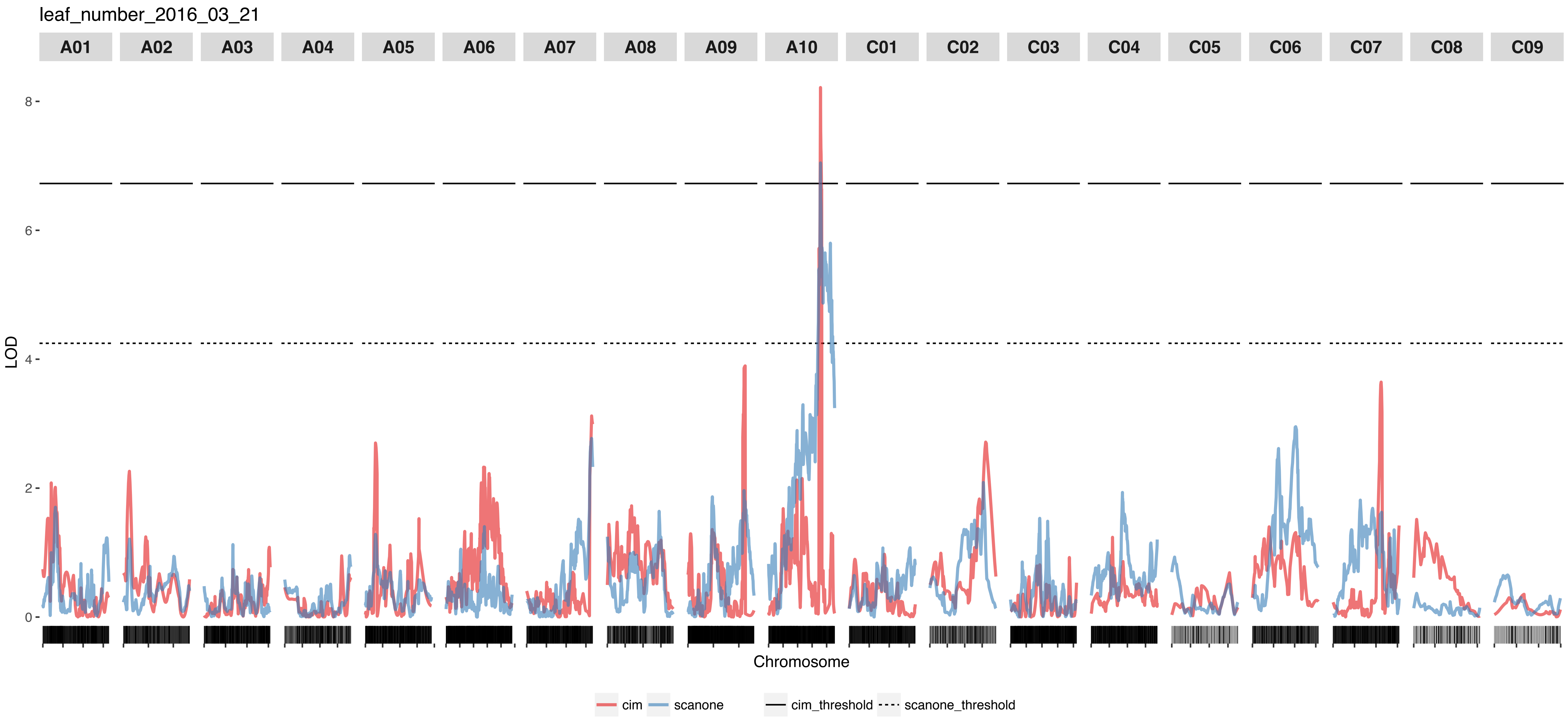

leaf_number_2016_03_21
A01
A02
A03
A04
A05
A06
A07
A08
A09
A10
C01
C02
C03
C04
C05
C06
C07
C08
C09
LOD
8
6
4
2
0
Chromosome
cim
scanone
cim_threshold
scanone_threshold

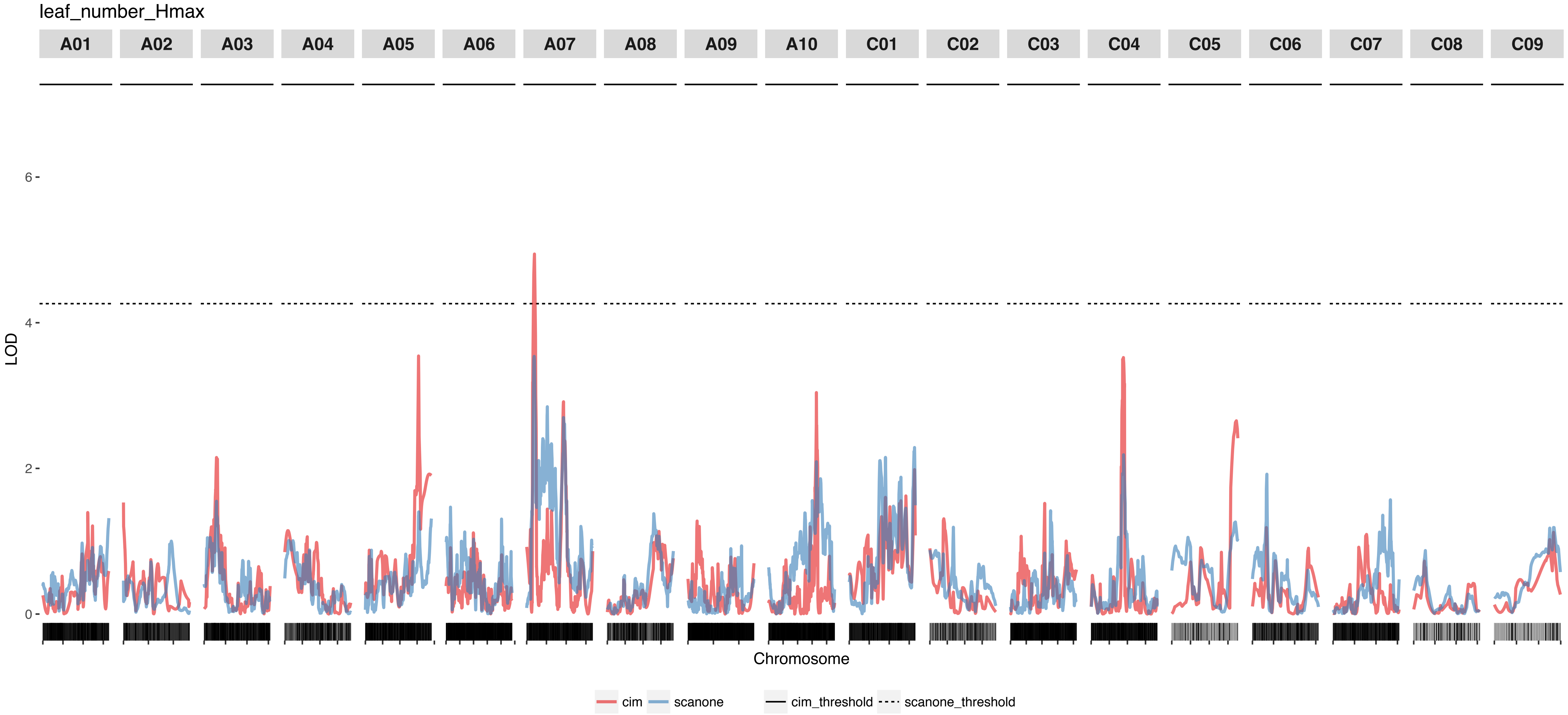
leaf_number_Hmax
A01
A02
A03
A04
A05
A06
A07
A08
A09
A10
C01
C02
C03
C04
C05
C06
C07
C08
C09
LOD
6
4
2
0
Chromosome
cim
scanone
cim_threshold
scanone_threshold

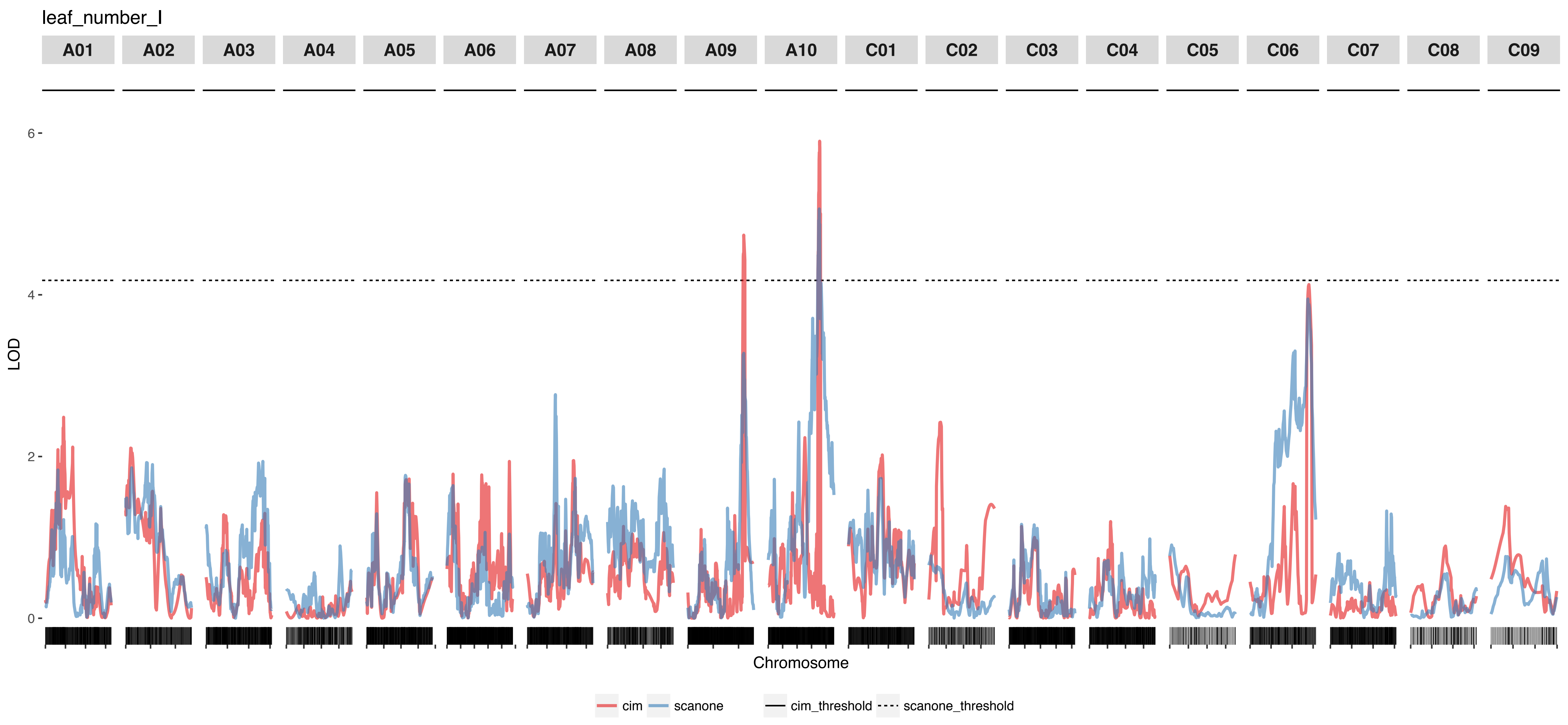
leaf_number_I
A01
A02
A03
A04
A05
A06
A07
A08
A09
A10
C01
C02
C03
C04
C05
C06
C07
C08
C09
LOD
6
4
2
0
Chromosome
cim
scanone
cim_threshold
scanone_threshold

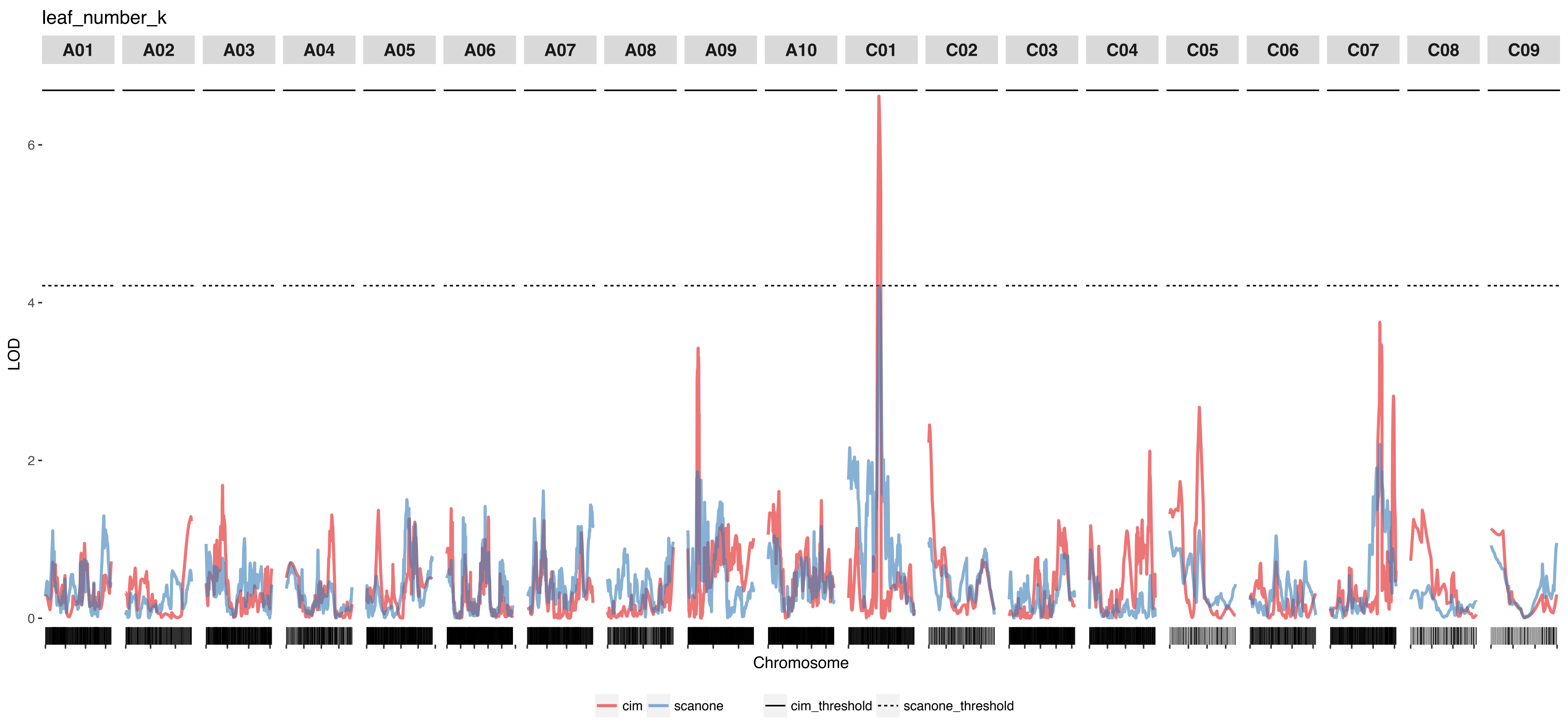
leaf_number_k
A01
A02
A03
A04
A05
A06
A07
A08
A09
A10
C01
C02
C03
C04
C05
C06
C07
C08
C09
LOD
6
4
2
0
Chromosome
cim
scanone
cim_threshold
scanone_threshold

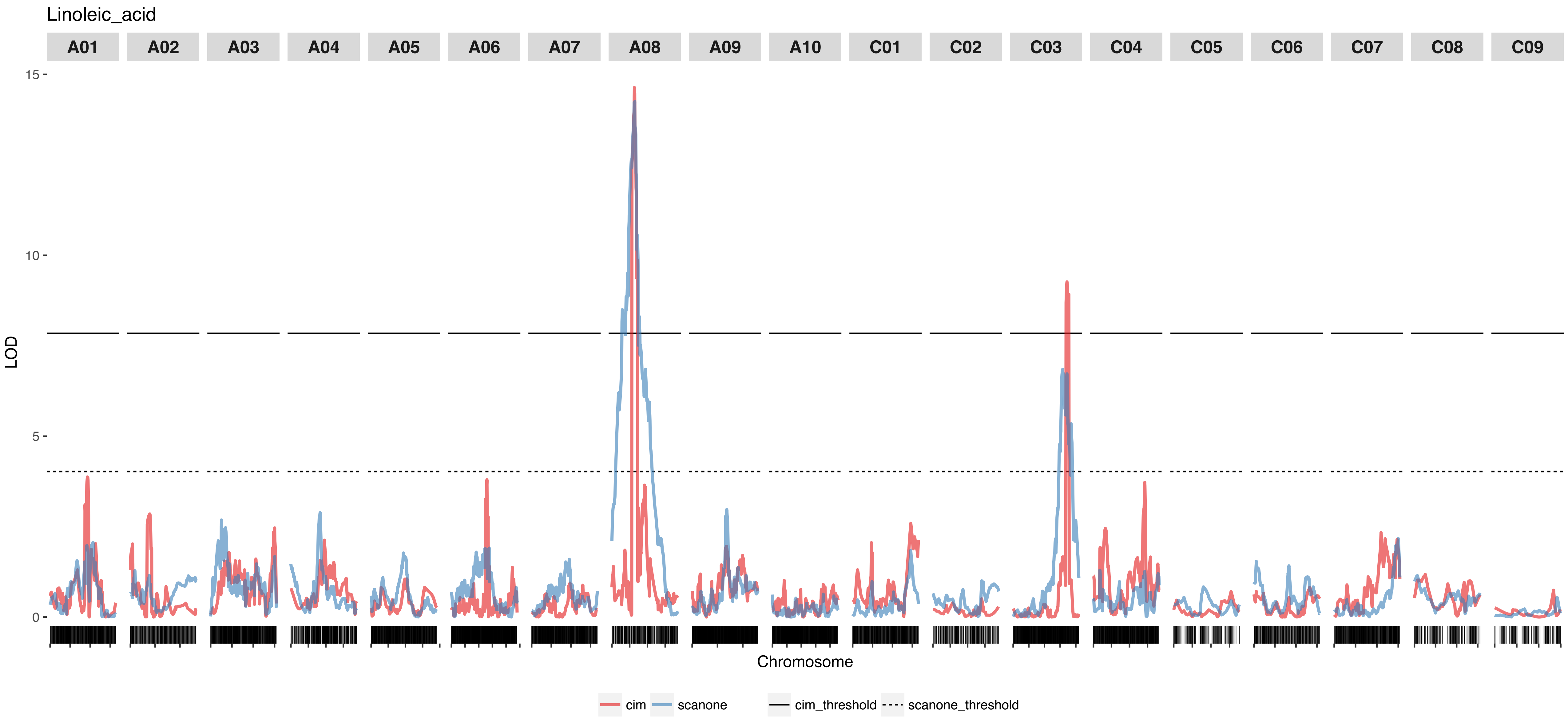

Linoleic_acid
A01
A02
A03
A04
A05
A06
A07
A08
A09
A10
C01
C02
C03
C04
C05
C06
C07
C08
C09
15
10
5
0
LOD
Chromosome
cim
scanone
cim_threshold
scanone_threshold

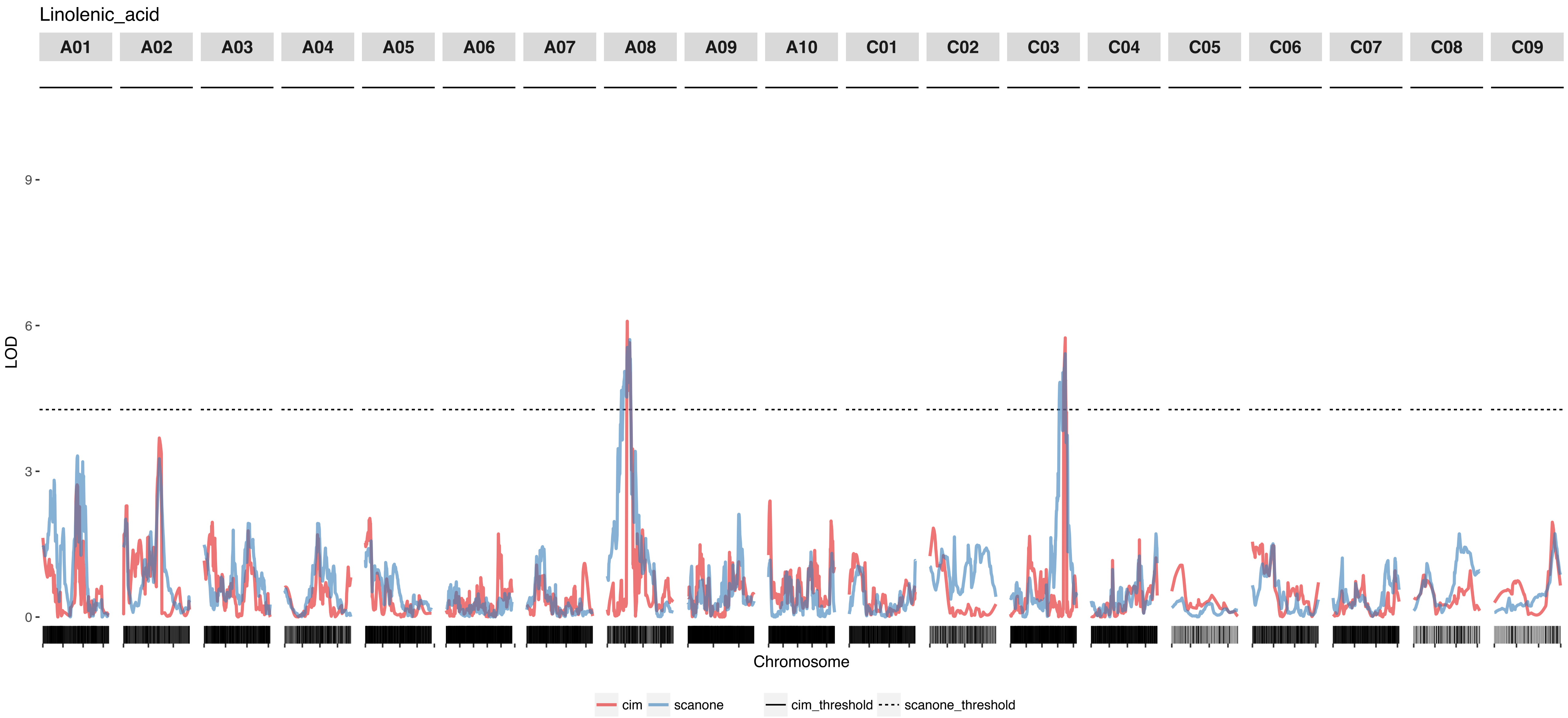

Linolenic_acid
A01
A02
A03
A04
A05
A06
A07
A08
A09
A10
C01
C02
C03
C04
C05
C06
C07
C08
C09
LOD
9
6
3
0
Chromosome
cim
scanone
cim_threshold
scanone_threshold

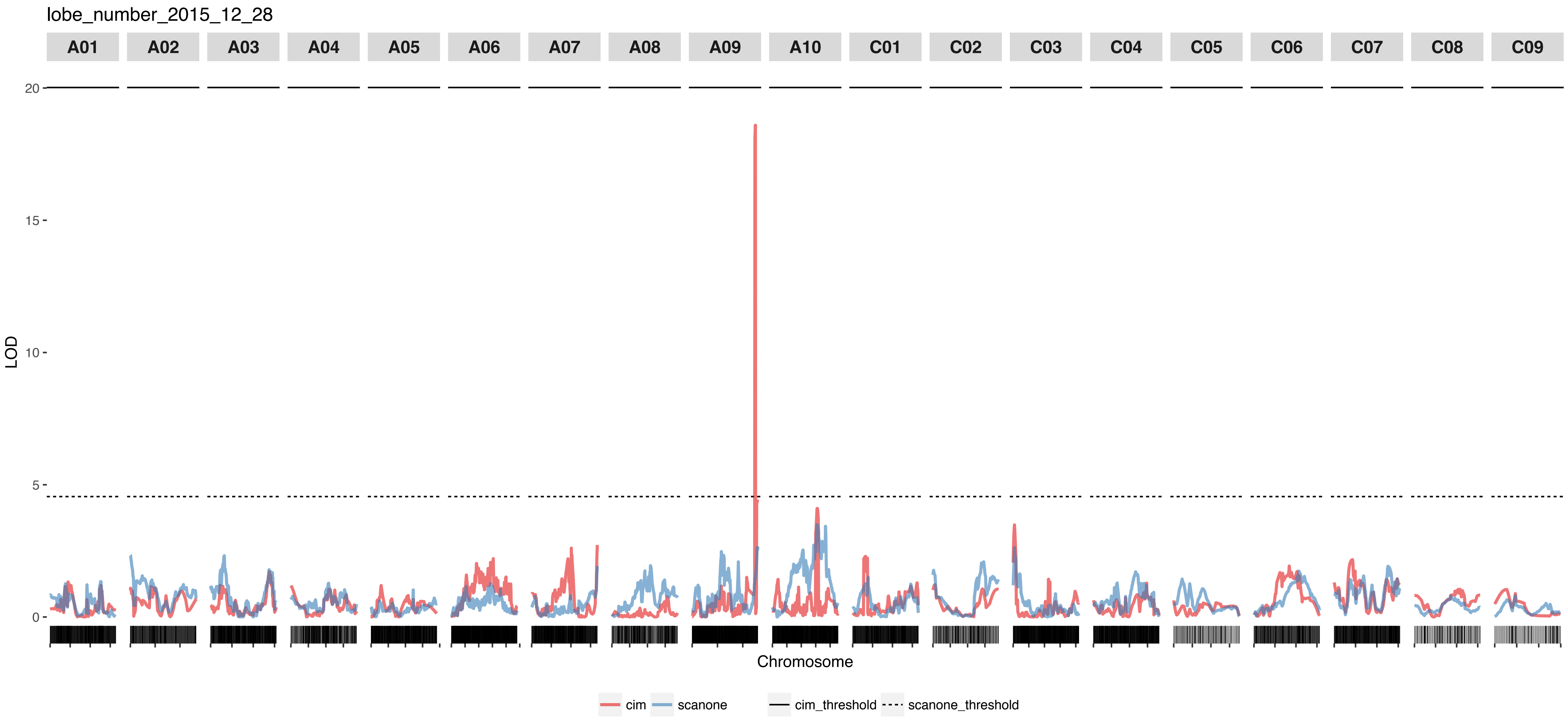
lobe_number_2015_12_28
A01
A02
A03
A04
A05
A06
A07
A08
A09
A10
C01
C02
C03
C04
C05
C06
C07
C08
C09
20
15
10
5
0
LOD
Chromosome
cim
scanone
cim_threshold
scanone_threshold

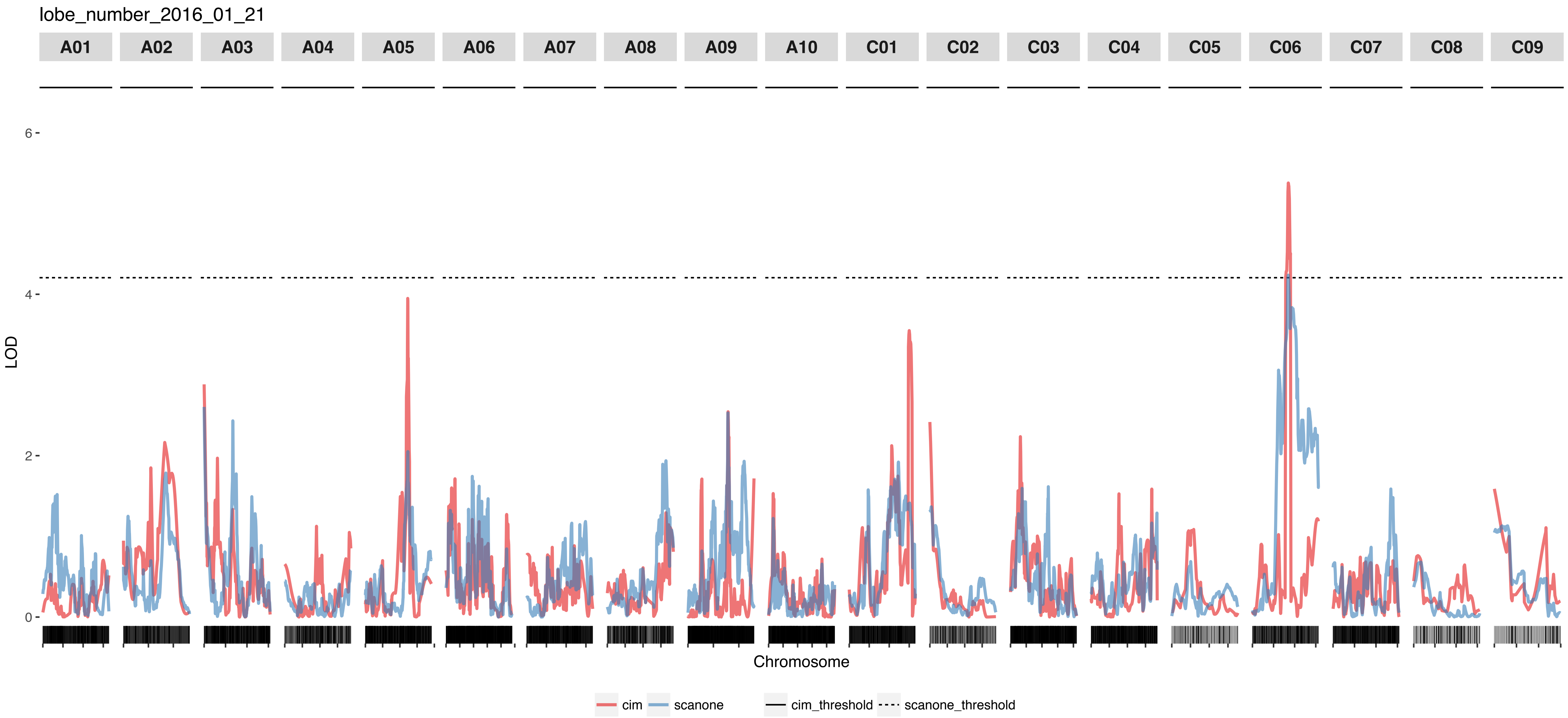
lobe_number_2016_01_21
A01
A02
A03
A04
A05
A06
A07
A08
A09
A10
C01
C02
C03
C04
C05
C06
C07
C08
C09
LOD
6
4
2
0
Chromosome
cim
scanone
cim_threshold
scanone_threshold

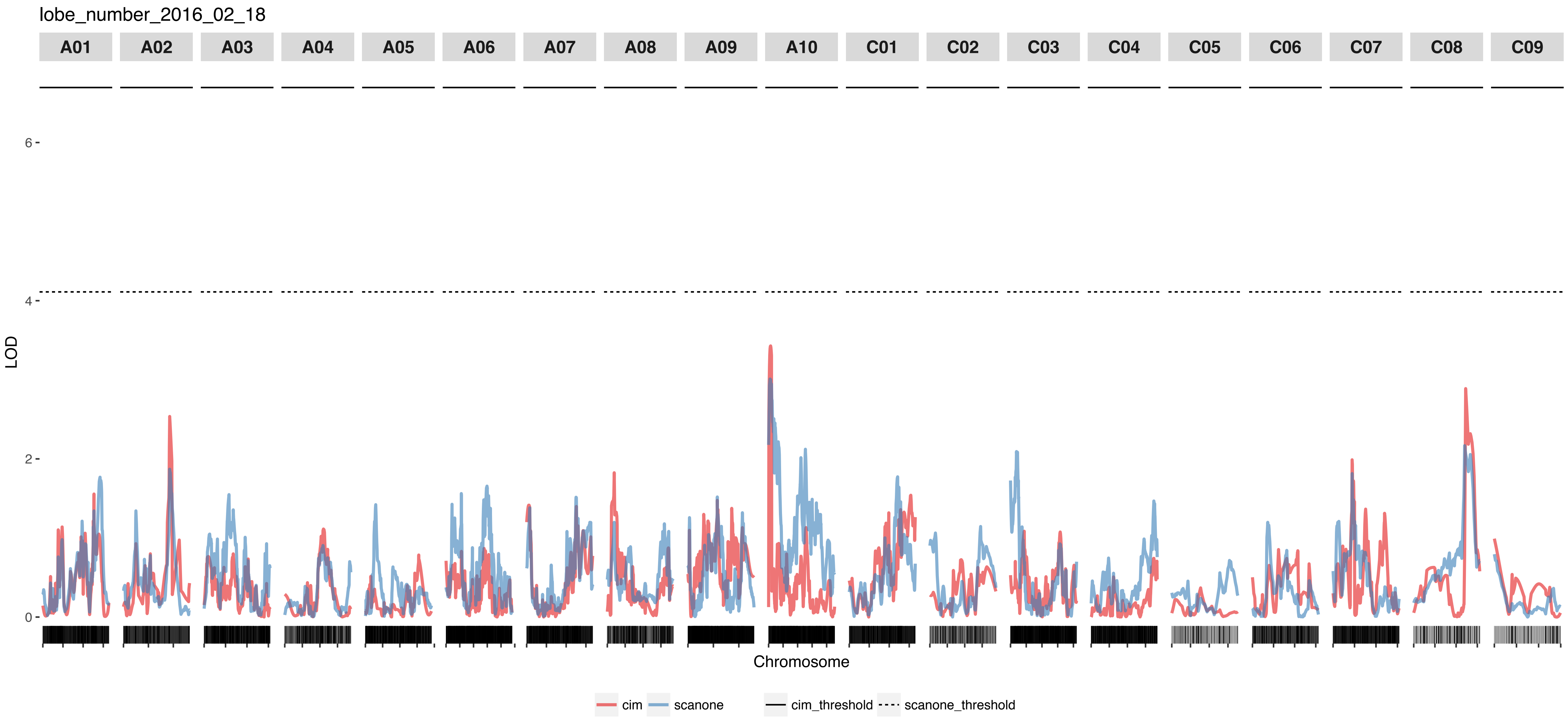

lobe_number_2016_02_18
A01
A02
A03
A04
A05
A06
A07
A08
A09
A10
C01
C02
C03
C04
C05
C06
C07
C08
C09
6
4
2
0
LOD
Chromosome
cim
scanone
cim_threshold
scanone_threshold

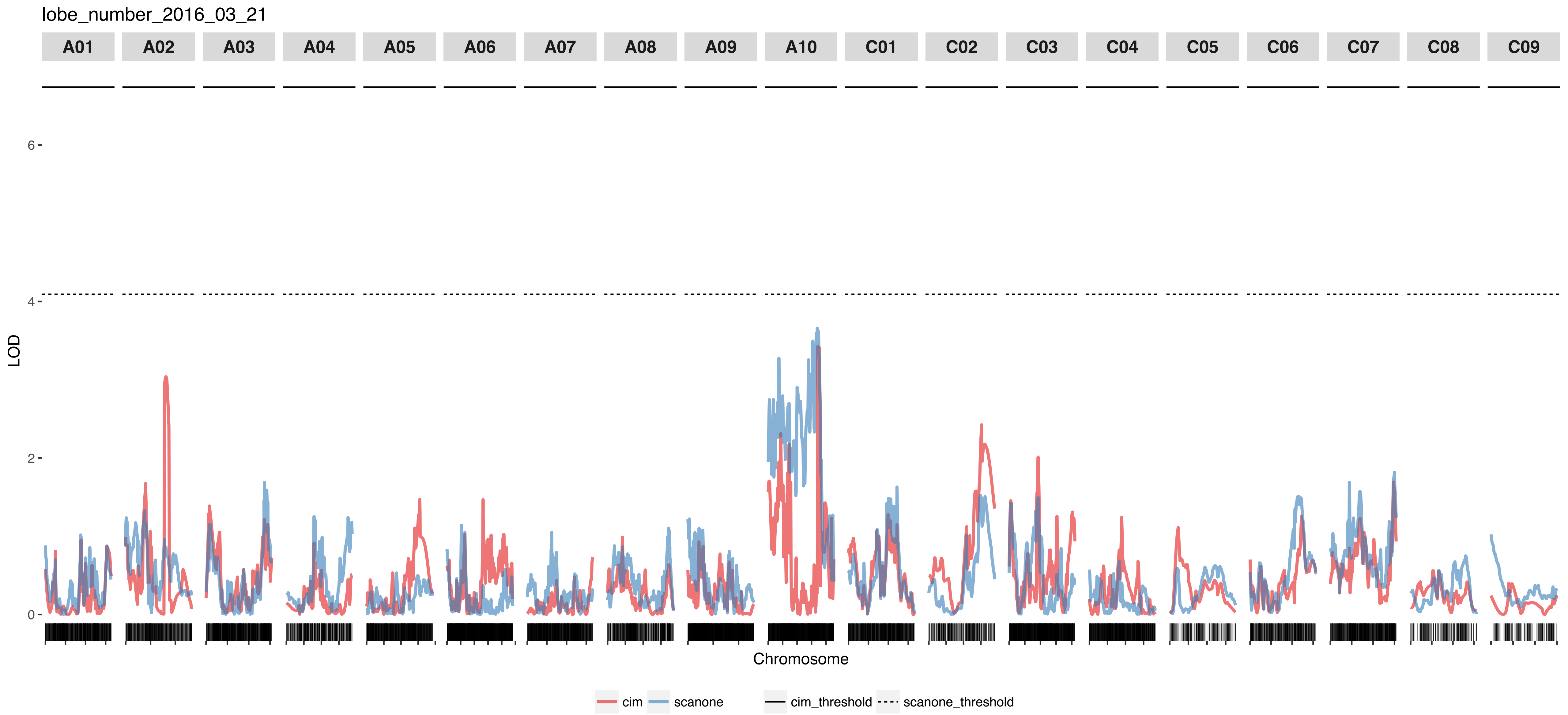

lobe_number_2016_03_21
A01
A02
A03
A04
A05
A06
A07
A08
A09
A10
C01
C02
C03
C04
C05
C06
C07
C08
C09
6
4
2
0
LOD
Chromosome
cim
scanone
cim_threshold
scanone_threshold

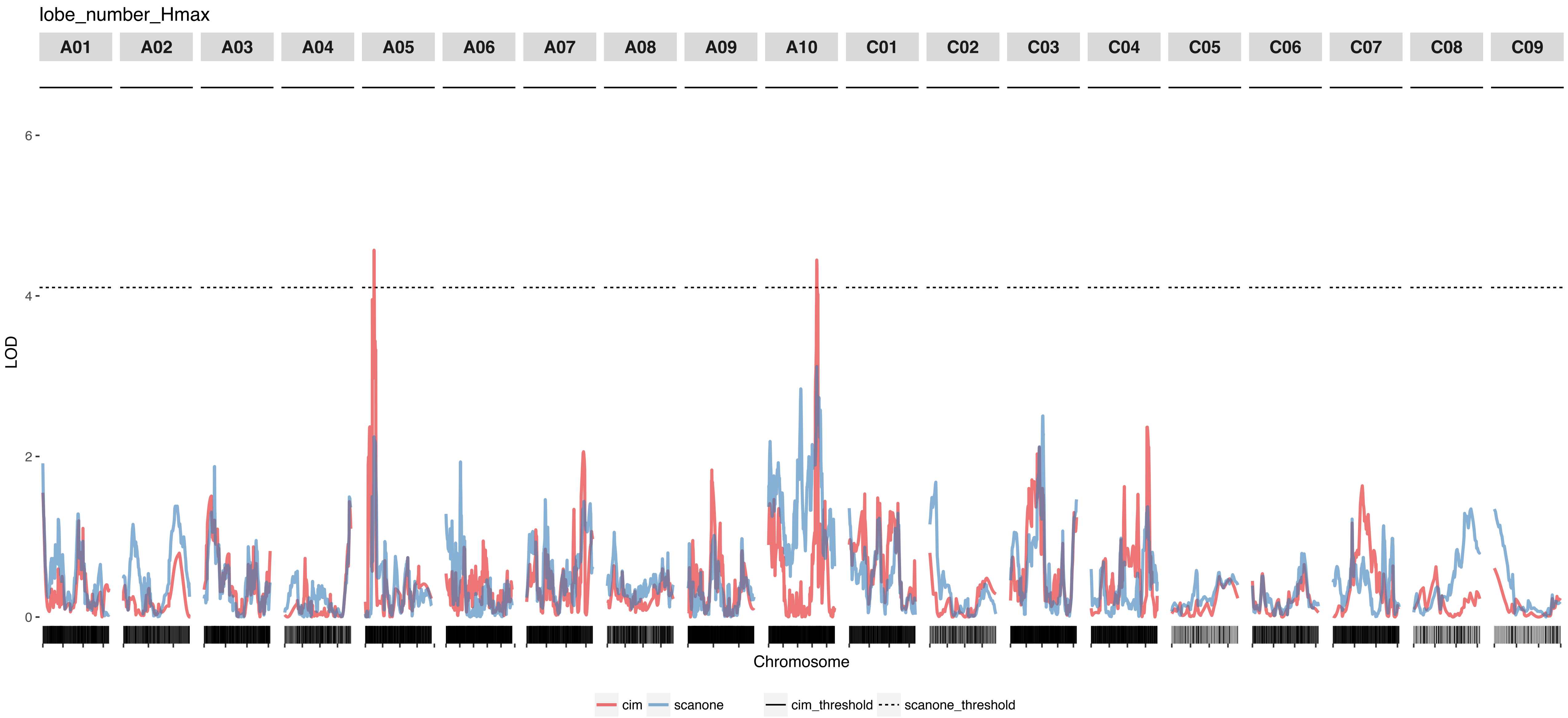

lobe_number_Hmax
A01
A02
A03
A04
A05
A06
A07
A08
A09
A10
C01
C02
C03
C04
C05
C06
C07
C08
C09
LOD
6
4
2
0
Chromosome
cim
scanone
cim_threshold
scanone_threshold

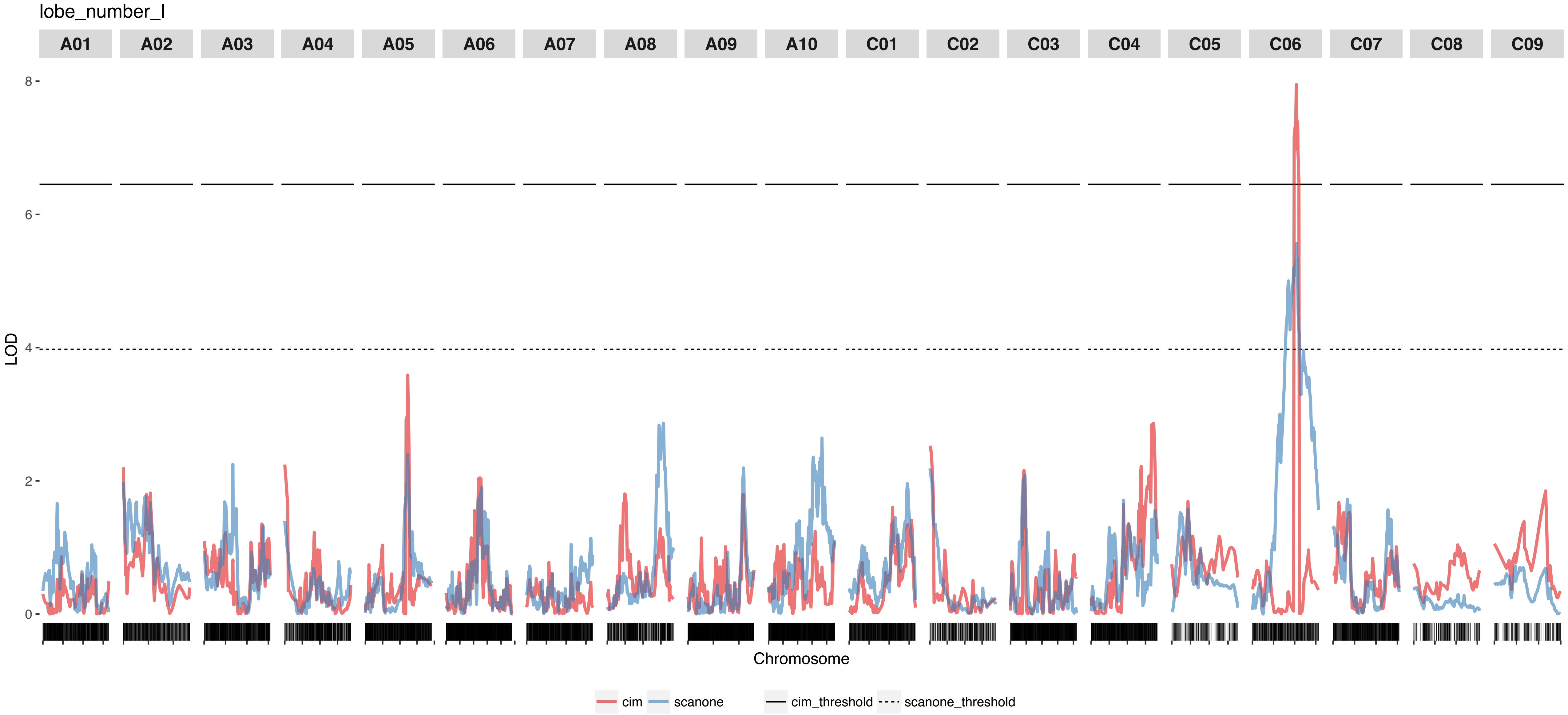

lobe_number_l
A01
A02
A03
A04
A05
A06
A07
A08
A09
A10
C01
C02
C03
C04
C05
C06
C07
C08
C09
LOD
0
2
4
6
8
Chromosome
cim
scanone
cim_threshold
scanone_threshold

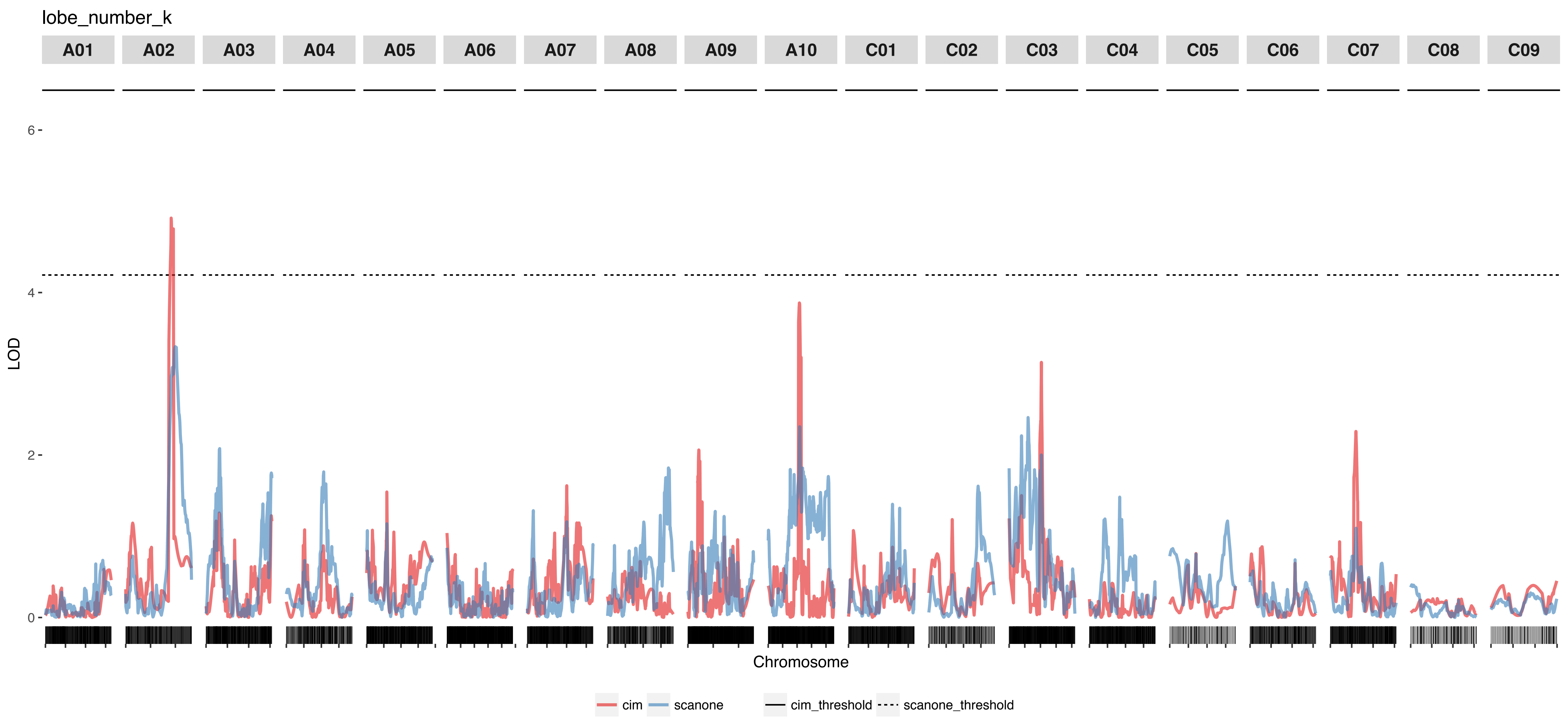

lobe_number_k
A01
A02
A03
A04
A05
A06
A07
A08
A09
A10
C01
C02
C03
C04
C05
C06
C07
C08
C09
LOD
6
4
2
0
Chromosome
cim
scanone
cim_threshold
scanone_threshold

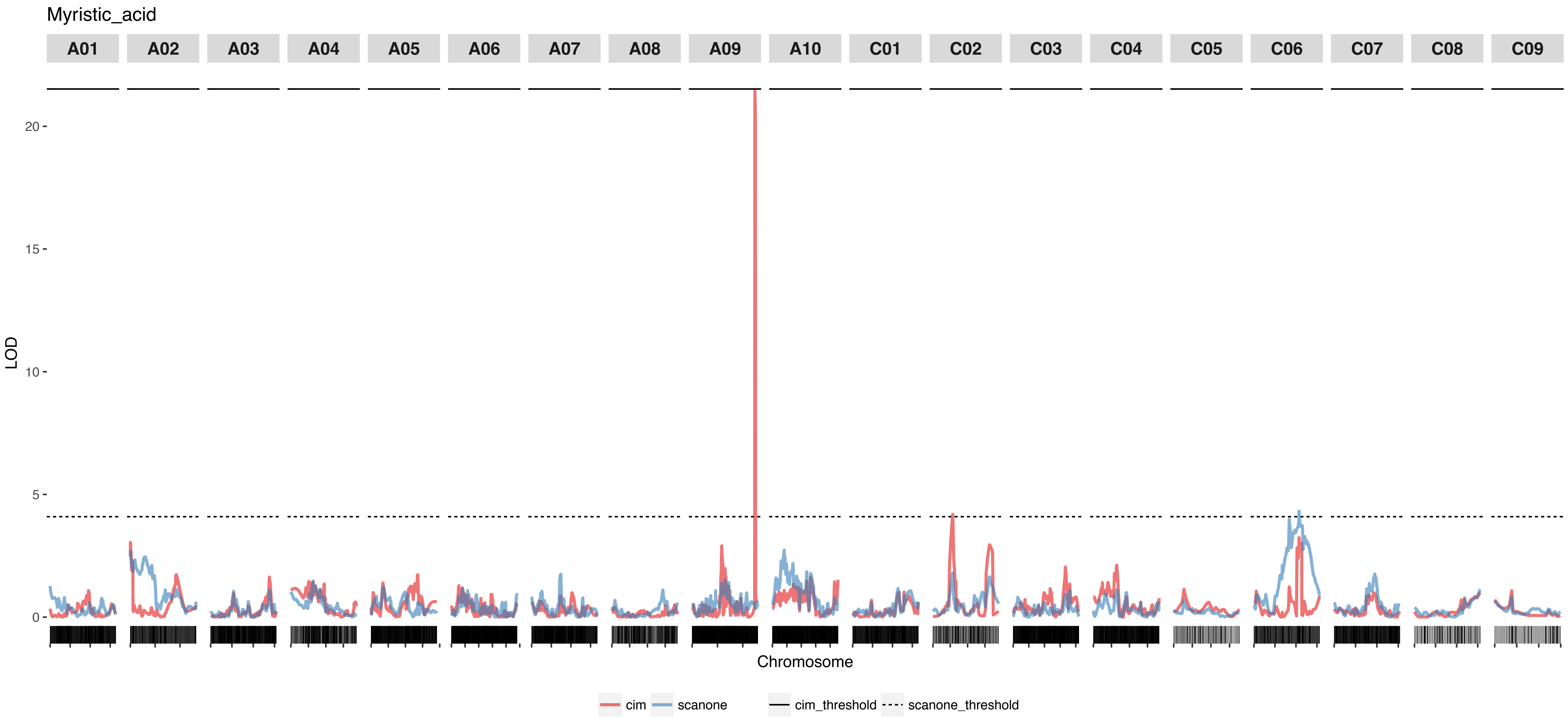

Myristic_acid
A01
A02
A03
A04
A05
A06
A07
A08
A09
A10
C01
C02
C03
C04
C05
C06
C07
C08
C09
LOD
0
5
10
15
20
Chromosome
cim
scanone
cim_threshold
scanone_threshold

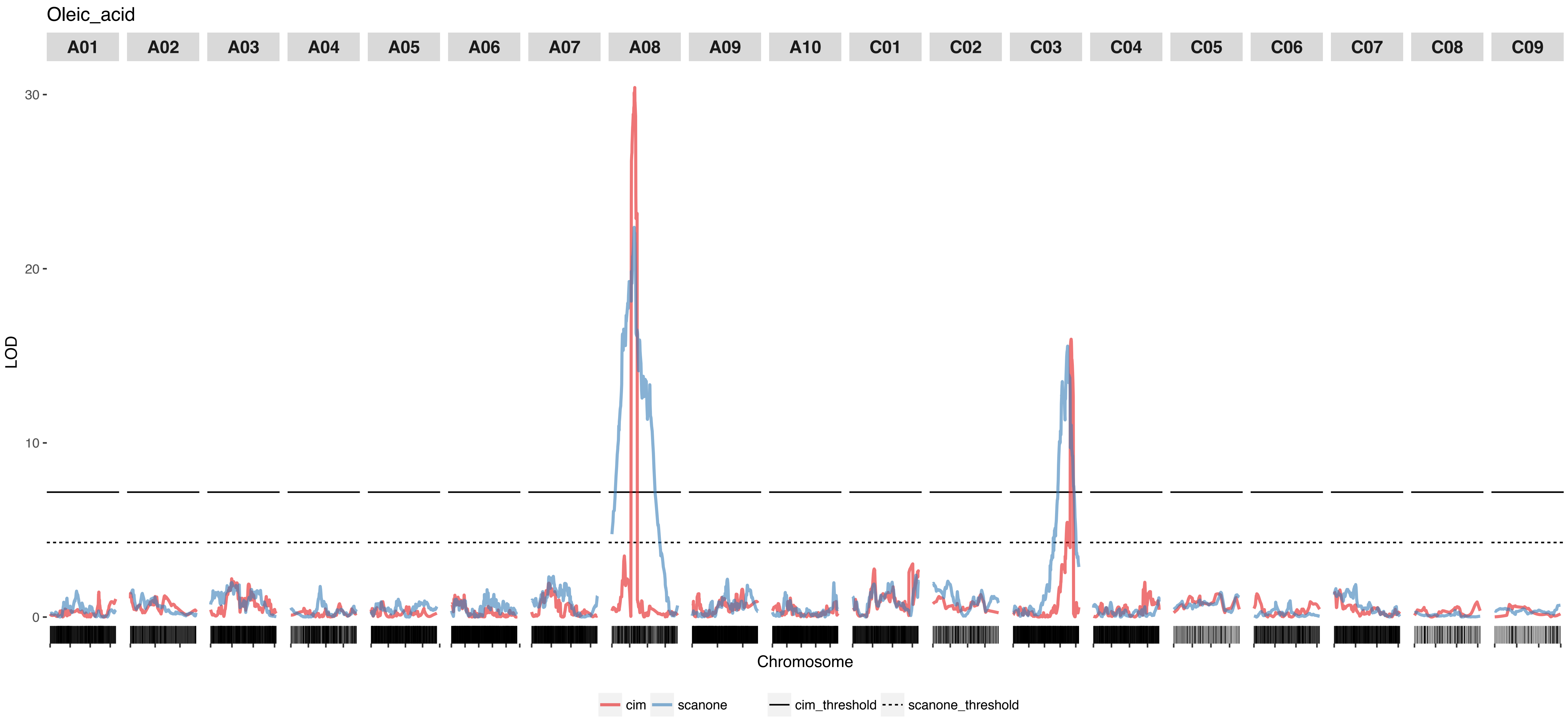

Oleic_acid
A01
A02
A03
A04
A05
A06
A07
A08
A09
A10
C01
C02
C03
C04
C05
C06
C07
C08
C09
30
20
10
0
LOD
Chromosome
cim
scanone
cim_threshold
scanone_threshold

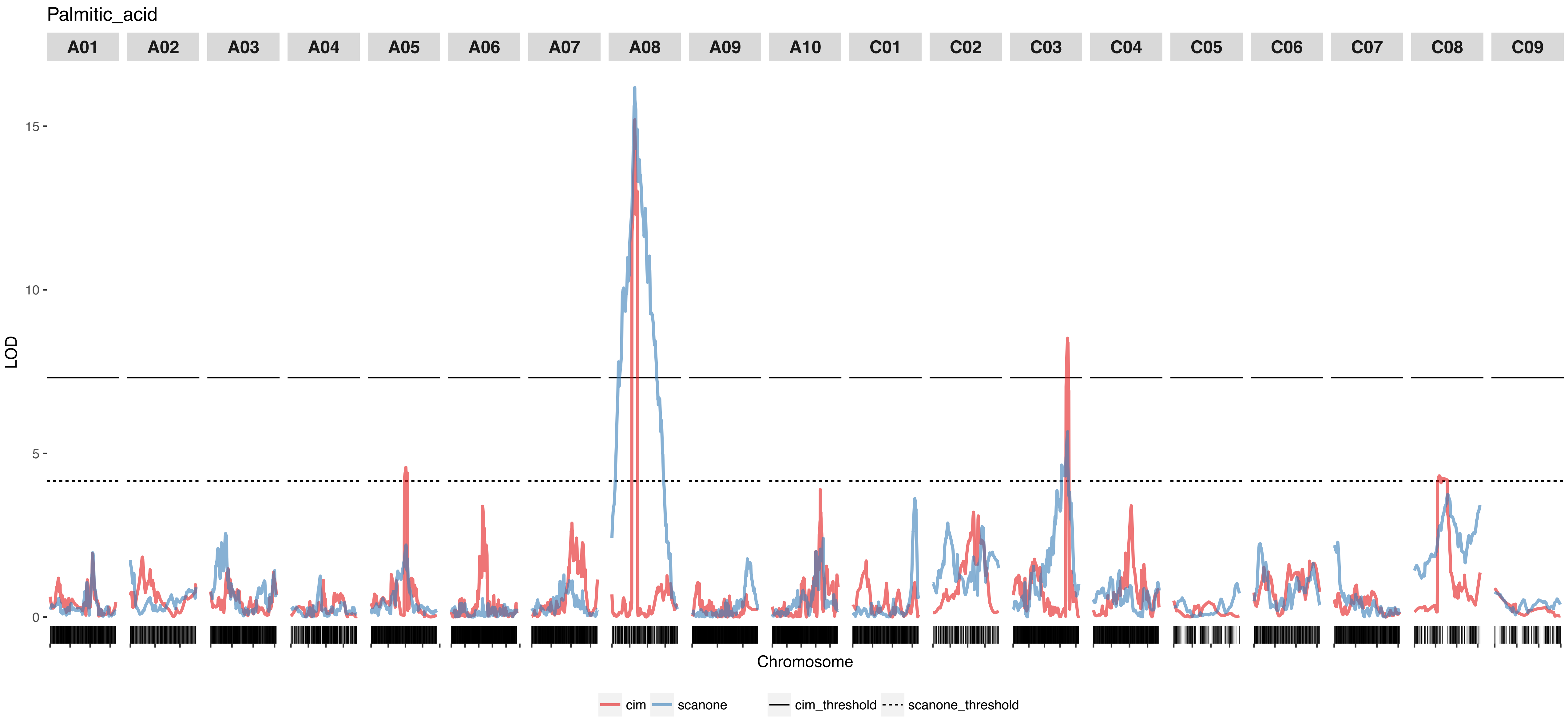
Palmitic_acid
A01
A02
A03
A04
A05
A06
A07
A08
A09
A10
C01
C02
C03
C04
C05
C06
C07
C08
C09
15
10
5
0
LOD
Chromosome
cim
scanone
cim_threshold
scanone_threshold

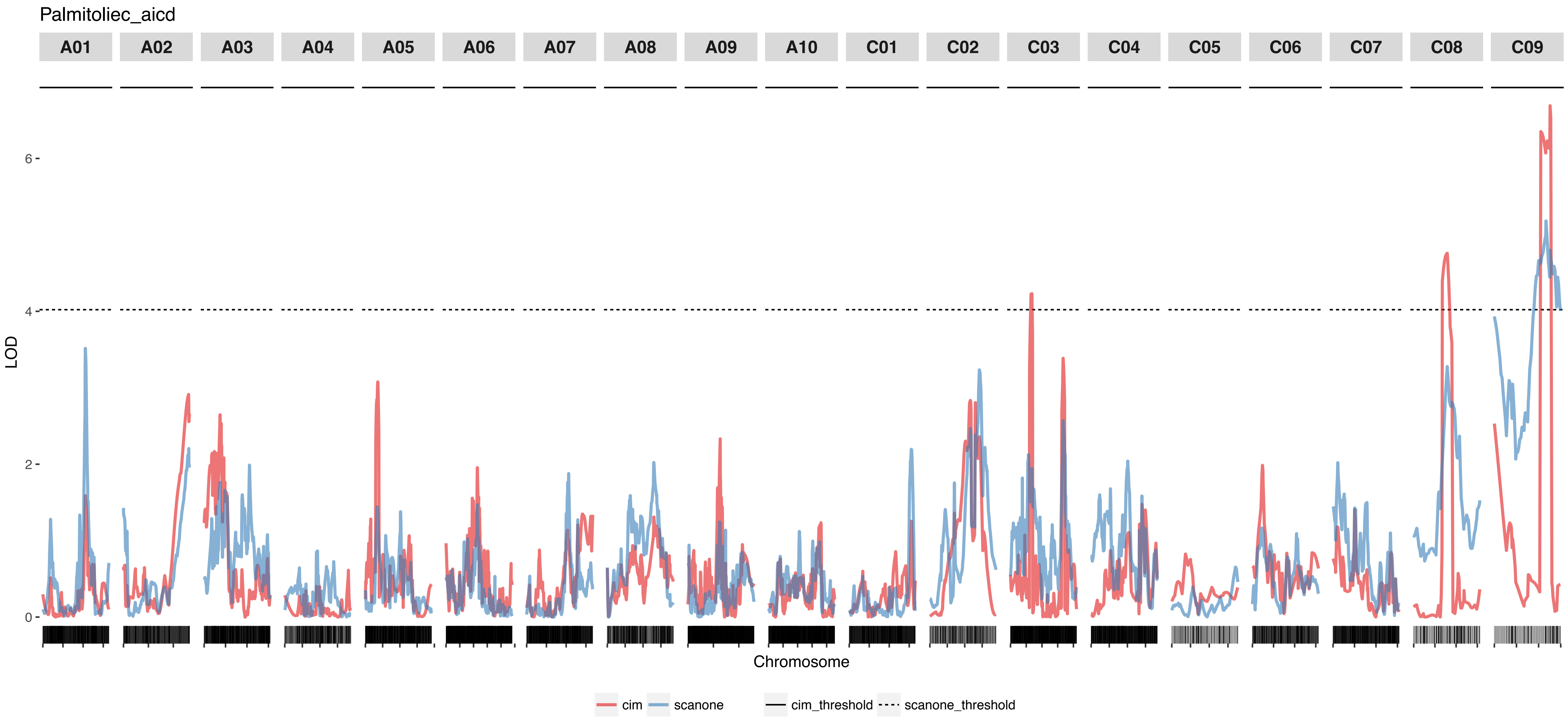

Palmitoliec_aicd
A01
A02
A03
A04
A05
A06
A07
A08
A09
A10
C01
C02
C03
C04
C05
C06
C07
C08
C09
LOD
6
4
2
0
Chromosome
cim
scanone
cim_threshold
scanone_threshold

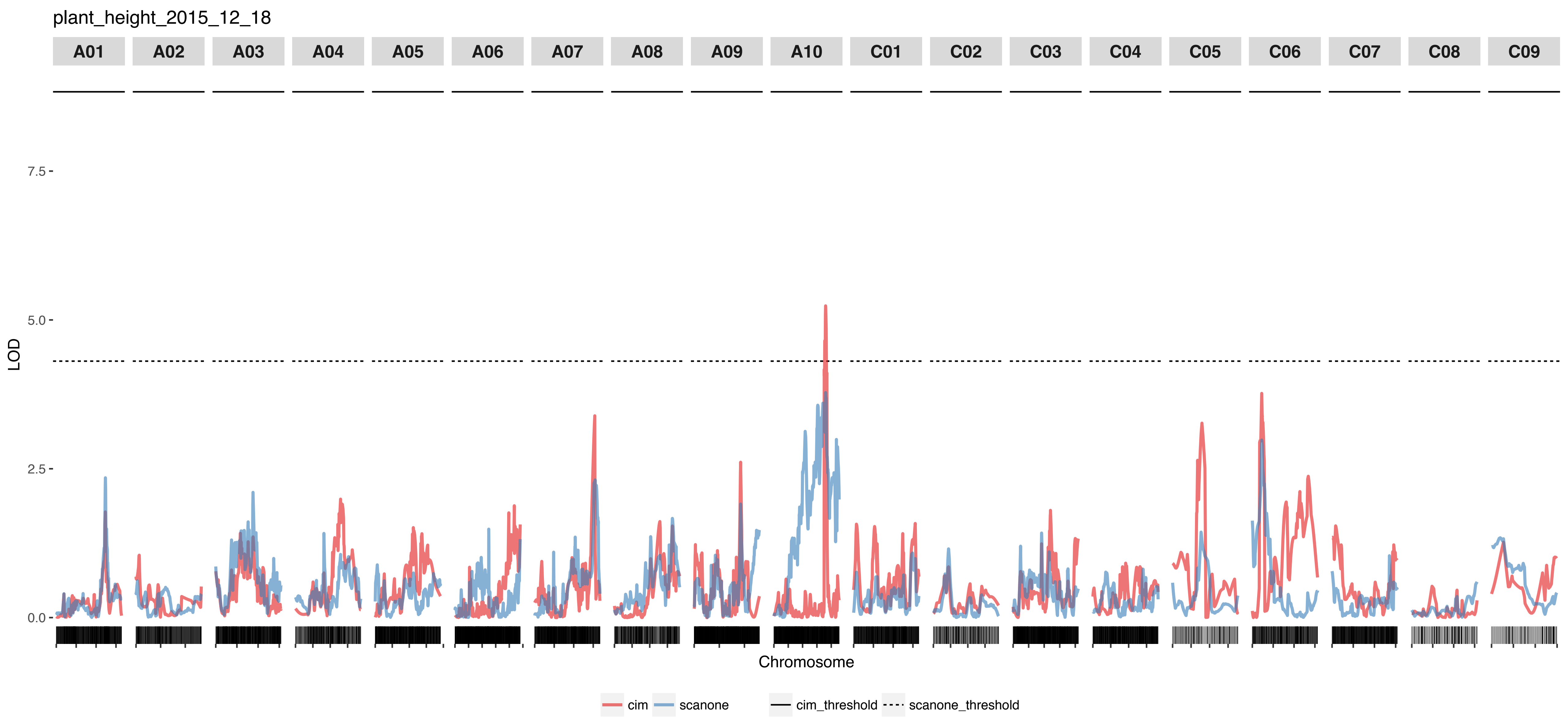

plant_height_2015_12_18
A01
A02
A03
A04
A05
A06
A07
A08
A09
A10
C01
C02
C03
C04
C05
C06
C07
C08
C09
7.5
5.0
2.5
0.0
LOD
Chromosome
cim
scanone
cim_threshold
scanone_threshold

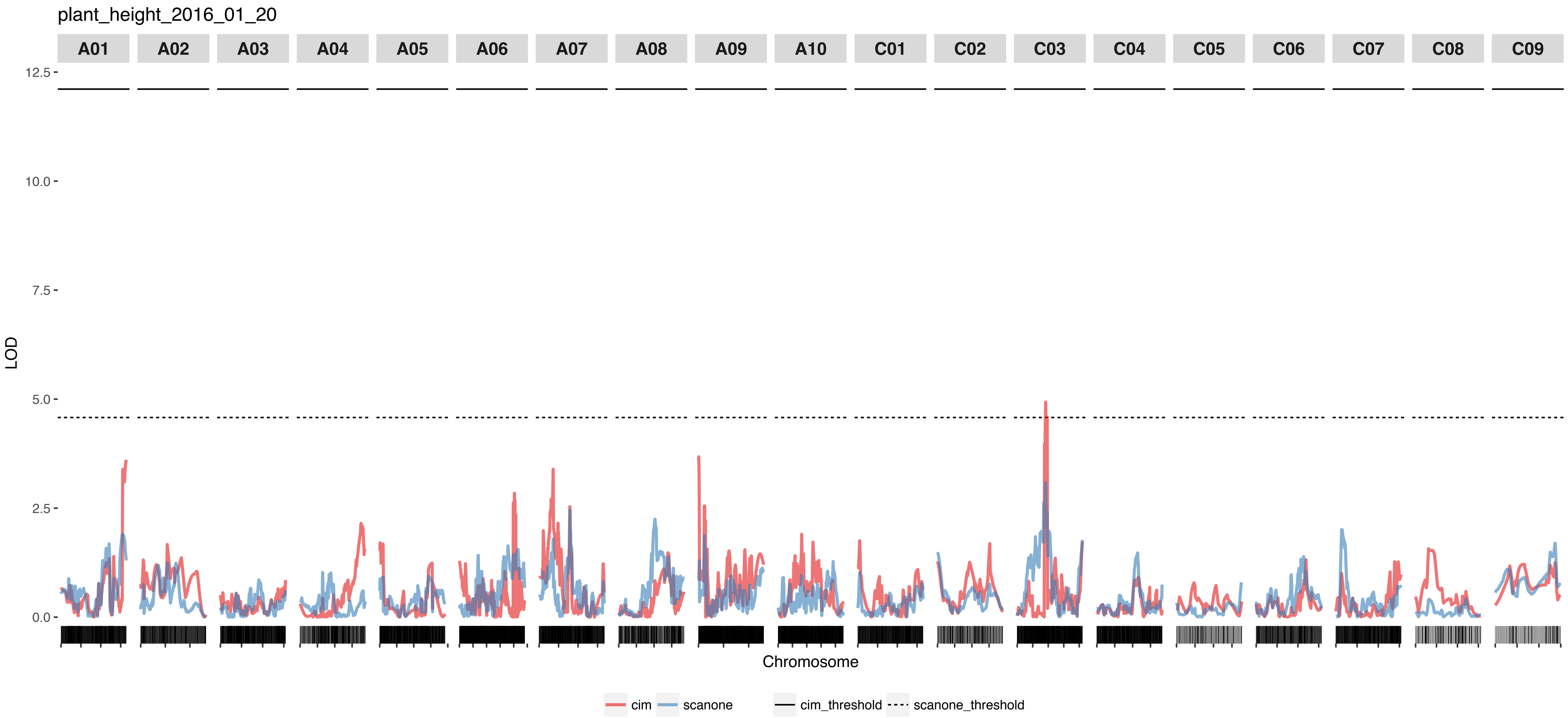

plant_height_2016_01_20
A01
A02
A03
A04
A05
A06
A07
A08
A09
A10
C01
C02
C03
C04
C05
C06
C07
C08
C09
12.5
10.0
7.5
5.0
2.5
0.0
LOD
Chromosome
cim
scanone
cim_threshold
scanone_threshold

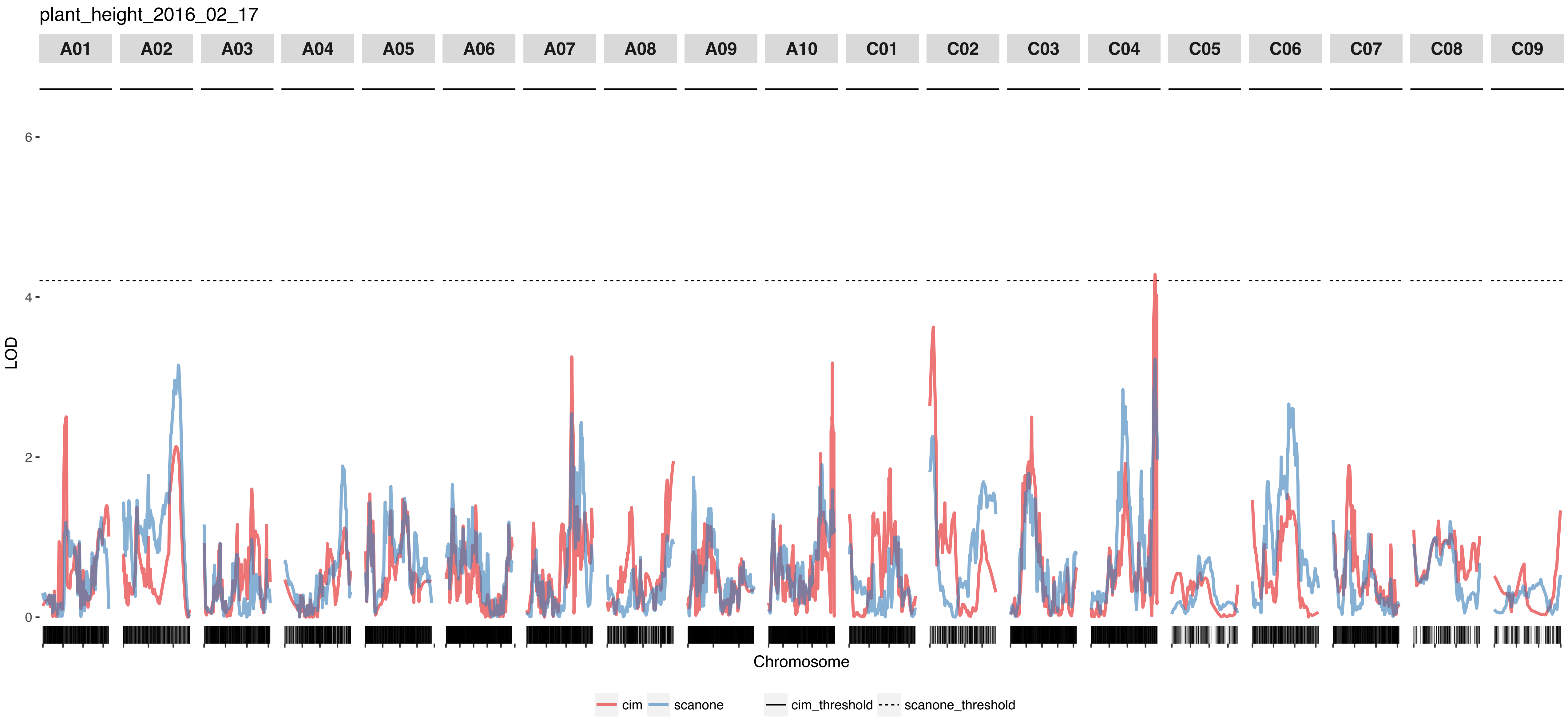

plant_height_2016_02_17
A01
A02
A03
A04
A05
A06
A07
A08
A09
A10
C01
C02
C03
C04
C05
C06
C07
C08
C09
LOD
6
4
2
0
Chromosome
cim
scanone
cim_threshold
scanone_threshold

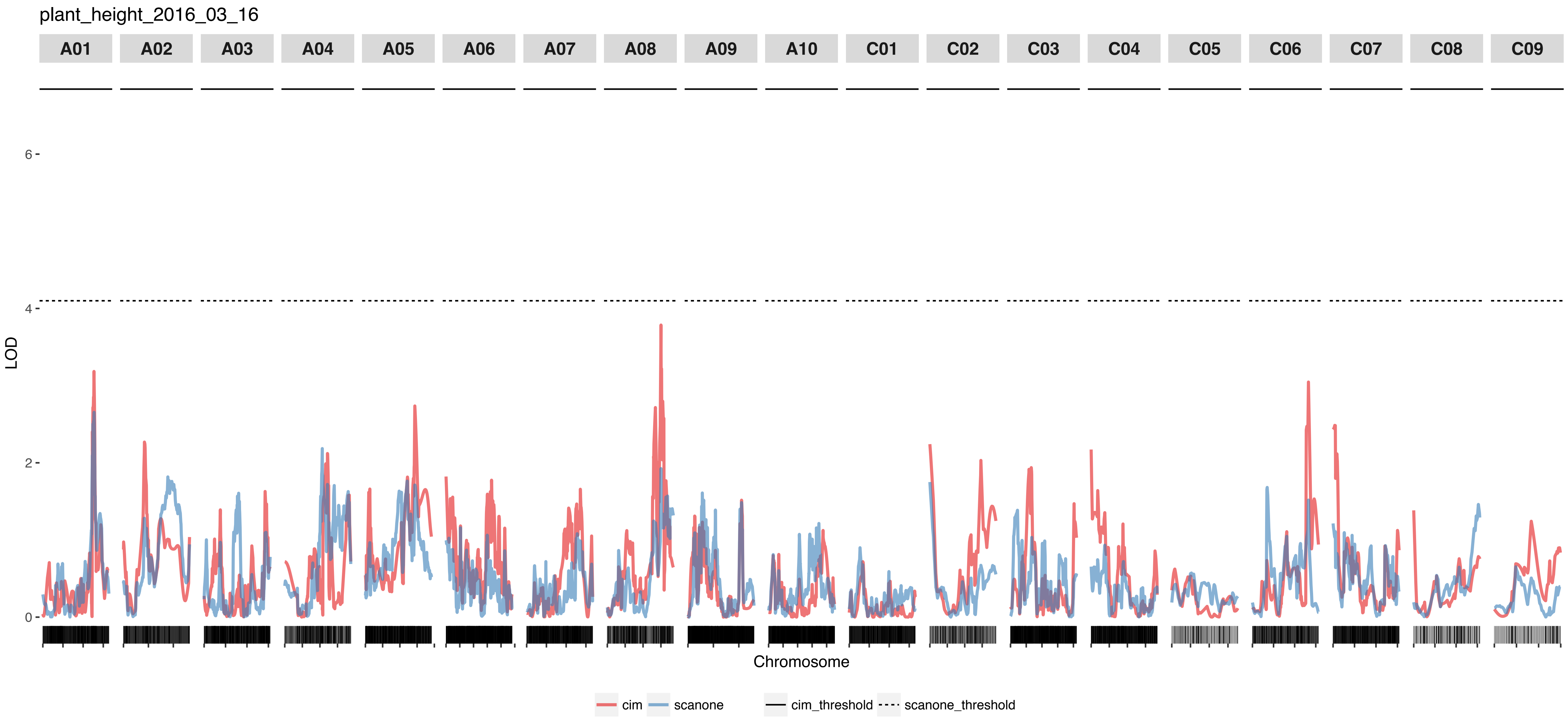
plant_height_2016_03_16
A01
A02
A03
A04
A05
A06
A07
A08
A09
A10
C01
C02
C03
C04
C05
C06
C07
C08
C09
LOD
6
4
2
0
Chromosome
cim
scanone
cim_threshold
scanone_threshold

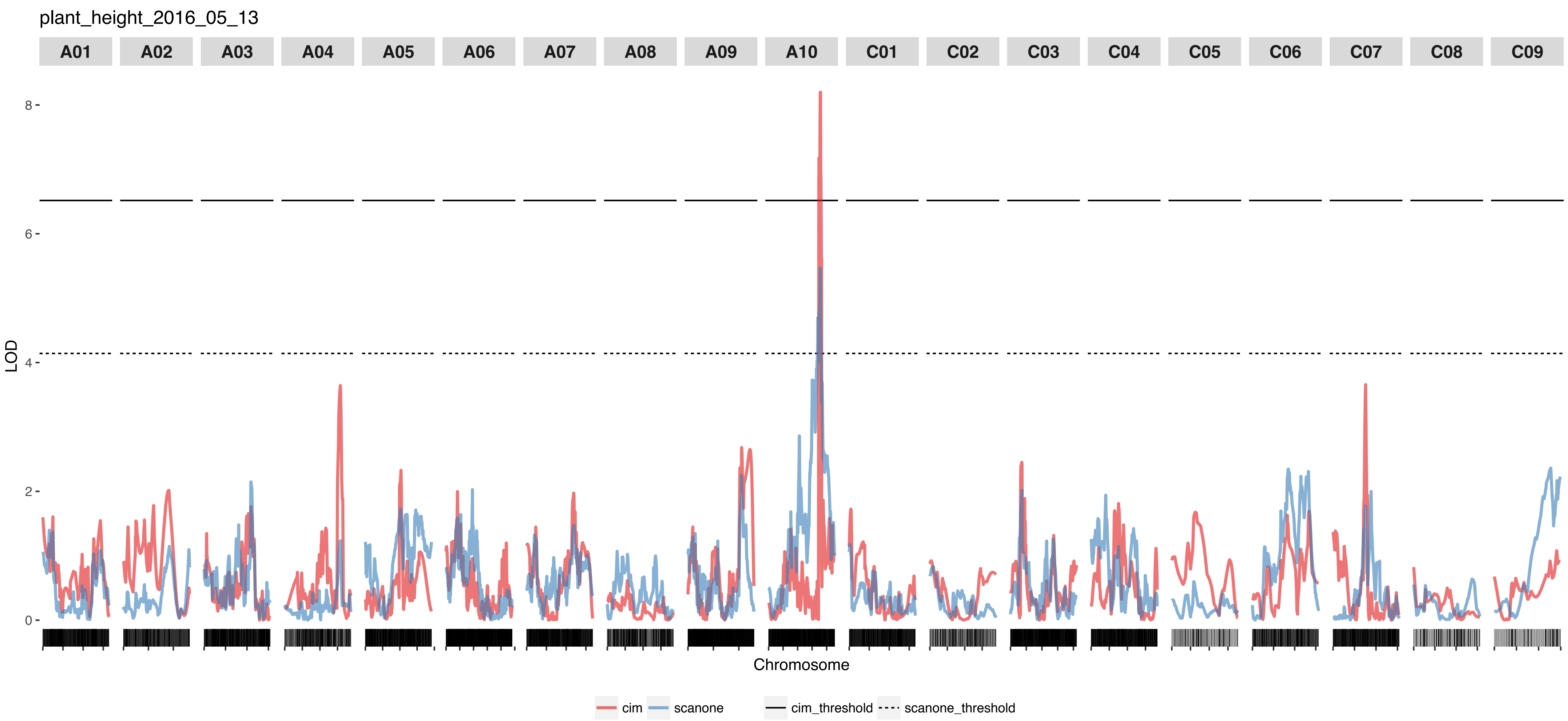
plant_height_2016_05_13
A01
A02
A03
A04
A05
A06
A07
A08
A09
A10
C01
C02
C03
C04
C05
C06
C07
C08
C09
LOD
8
6
4
2
0
Chromosome
cim
scanone
cim_threshold
scanone_threshold

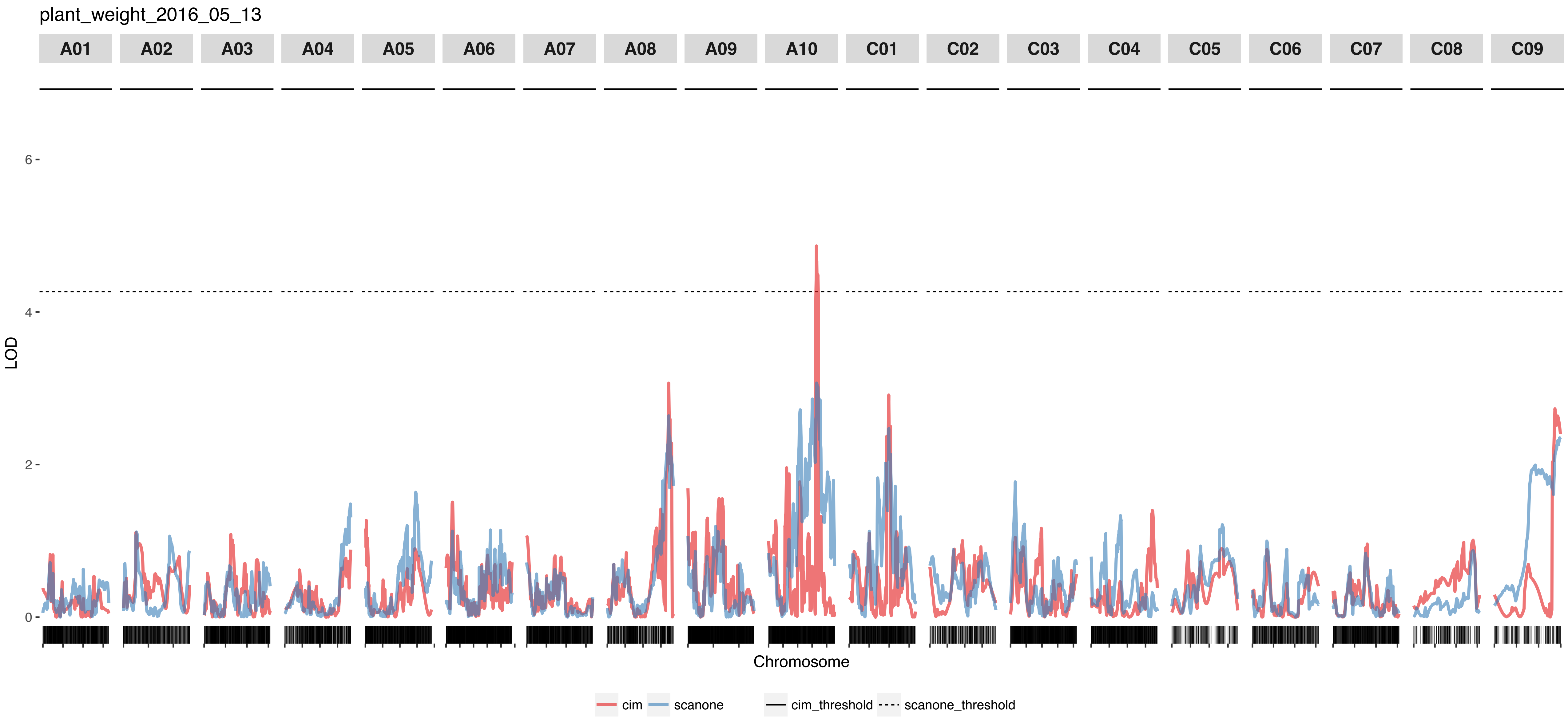

plant_weight_2016_05_13
A01
A02
A03
A04
A05
A06
A07
A08
A09
A10
C01
C02
C03
C04
C05
C06
C07
C08
C09
LOD
6
4
2
0
Chromosome
cim
scanone
cim_threshold
scanone_threshold

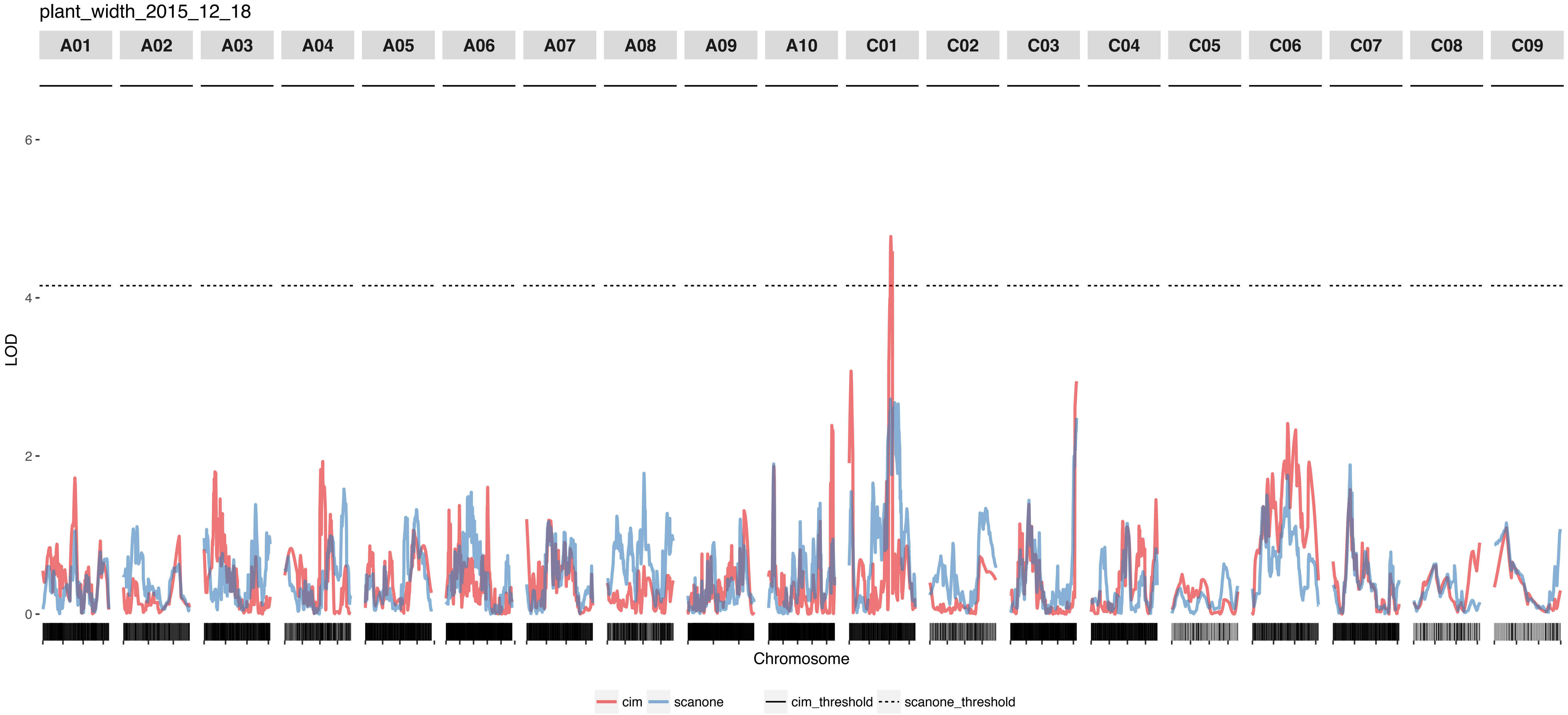
plant_width_2015_12_18
A01
A02
A03
A04
A05
A06
A07
A08
A09
A10
C01
C02
C03
C04
C05
C06
C07
C08
C09
LOD
6
4
2
0
Chromosome
cim
scanone
cim_threshold
scanone_threshold

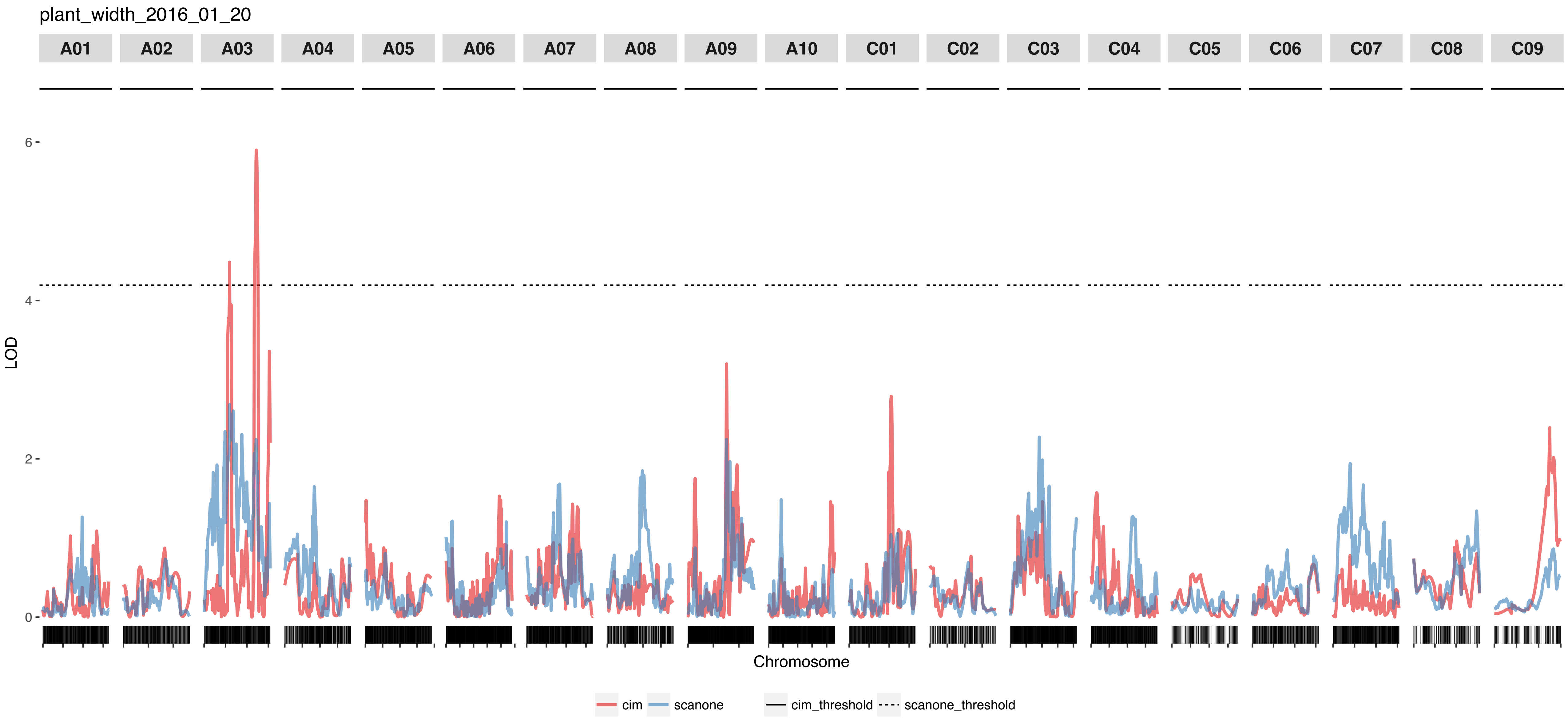

plant_width_2016_01_20
A01
A02
A03
A04
A05
A06
A07
A08
A09
A10
C01
C02
C03
C04
C05
C06
C07
C08
C09
LOD
6
4
2
0
Chromosome
cim
scanone
cim_threshold
scanone_threshold

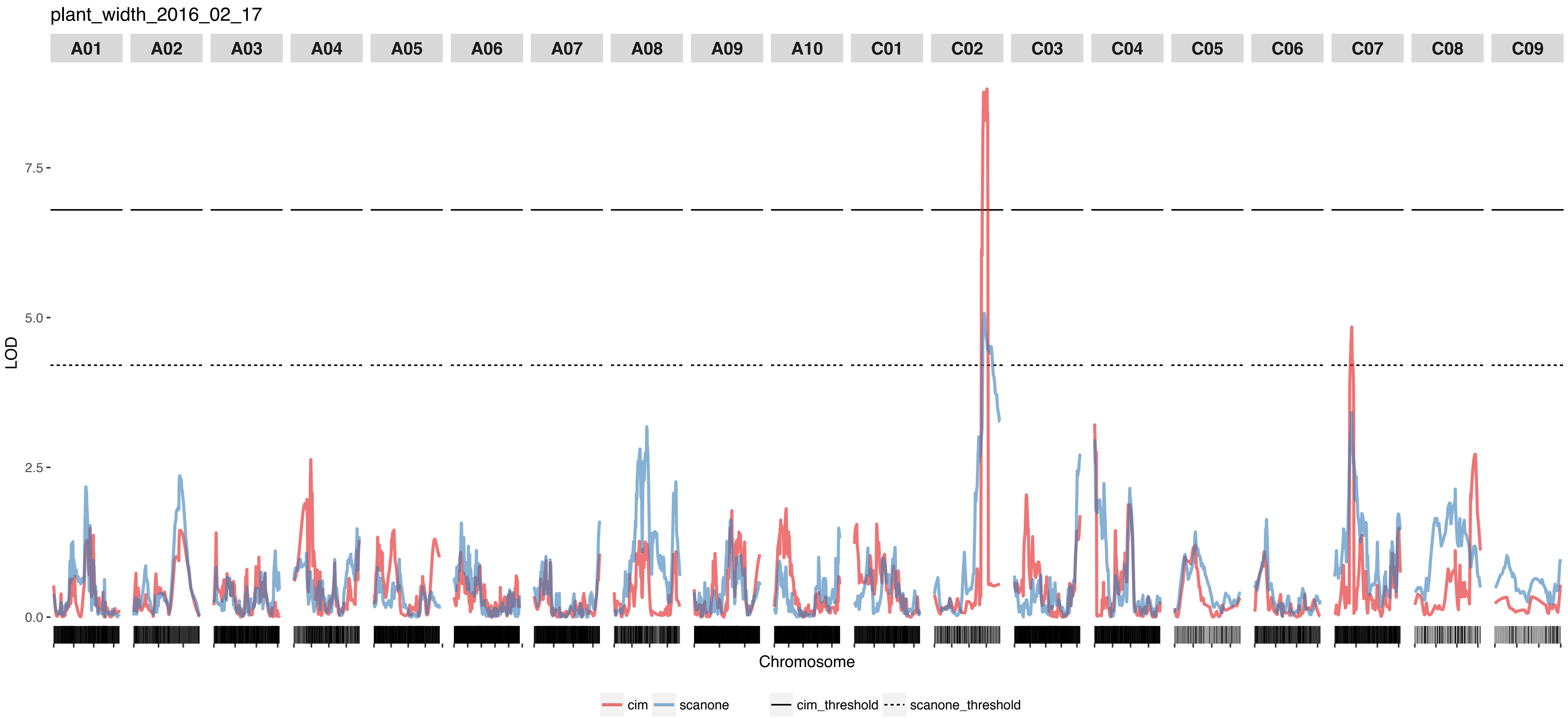
plant_width_2016_02_17
A01
A02
A03
A04
A05
A06
A07
A08
A09
A10
C01
C02
C03
C04
C05
C06
C07
C08
C09
LOD
7.5
5.0
2.5
0.0
Chromosome
cim
scanone
cim_threshold
scanone_threshold

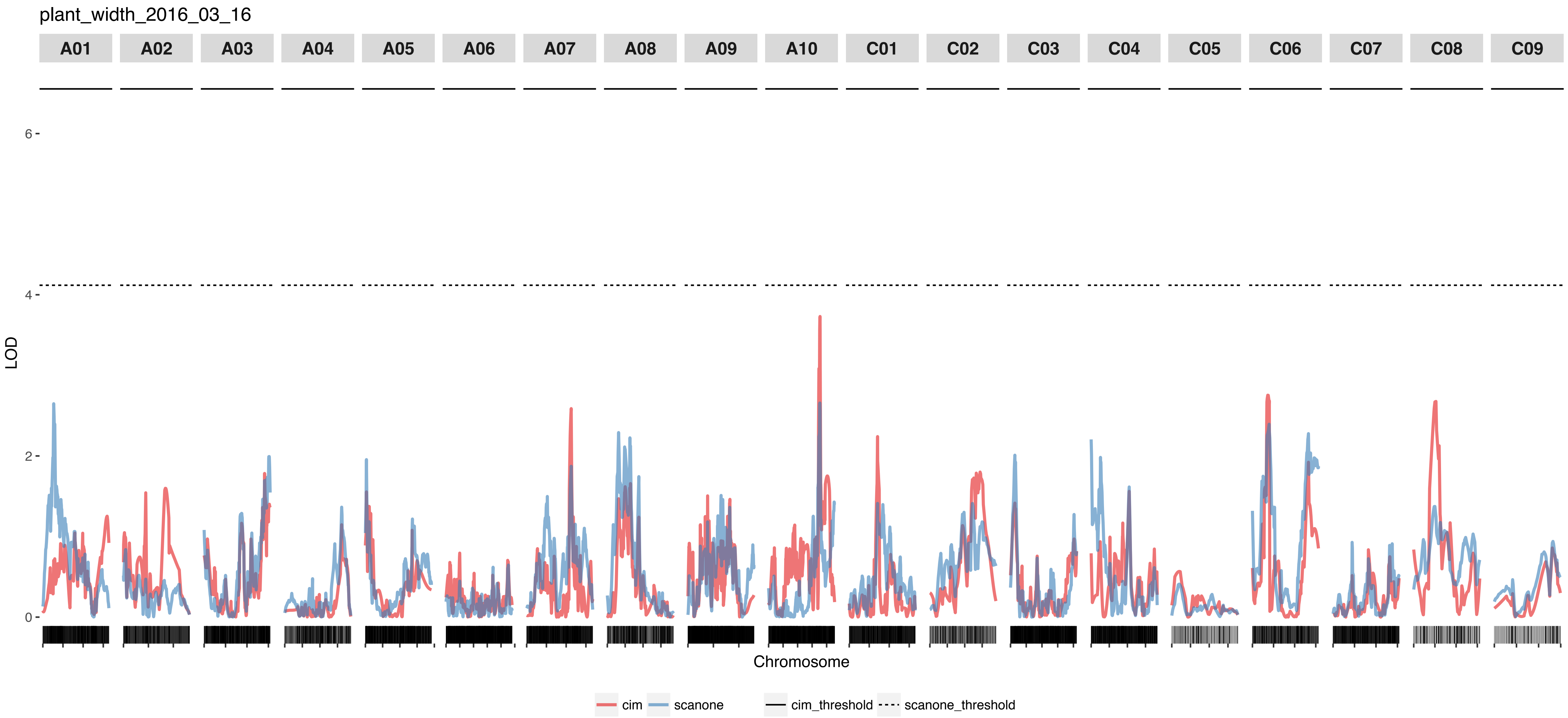
plant_width_2016_03_16
A01
A02
A03
A04
A05
A06
A07
A08
A09
A10
C01
C02
C03
C04
C05
C06
C07
C08
C09
6
4
2
0
LOD
Chromosome
cim
scanone
cim_threshold
scanone_threshold

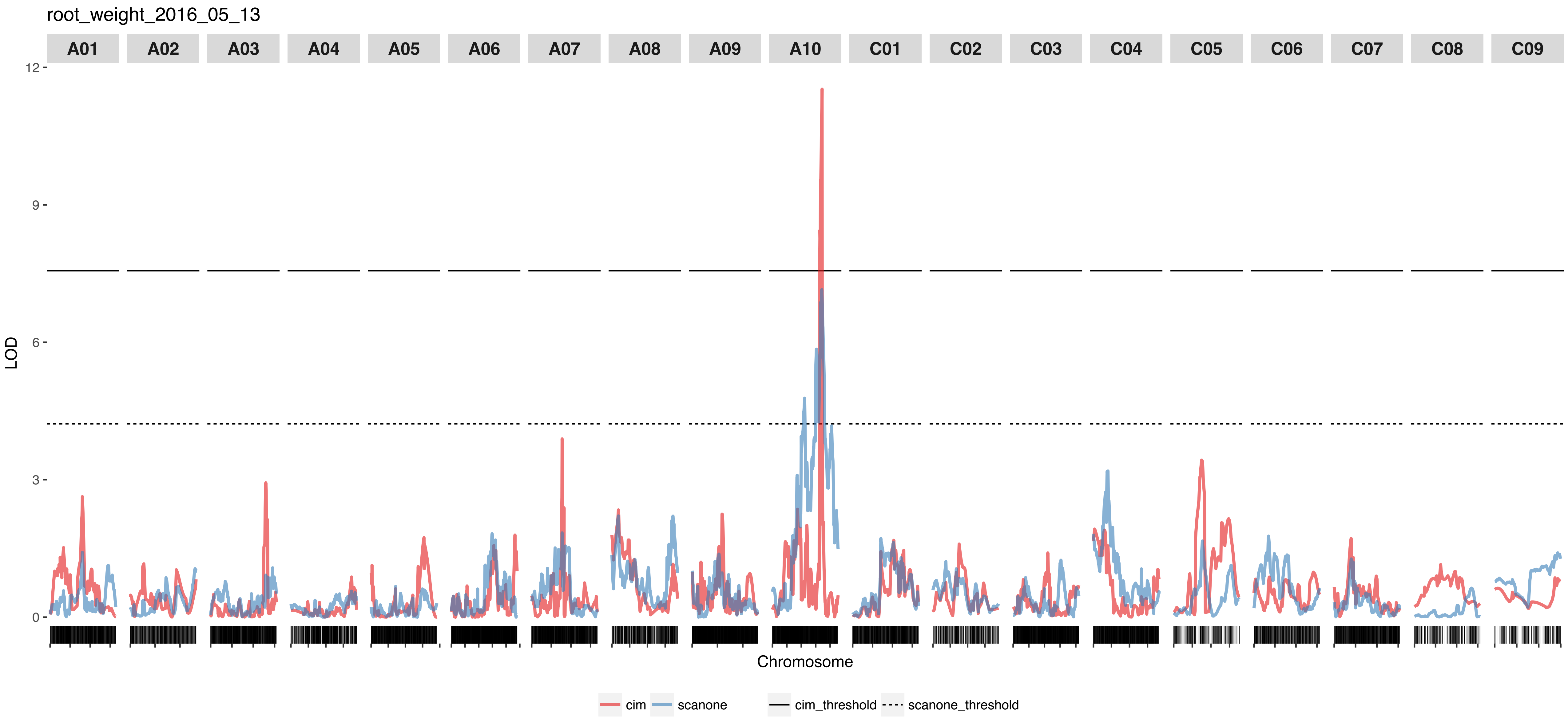
root_weight_2016_05_13
A01
A02
A03
A04
A05
A06
A07
A08
A09
A10
C01
C02
C03
C04
C05
C06
C07
C08
C09
12
9
6
3
0
LOD
Chromosome
cim
scanone
cim_threshold
scanone_threshold

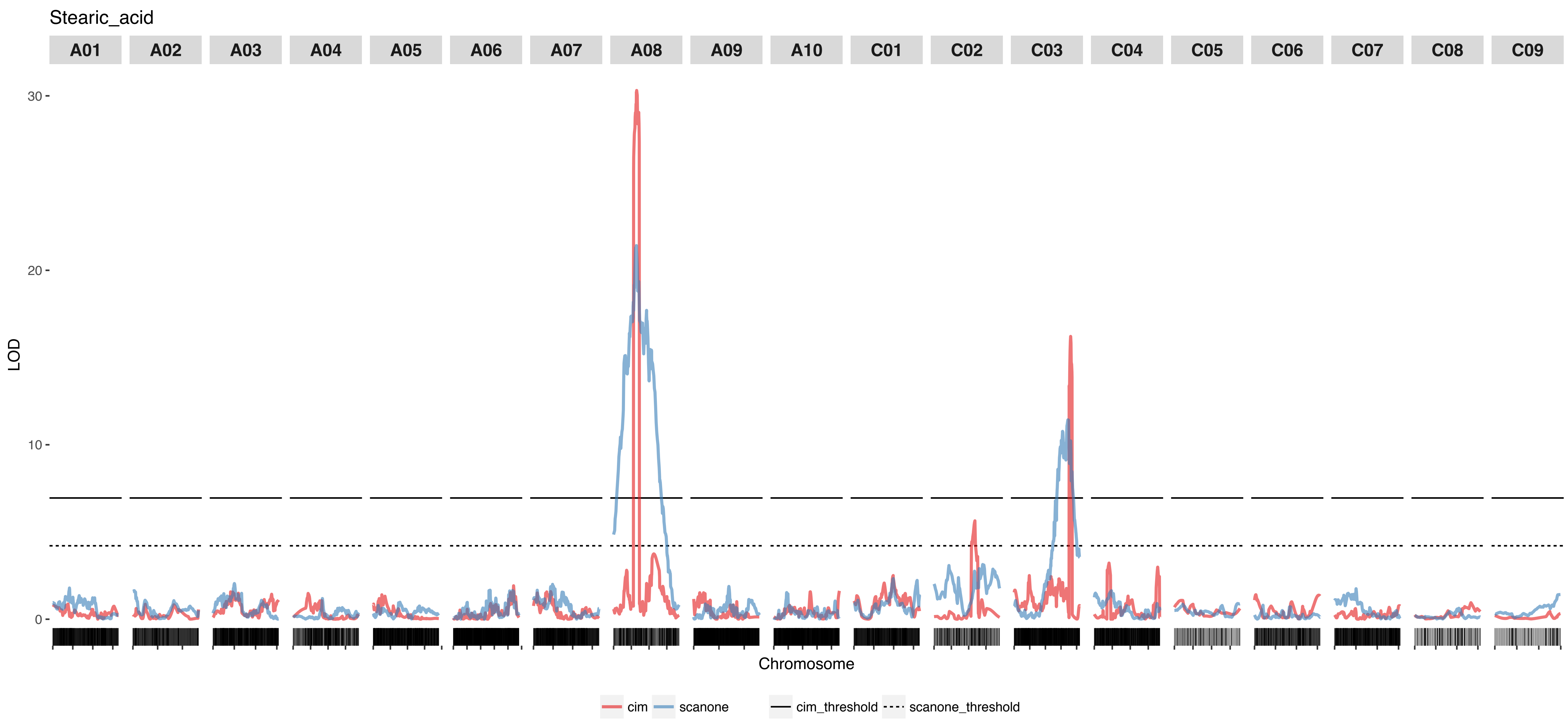

Stearic_acid
A01
A02
A03
A04
A05
A06
A07
A08
A09
A10
C01
C02
C03
C04
C05
C06
C07
C08
C09
30
20
10
0
LOD
Chromosome
cim
scanone
cim_threshold
scanone_threshold

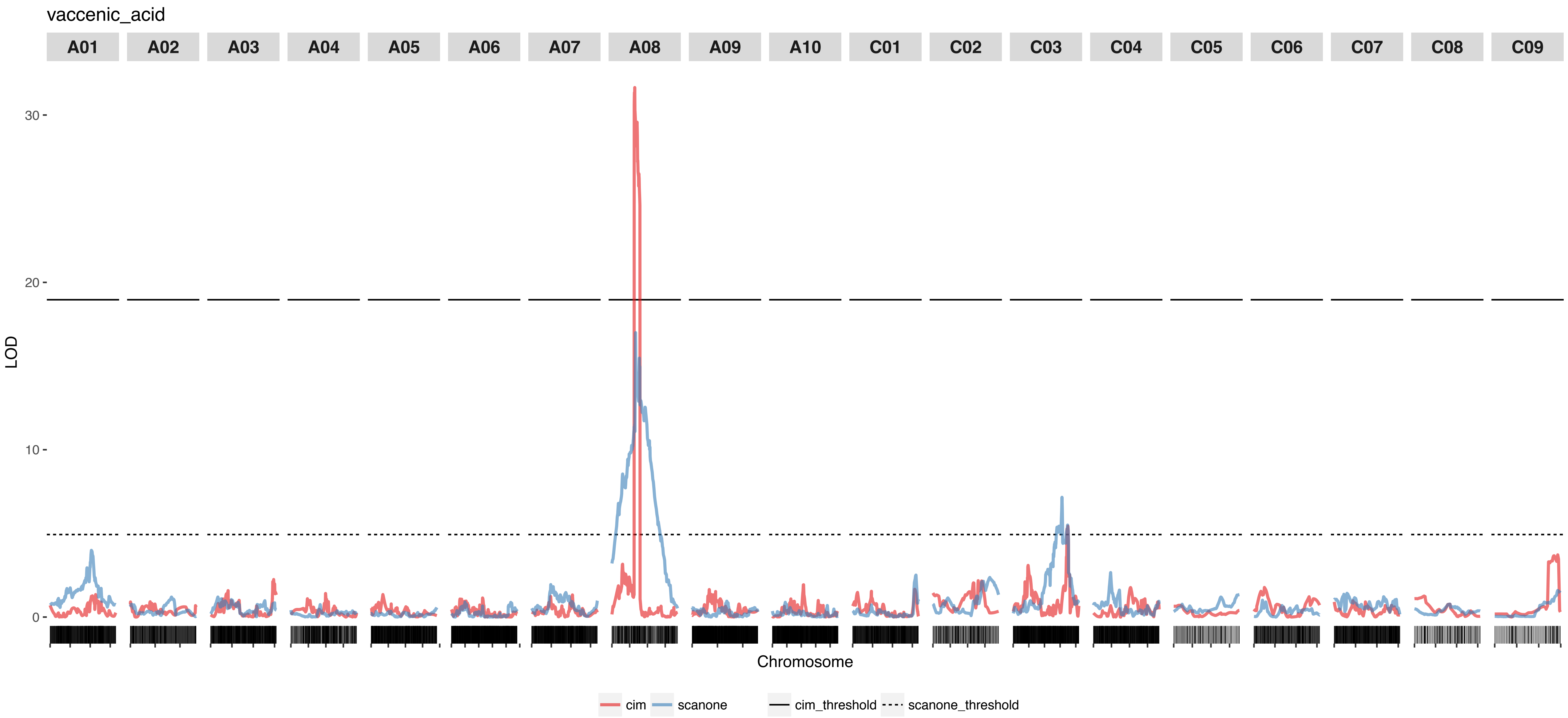

vaccenic_acid
A01
A02
A03
A04
A05
A06
A07
A08
A09
A10
C01
C02
C03
C04
C05
C06
C07
C08
C09
30
20
10
0
LOD
Chromosome
cim
scanone
cim_threshold
scanone_threshold

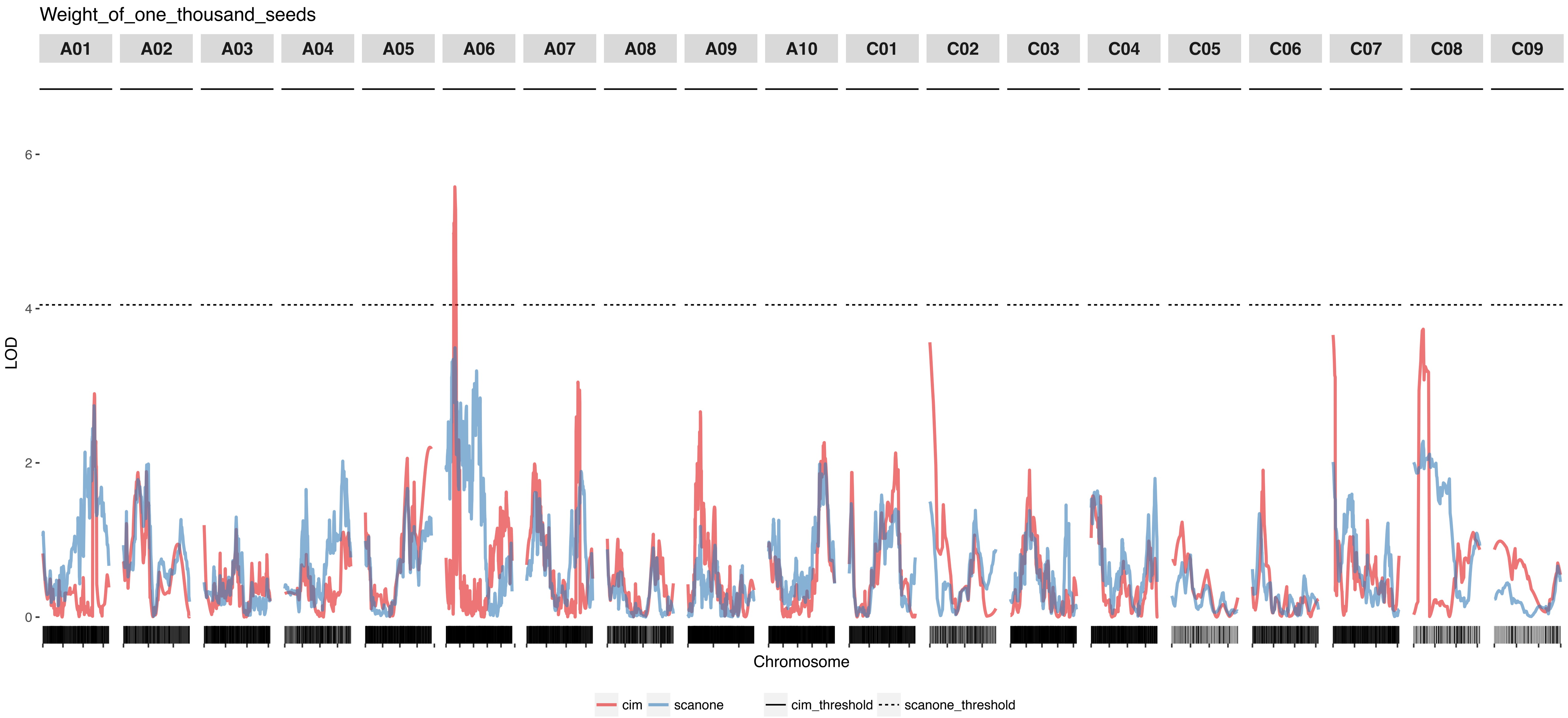

Weight_of_one_thousand_seeds
A01
A02
A03
A04
A05
A06
A07
A08
A09
A10
C01
C02
C03
C04
C05
C06
C07
C08
C09
LOD
6
4
2
0
Chromosome
cim
scanone
cim_threshold
scanone_threshold

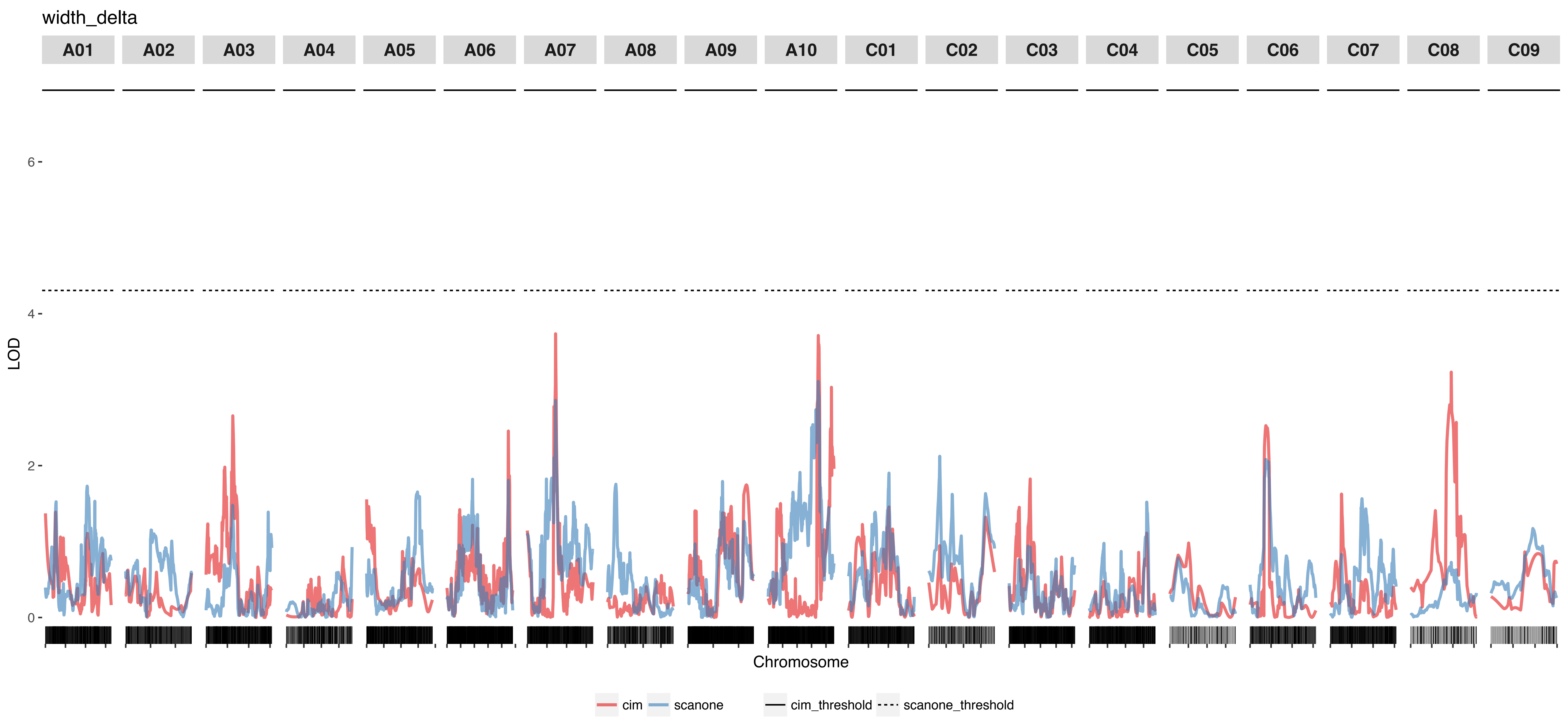
width_delta
A01
A02
A03
A04
A05
A06
A07
A08
A09
A10
C01
C02
C03
C04
C05
C06
C07
C08
C09
6
4
2
0
LOD
Chromosome
cim
scanone
cim_threshold
scanone_threshold

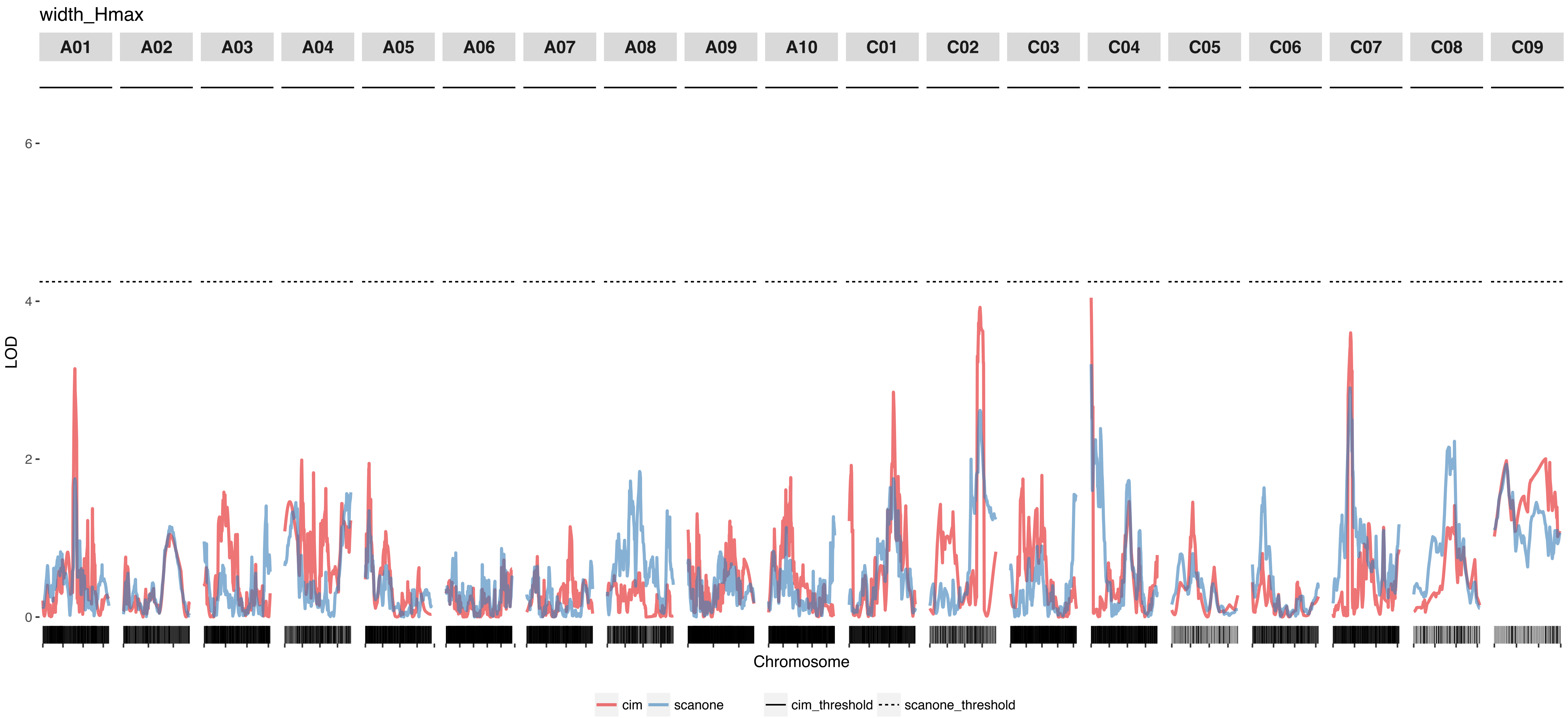

width_Hmax
A01
A02
A03
A04
A05
A06
A07
A08
A09
A10
C01
C02
C03
C04
C05
C06
C07
C08
C09
6
4
2
0
LOD
Chromosome
cim
scanone
cim_threshold
scanone_threshold

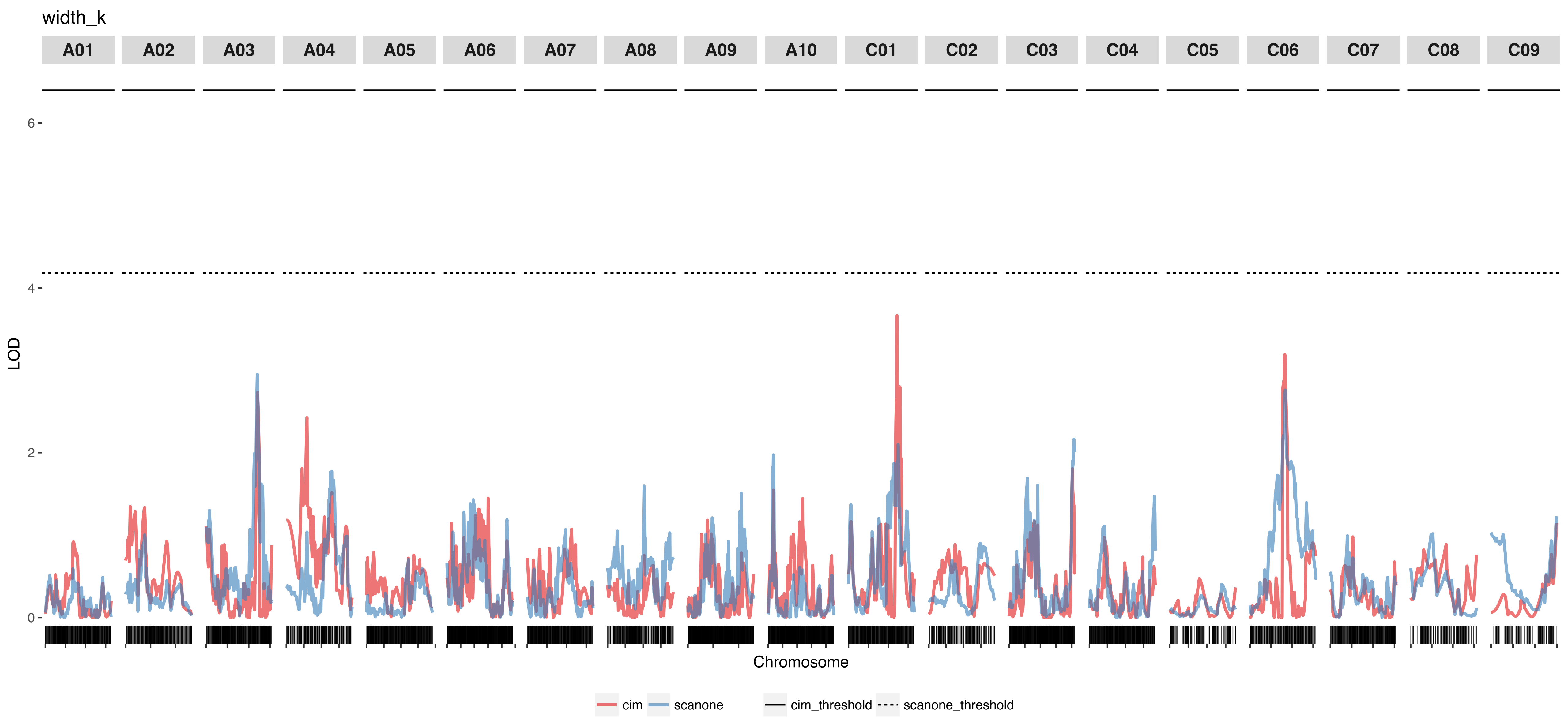
width_k
A01
A02
A03
A04
A05
A06
A07
A08
A09
A10
C01
C02
C03
C04
C05
C06
C07
C08
C09
LOD
6
4
2
0
Chromosome
cim
scanone
cim_threshold
scanone_threshold

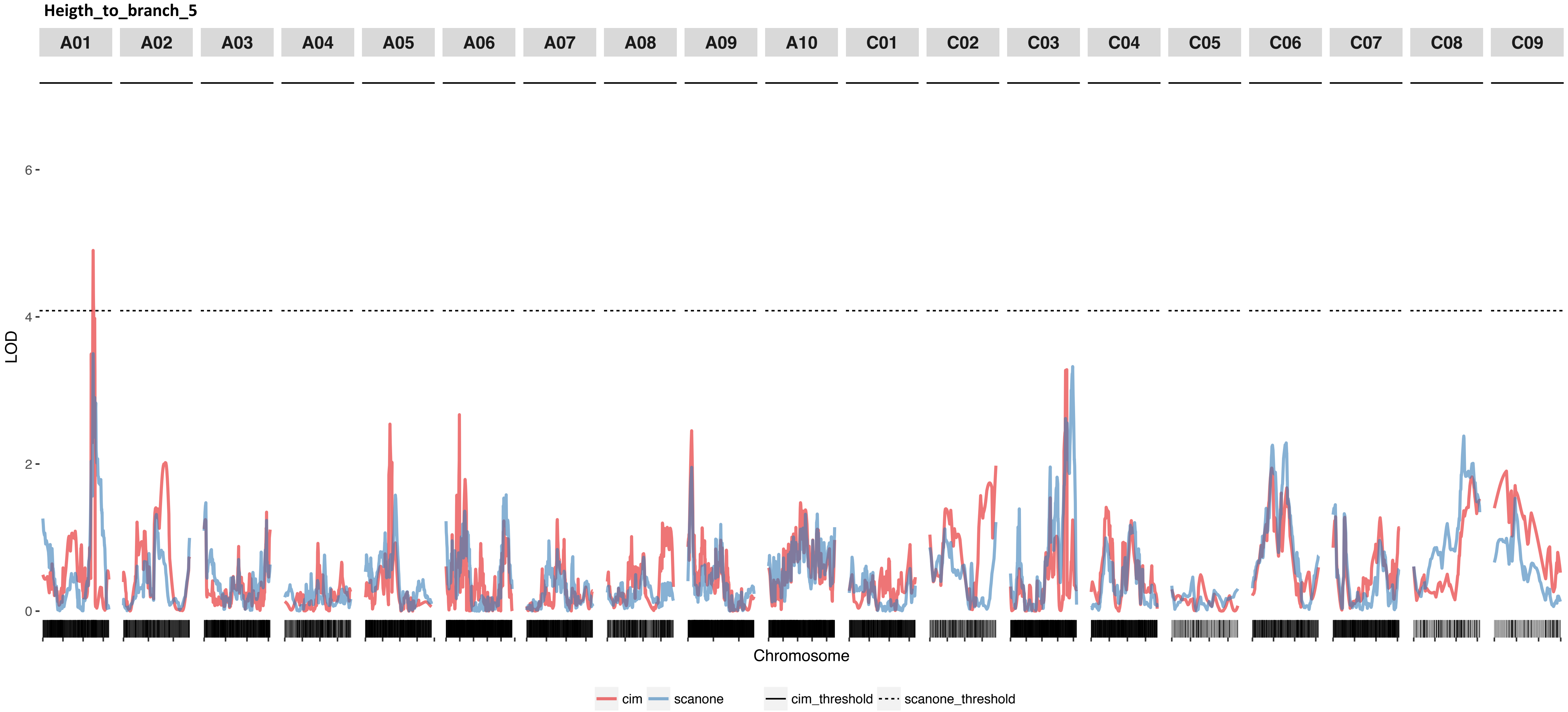
Heigth_to_branch_5
A01
A02
A03
A04
A05
A06
A07
A08
A09
A10
C01
C02
C03
C04
C05
C06
C07
C08
C09
LOD
6
4
2
0
Chromosome
cim
scanone
cim_threshold
scanone_threshold

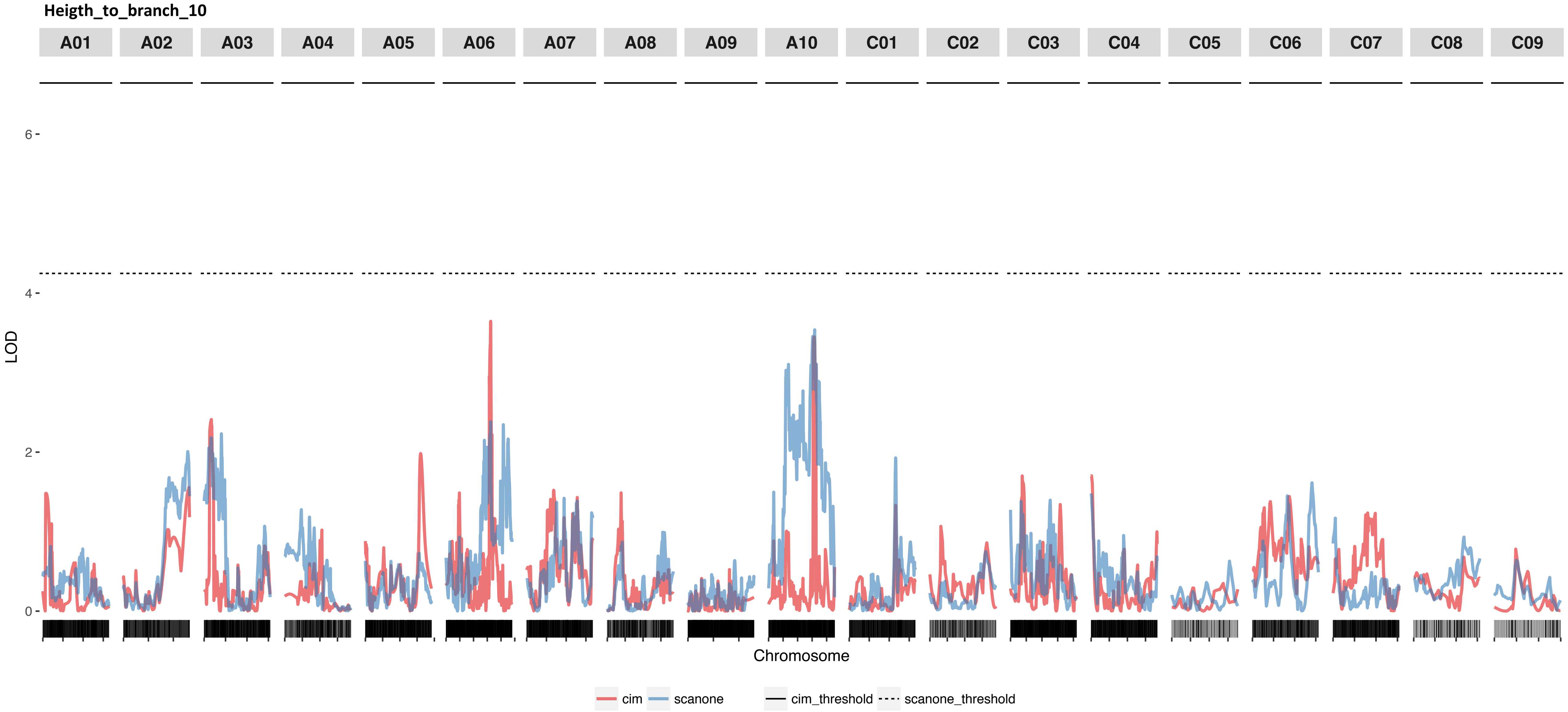

Heigth_to_branch_10
A01
A02
A03
A04
A05
A06
A07
A08
A09
A10
C01
C02
C03
C04
C05
C06
C07
C08
C09
6
4
2
0
LOD
Chromosome
cim
scanone
cim_threshold
scanone_threshold

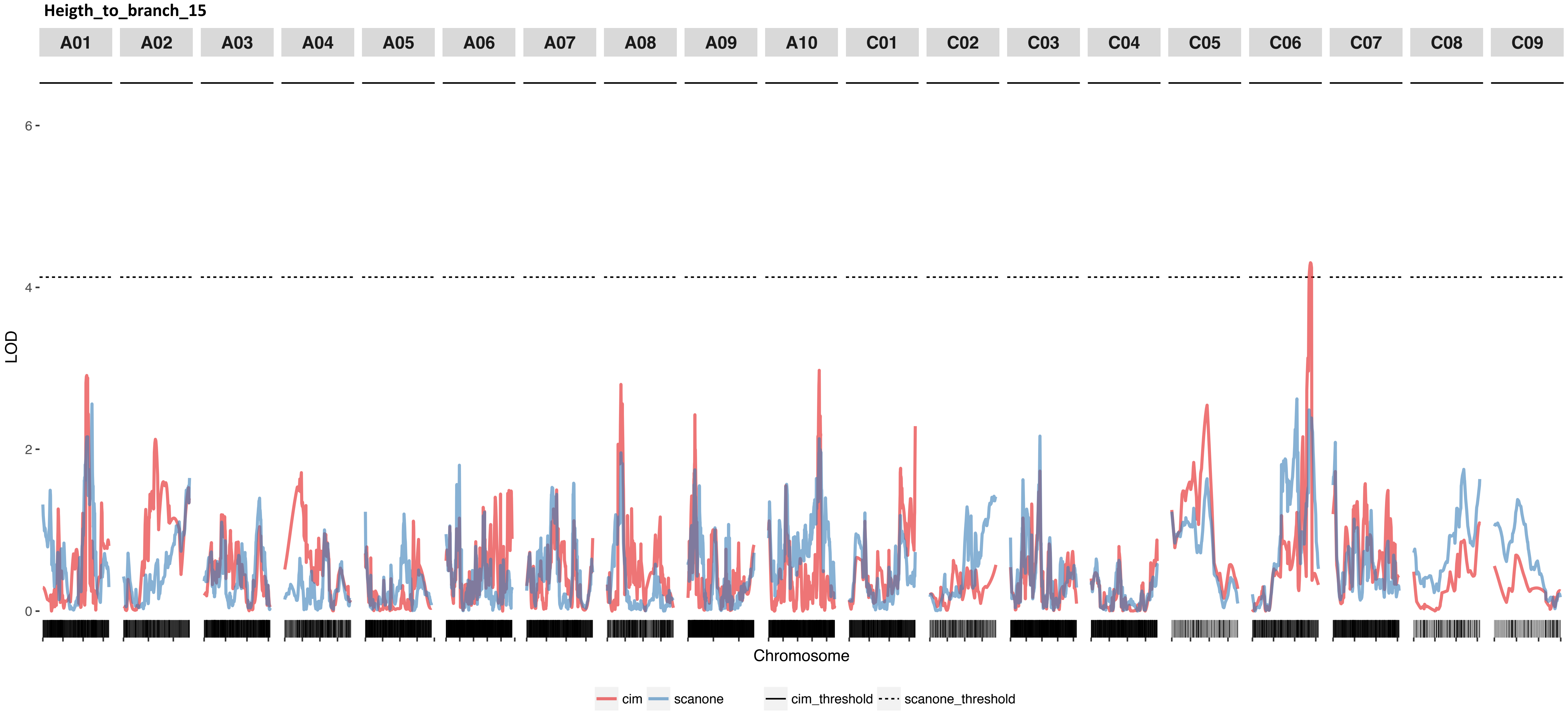

Heigth_to_branch_15
A01
A02
A03
A04
A05
A06
A07
A08
A09
A10
C01
C02
C03
C04
C05
C06
C07
C08
C09
LOD
6
4
2
0
Chromosome
cim
scanone
cim_threshold
scanone_threshold
